# 2024 CSACI Annual Scientific Meeting Book of Abstracts

**DOI:** 10.1186/s13223-025-00971-6

**Published:** 2025-07-31

## Allergic Rhinitis/Asthma

## 1 ACTIONS: chAracterizing oral Corticosteroid use in patients wiTh chronic rhInOsinusitis with Nasal polypS in Canada

### Yvonne Chan^1^, Jeffrey Beach^2^, Jenna Reynolds^2^, Koyo Usuba^3^, Danya Thayaparan^4^, Janine Xu^3^, Ali Tehrani^5^, Juejing Ling^5^, Huijuan Yang^5^, Andrew Thamboo^6^

#### ^1^University of Toronto, Toronto, ON; ^2^Asthma Canada, Toronto, ON; ^3^Health Economics and Outcomes Research, GSK, Mississauga, ON; ^4^Medical Affairs, GSK, Mississauga, ON; ^5^IQVIA Solutions Canada, Inc, Kirkland, QC; ^6^St. Paul’s Hospital, Vancouver, BC

##### **Correspondence:** Yvonne Chan

*Allergy, Asthma & Clinical Immunology* 2025, **21(Suppl 1)**:1

**Background:** Chronic rhinosinusitis with nasal polyps (CRSwNP) is associated with significant disease burden. Oral corticosteroids (OCS) are frequently used to treat CRSwNP; however, OCS therapy is associated with increased risk of adverse events. Biologics are an effective option in managing CRSwNP and reducing need for OCS, yet OCS use prior to biologic initiation is poorly characterized. Therefore, this study aimed to describe patterns of OCS use among patients with CRSwNP in Canada prior to biologic use.

**Methods**: This retrospective, real-world study leveraged IQVIA’s Private Drug Plan claims database. Patients with CRSwNP (diagnosis inferred on longitudinal claims data) 24 months prior to biologic treatment initiation (index) between August 2020 and November 2023 were selected. OCS use and overexposure (total yearly dose of OCS ≥ 1,000 mg [maximum recommended total lifetime cumulative dose]) in the 24 months pre-index (analysis period) were described using mean (± standard deviation [SD]).

**Results:** Overall, 747 patients were included; mean (SD) age was 51 (± 13) years, 48% were female. Over the 24-month analysis period, 81% of patients had ≥ 1 claim for OCS (mean [SD] 4.0 [5.0] claims per patient). Among those with known prescribers, OCS was prescribed by 1, 2 or ≥ 3 physician specialties in 62%, 31% and 7% of patients, respectively. Of those with ≥ 1 claim for OCS, mean (SD) dose per patient was 32 (± 17) mg/day; mean (SD) treatment duration was 77 (± 132) days. In total, 28% of patients had OCS overexposure 12–24 months pre-index, which increased to 39% in the 12 months pre-index.

**Conclusions:** Prior to biologic initiation, 39% of patients with CRSwNP were overexposed to OCS and mean OCS treatment duration reached almost 3 months. Earlier consideration of escalated therapy, adherence to prescribed therapies, and improved communication amongst physicians and patients could reduce risk of OCS overexposure and improve patient care.

## 2 The nasal microbiome of individuals with allergic rhinitis is stable following a nasal allergen challenge with ragweed

### Sophia Linton^1,2^, Jill Greenlaw^1,3^, Calvin Sjaarda^2,4^, Lubnaa Hossenbaccus^1,2^, Jenny Thiele^5^, Lisa Steacy^2^, Prameet Sheth^2,3,4^, Anne Ellis^1,2,5^

#### ^1^Department of Medicine, Queen’s University, Kingston, O; ^2^Kingston General Health Research Institute, Kingston Health Sciences Centre – KGH Site, Kingston, ON; ^3^Gastrointestinal Disease Research Unit, Queen’s University, Kingston, ON; ^4^Department of Pathology and Molecular Medicine, Queen’s University, Kingston, ON; ^5^Department of Biomedical and Molecular Sciences, Queen’s University, Kingston, ON

##### **Correspondence:** Sophia Linton

*Allergy, Asthma & Clinical Immunology* 2025, **21(Suppl 1)**:2

**Background:** Current studies examining the nasal microbiome in allergic rhinitis (AR) often overlook the impact of seasonality and allergen exposure. To address this gap, we analyzed the nasal microbiome of AR participants compared to healthy controls, both before and after exposure to ragweed outside of the pollen season. This study used the validated Allergic Rhinitis Clinical Investigator Collaborative’s nasal allergen challenge (NAC) model.(1).

**Methods**: Nasal sponges were collected at screening and NAC visits (baseline, 6-, 24-, and 48-h post-NAC) to capture the middle meatus microbiome. The variable regions V3-V4 of the bacterial 16S rRNA gene were sequenced using the Illumina MiSeq 2000 platform. Microbial abundance and taxonomic classification were determined using the DADA2 package in R. Differences between groups and times were quantified by alpha diversity (Shannon and Chao1 indices) and beta diversity (principal coordinate analysis based on Bray–Curtis dissimilarity).

**Results:** Samples were collected from 19 AR participants and 15 healthy controls at four time points post-NAC. Analysis revealed no significant changes in alpha or beta diversity between AR and healthy control participants at any time point during the study. However, significant differences in the taxonomic profiles were observed between AR and healthy controls at 24- and 48-h post-NAC (p = 0.008 and p = 0.01491, respectively). Furthermore, when examining the effect of time post-NAC, neither the AR group nor the control group showed significant differences in alpha diversity, beta diversity, or taxonomic profiles.

**Conclusions:** Our study found the nasal microbiome demonstrated remarkable stability with minimal changes observed at 6-h post-NAC. However, significant microbiome changes were observed at 24- and 48-h post-NAC. These findings highlight the importance of further investigating the nasal microbiome over an extended period to understand the long-term effects of NAC.

**References:**

- 1. Ellis AK, Soliman M, Steacy L, Boulay MÈ, Boulet LP, Keith PK, et al. The Allergic Rhinitis – Clinical Investigator Collaborative (AR-CIC): nasal allergen challenge protocol optimization for studying AR pathophysiology and evaluating novel therapies. Allergy Asthma Clin Immunol. 2015 Dec;11(1):1–10.

## 3 Serum cytokine profiles following a cat allergen challenge in the specialized particulate control environmental exposure unit (SPaC-EEU)

### Lubnaa Hossenbaccus^1,2^, Aliya Guttman^1,2^, Sarah Garvey^2^, Terry Walker^2^, Hannah Botting^2^, Lisa Steacy^2^, Anne K. Ellis^1,2^

#### ^1^Department of Medicine, Queen’s University, Kingston, ON; ^2^Allergy Research Unit, Kingston Health Sciences Centre – Kingston General Hospital Site, Kingston, ON

##### **Correspondence:** Aliya Guttman

*Allergy, Asthma & Clinical Immunology* 2025, **21(Suppl 1)**:3

**Background:** The Specialized Particulate Control Environmental Exposure Unit (SPaC-EEU) has been technically and clinically validated for house dust mite allergen, and technically validated for Fel d 1, the major cat allergen [1–2]. We aimed to assess biological outcomes of SPaC-EEU exposure to Fel d 1.

**Methods**: At screening, blood samples were collected from eligible, consenting participants. Qualifying participants returned to the research site for a three hour SPaC-EEU Fel d 1 allergen exposure visit. Blood samples were collected at baseline, 3 h, 6 h (optional), and 24 h post-allergen exposure onset. Samples were processed for serum isolation and frozen. IL-4, IL-5, IL-10, IL-13, IL-25, IL-33, eotaxin-1, MIP-1β, and MCP-1 levels were analyzed with Luminex® xMAP® Technology.

**Results:** Thirty-one Fel d 1-allergic and fifteen non-allergic participants completed this study. Allergic participants experienced a significant increase in IL-5 (p < 0.01), MCP-1 (p < 0.01), and MIP-1β (p < 0.001) between the screening and baseline samples. Eotaxin-1 increased significantly from the baseline to 3-h timepoint (p < 0.05), 3 to 6 h (p < 0.05), and baseline to 6 h (p < 0.001), and decreased from 6 to 24 h (p < 0.05). MCP-1 decreased from baseline to 3 h (p < 0.001) and to 24 h (p < 0.05).

In non-allergic participants, IL-5 decreased significantly from baseline to 6 h and increased from 6 to 24 h (p < 0.05). Eotaxin-1 decreased from 3 to 24 h (p < 0.05) and 6 to 24 h (p < 0.05).

**Conclusions:** Allergic participants exhibited significant biological changes from screening to baseline, indicating fluctuating biological characteristics compared to controls. Furthermore, eotaxin-1 and MCP-1 (both chemoattractants) changed differentially in allergic participants upon Fel d 1 exposure. Going forward, comparing outcomes of those who live with versus without a cat may illustrate the impact of daily Fel d 1 exposure on the allergic state.

**References:**

1. Hossenbaccus L, Linton S, Thiele J, Steacy L, Walker T, Malone C, et al. **Clinical validation of controlled exposure to house dust mite in the environmental exposure unit (EEU).** Allergy Asthma Clin Immunol Off J Can Soc Allergy Clin Immunol. 2021 Mar 26;**17**:34.
2. Hossenbaccus L, Walker T, Linton S, Ellis A. **Reproducibility of the Specialized PArticulate Control Environmental Exposure Unit (SPAC-EEU) as a Novel Controlled Cat Dander Exposure Room**. J Allergy Clin Immunol. 2023 Feb 1;**151**(2):AB105.

## 4 Acuity of pediatric asthma exacerbations in Alberta, Canada is increasing: a retrospective population-based study

### Amanpreet Gill, Jalal Moolji, Adil Adatia

#### University of Alberta, Edmonton, AB

##### **Correspondence:** Amanpreet Gill

*Allergy, Asthma & Clinical Immunology* 2025, **21( Suppl 1)**:4

**Background:** Asthma is the most common chronic disease in children, with an estimated prevalence of 15%^1,2^. The prevalence is increasing worldwide by ~ 50% per decade^3^. Asthma exacerbation is a common reason for ED presentation among children^2,4^. There is a paucity of data on trends in the epidemiology of pediatric asthma exacerbations requiring ED care in Canada.

**Methods**: We conducted a retrospective study examining trends in asthma exacerbation ED visit volumes (normalized for population size), admission rates, and presentation acuity for children ages 0–17 years from 2010 to 2023. Acuity was determined using Canadian Triage and Acuity Scale (CTAS) scores determined by the ED triage nurse at the time of presentation. Data were collected from the Government of Alberta Interactive Health Data Application. Information on CTAS scores was obtained from the Alberta Health Services aggregated administrative database. Data were analyzed graphically.

**Results:** From 2010 to 2023, there were 61,500 ED presentations and 7089 admissions for pediatric asthma exacerbations in Alberta. Additionally, from 2010 to 2023, there was a 64.7% increase in admission rates and a 35.9% increase in ED visits (normalized for population) for asthma across all sexes and age groups. There was a 58.7% drop in asthma ED presentations in 2020 from 2019, likely because of quarantine during the COVID-19 pandemic. There was an increased frequency of CTAS 1 and 2 presentations for both males and females across all age groups.

**Conclusions:** There has been an increase in asthma ED visits, admission rates, and ED presentation acuity in pediatric populations across all sexes and ages from 2010 to 2023.

**References:**

1. Ducharme FM, Dell SD, Radhakrishnan D, et al. Diagnosis and management of asthma in preschoolers: A Canadian Thoracic Society and Canadian Paediatric Society position paper. *Paediatr Child Health* 2015:20(7):353–71.
2. Rosychuk RJ, Voaklander DC, Klassen TP, Senthilselvan A, Marrie TJ, Rowe BH. Asthma presentations by children to emergency departments in a Canadian province: A population-based study. *Pediatr Pulmonol* 2010;45(10):985–92.
3. Ismaila AS, Sayani AP, Marin M, Su Z. Clinical, economic, and humanistic burden of asthma in Canada: A systematic review. *BMC Pulm Med* 2013;13:70.
4. Guttmann A, Zagorski B, Austin PC, et al. Effectiveness of emergency department asthma management strategies on return visits in children: A population-based study. Pediatrics 2007;120(6):e1402-10.

## 5 Association of pulse wave velocity and asthma diagnosis and methacholine challenge

### Douglas Houlbrook^1^, Maryem Zahra^1^, Stephanie Goguen^1^, Scarlet Deluz^1^, Camille Ross^1^,Edmond S. Chan^2^, Allan Becker^1^, Jon McGavock^1^, Elinor Simons^1^

#### ^1^University of Manitoba, Winnipeg, MB; ^2^Division of Allergy, Department of Pediatrics, University of British Columbia and British Columbia Children’s Hospital, Vancouver, BC

##### **Correspondence:** Douglas Houlbrook, Maryem Zahra

*Allergy, Asthma & Clinical Immunology* 2025, **21(Suppl 1)**:5

**Background:** Previous asthma diagnosis has been demonstrated to be a risk factor for adult cardiovascular disease (CVD), measured using pulse wave velocity (PWV). Childhood and adolescent characteristics have been increasingly recognized as possible predictors of chronic diseases. We examined associations between asthma and PWV in the early teen years.

**Methods**: Children born in 1995 in Manitoba were enrolled in a nested case–control birth cohort study, The Study of Asthma, Genes and the Environment (SAGE). At age 12–13 years, participants underwent a clinical assessment to evaluate for asthma, spirometry, methacholine challenge (MCH) and PWV. We used Wilcoxon rank sum to examine PWV (m/s) for children with and without clinical asthma, with PC20 above and below 4 mg/mL and 1 mg/mL cut points, and with FEV1 above and below a cut point of 80%.

**Results:** Of the 288 children with MCH and PWV data, 132 females (46%), and 156 males (54%) completed all measures. PWV (median 7.60, interquartile range 6.80–8.10) was comparable for children with (7.55, 6.70–8.05) and without (7.60, 6.85–8.10) asthma (p = 0.61), and was comparable for PC20 above (7.60, 6.82–8.10) versus at or below (7.55, 6.70–8.10) 1 mg/mL (p = 0.85) or PC20 above (7.60, 6.84–8.10) versus at or below (7.57, 6.74–8.10) 4 mg/mL (p = 0.97). PWV was comparable for children with FEV1 at or above (7.47, 6.75–8.06) versus below (7.78, 7.15–8.15) 80% (p = 0.066). PWV was higher for females with a PC20 above (7.50, 6.75–8.10) versus at or below (7.03, 6.24–7.57) 1 mg/mL, although the association was not significant (p = 0.051).

**Conclusions:** We found no relationship between asthma diagnosis, FEV1 or PC20 and PWV at age 12 years, although examination of adjusted models and asthma at other ages, and further evaluation of gender are warranted.

## 6 Efficacy and safety of the SQ house dust mite SLIT-tablet in children with allergic rhinitis/rhinoconjunctivitis with or without asthma in a randomized controlled trial

### Remi Gagnon^1^, Davide Caimmi^2^, Oliver Pfaar^3^, Majken Hougaard Foss-Skiftesvik^4^, Marie Chantal Arseneault^5^, Hendrik Nolte^6^, Antje Schuster^7^

#### ^1^Clinique spécialisée en allergie de la Capitale, Quebec, QC; ^2^Allergy Unit, CHU de Montpellier and IDESP, UMR A11-INSERM, Université de Montpellier, Montpellier, France; ^3^Department of Otorhinolaryngology, Head and Neck Surgery, Section of Rhinology and Allergy, University Hospital Marburg, Phillipps-Universität Marburg, Marburg, Germany; ^4^ALK, Hørsholm, Denmark, ^5^ALK, Mississauga, ON; ^6^ALK, Bedminster, NJ, USA, 7. Medical Faculty and University Hospital Düsseldorf, Heinrich-Heine-University, Düsseldorf, Germany

##### **Correspondence:** Remi Gagnon, Marie Chantal Arseneault

*Allergy, Asthma & Clinical Immunology* 2025, **21(Suppl 1)**:6

**Background:** Allergic rhinitis/rhinoconjunctivitis (AR/C) induced by house dust mites (HDM) is often associated with concomitant asthma in children. This analysis assessed the efficacy and safety of the SQ HDM sublingual immunotherapy (SLIT)-tablet in children with HDM AR/C with or without concomitant asthma.

**Methods**: Children (N = 1,458) ages 5–11 years with HDM AR/C, with or without controlled asthma (intermittent or mild-to-moderate), were randomized to daily HDM SLIT-tablet (ALK, Denmark) or placebo for ≈52 weeks in a phase 3 double-blind trial (NCT04145219). Children could receive symptom-relieving AR/C medications and asthma rescue medications, if needed. In all, 38% of subjects reported asthma, 55% of whom were using inhaled corticosteroids at baseline. The primary endpoint was the average total combined rhinitis score (TCRS; sum of the rhinitis daily symptom score [DSS] and rhinitis daily medication score [DMS]) during the last 8 weeks of treatment.

**Results:** The TCRS in the overall study population improved with the HDM SLIT-tablet by 22.0% (95% CI: 12.0, 31.1) relative to placebo (p < 0.0001). In subjects with and without asthma, the TCRS improved with the HDM SLIT-tablet by 25.7% (10.3, 38.8; p = 0.002) and 18.9% (5.1, 30.9; p = 0.009) relative to placebo, respectively, the DSS improved by 23.9% (8.4, 37.1; p = 0.004) and 21.2% (9.0, 32.0; p = 0.001), respectively, and the DMS improved by 30.2% (8.5, 47.5; p = 0.009) and 20.3% (-1.7, 38.0; p = 0.068), respectively. The safety profile was similar to the known profile in adults and adolescents. There were no serious treatment-related adverse events (TRAEs), epinephrine administrations, or TRAEs of eosinophilic esophagitis. There were no severe TRAEs in subjects with asthma. The discontinuation rate due to AEs in the HDM SLIT-tablet group was similar in subjects with and without asthma (2.2% and 1.5%, respectively).

**Conclusions:** The HDM SLIT-tablet had a significant clinical effect on AR/C and was well tolerated in children with or without concomitant asthma.

## 7 Efficacy and safety of the SQ tree SLIT-tablet in children with allergic rhinitis and/or conjunctivitis during birch and oak pollen season in a randomized controlled trial

### Remi Gagnon^1^, Oliver Pfaar^2^, Marie Chantal Arseneault^3^, Terrie Dalgaard^4^, Kate Hargreaves^4^, Monika Gappa^5^

#### ^1^Clinique spécialisée en allergie de la Capitale, Quebec, QC; ^2^Department of Otorhinolaryngology, Head and Neck Surgery, Section of Rhinology and Allergy, University Hospital Marburg, Phillipps-Universität Marburg, Marburg, Germany; ^3^ALK, Mississauga, ON; ^4^ALK, Hørsholm, Denmark; ^5^Evangelical Hospital Düsseldorf, Düsselfdorf, Germany

##### **Correspondence:** Remi Gagnon, Marie Chantal Arseneault

*Allergy, Asthma & Clinical Immunology* 2025, **21(Suppl 1)**:7

**Background:** Pollen from birch and other trees in the birch homologous allergen group, including oak, is a common cause of allergic rhinitis and/or conjunctivitis (AR/C) symptoms in Canadian children. This analysis determined the efficacy and safety of the SQ tree sublingual immunotherapy (SLIT)-tablet in children with tree-pollen induced AR/C during the birch and oak pollen seasons.

**Methods**: Children (N = 952) ages 5–17 years with moderate-to-severe tree AR/C were randomized 1:1 to daily tree SLIT-tablet (ALK, Denmark) or placebo for up to 12 months in a double-blind phase 3 trial (EudraCT 2020–004372-17). Subjects had free access to symptom-relieving medication. The primary endpoint was the average total combined score (TCS; sum of rhinoconjunctivitis daily symptom score [DSS] and daily medication score [DMS]) during the birch pollen season (BPS). The average TCS was also analyzed for the oak pollen season (OPS) and the pure OPS where data from days overlapping the birch, alder, or hazel pollen season were excluded.

**Results:** During BPS, the TCS improved by 21.9% (95% CI: 10.6–31.9) with tree SLIT-tablet versus placebo (mean difference = 1.3; p = 0.0004). During OPS, the TCS improved by 25.6% (13.9, 36.0) versus placebo (mean difference = 1.5; p < 0.0001), the DSS improved by 17.8% (5.2, 28.9; p = 0.008), and the DMS improved by 39.9% (23.2, 53.7; p < 0.0001). During pure OPS, the TCS improved by 22.5% (8.5, 34.7) with tree SLIT-tablet versus placebo (mean difference = 1.2; p = 0.0030).

Adverse events were primarily local application site reactions assessed as mild-to-moderate in severity. The rate of discontinuations due to adverse events with the tree SLIT-tablet was 2.7%. One subject experienced symptoms of an anaphylactic reaction within 4 min after the first SLIT-tablet dose, was treated with epinephrine, and recovered within 2 h.

**Conclusions:** The tree SLIT-tablet demonstrated a significant effect in children with AR/C during the birch and oak pollen seasons and was generally well tolerated.

## 8 Impact of early transient increase in eosinophils on the long-term efficacy of dupilumab in patients with moderate-to-severe asthma in TRAVERSE

### Ian D. Pavord^1^, Njira L. Lugogo^2^, Mario Castro^3^, Alberto Papi^4^, Arnaud Bourdin^5^, Michael E. Wechsler^6^, Andréanne Côté^7^, Changming Xia^8^, Mena Soliman^8^, Nami Pandit-Abid^9^, Juby A. Jacob-Nara^9^, Sylvana Sbeinati^10^, Harry Sacks^8^

#### ^1^NIHR Oxford Biomedical Research Centre, University of Oxford, Oxford, United Kingdom; ^2^University of Michigan, Ann Arbor, MI, USA; ^3^Division of Pulmonary, Critical Care and Sleep Medicine, University of Kansas School of Medicine, Kansas City, KS, USA; ^4^Department of Cardiorespiratory Medicine, Respiratory Unit, University of Ferrara, S. Anna University Hospital, Ferrara, Italy; ^5^Department of Respiratory Diseases, PhyMedExp, University of Montpellier, Montpellier, France; ^6^Division of Pulmonary, Critical Care and Sleep Medicine, National Jewish Health, Denver, CO, USA; ^7^Quebec Heart and Lung Institute – Laval University, Quebec, QC; ^8^Regeneron Pharmaceuticals Inc., Tarrytown, NY, USA; ^9^Sanofi, Bridgewater, NJ, USA; ^10^Sanofi, Toronto, ON

##### **Correspondence:** Sylvana Sbeinati

*Allergy, Asthma & Clinical Immunology* 2025, **21(Suppl 1)**:8

**Background:** Early transient increases in blood eosinophils were observed in LIBERTY ASTHMA QUEST (NCT02414854), which together with LIBERTY ASTHMA TRAVERSE (NCT02134028) demonstrated dupilumab efficacy for 148 weeks in patients with moderate-to-severe asthma, with an acceptable safety profile. We assessed the effect of these blood eosinophil increases on treatment outcomes.

**Methods**: Patients from QUEST who enrolled in TRAVERSE were stratified by ≥ twofold or < twofold increase in blood eosinophils at QUEST Week. Unadjusted annualized severe asthma exacerbation rates and change from parent study baseline (PSBL) in prebronchodilator forced expiratory volume in 1 s (FEV_1_) were assessed.

**Results:** At QUEST Week 52, blood eosinophils returned to baseline in both populations (mean [SD] change from PSBL in patients with ≥ twofold increase at Week 12 was 0.241 [0.480] and 0.199 [0.288], or < twofold increase was -0.068 [0.345] and -0.064 [0.336] in dupilumab and placebo groups, respectively). Dupilumab vs placebo reduced asthma exacerbation rates in ≥ twofold-increase (0.437 vs 0.964) and < twofold-increase (0.515 vs 1.072) groups. LS mean difference [95%CI] from PSBL in prebronchodilator FEV_1_ at QUEST Week 52 was numerically higher with dupilumab vs placebo for both ≥ twofold-increase (0.21[0.09–0.33] L; *P* = 0.0008) and < twofold-increase (0.16 [0.11–0.20] L; *P* < 0.0001) groups. At TRAVERSE Week 96, asthma exacerbation rates were reduced (0.283 vs 0.327 and 0.333 vs 0.361 in dupilumab/dupilumab vs placebo/placebo groups, with ≥ twofold and < twofold increase in blood eosinophil count, respectively). Prebronchodilator FEV_1_ improved in the placebo/dupilumab group while improvements were maintained in the dupilumab/dupilumab group (mean [SD] change from PSBL: 0.36 [0.50] vs 0.18 [0.42] L and 0.29 [0.46] vs 0.35 [0.44] L in dupilumab/dupilumab vs placebo/dupilumab groups with ≥ twofold and < twofold increase in blood eosinophil counts, respectively).

**Conclusions:** Dupilumab reduced unadjusted annualized severe asthma exacerbation rates and improved prebronchodilator FEV_1_ up to 148 weeks in patients with asthma, irrespective of transient early increases in blood eosinophils.

## 9 The role of airway epithelial-derived thymic stromal lymphopoietin in regulating the epithelial anti-viral response

### Luke Gerla, Zahraa Yassine-Hojiej, Hazel Marriott, Paige Lacy

#### University of Alberta, Edmonton, AB

##### **Correspondence:** Luke Gerla

*Allergy, Asthma & Clinical Immunology* 2025, **21(Suppl 1)**:9

**Background:** Over 80% of asthma exacerbations are associated with concurrent viral upper respiratory tract infections. It is unclear whether viral infections initiate asthma exacerbations or if asthma exacerbations activate latent viruses present in the lungs. Individuals with asthma have been shown to produce dysregulated anti-viral immune responses causing non-productive inflammation and delayed viral clearance. Airway epithelial cells from donors with asthma have been shown to produce elevated proinflammatory alarmin cytokine expression, including thymic stromal lymphopoietin (TSLP). During virus infection of airway epithelial cells, numerous antiviral genes, including the cytosolic pattern recognition receptor RIG-I, are upregulated. We hypothesize that elevated TSLP released from airway epithelial cells regulates their antiviral responses.

**Methods**: Primary bronchial epithelial cells (NHBE) from healthy donors were grown to 80% confluency before being stimulated with 10 μg/mL of the viral mimetic polyinosinic:polycytidylic acid (poly I:C) in the presence of 25 μg/mL tezepelumab (anti-TSLP) or a commercially available sheep anti-human TSLP and appropriate isotype controls for 8 h. After stimulation, RNA was collected, and gene expression was measured via quantitative polymerase chain reaction (qPCR), normalized to the housekeeping gene GAPDH.

**Results:** Following the 8-h stimulation of NHBE cells with poly I:C alone, a significant increase in the expression of TSLP and the anti-viral gene RIG-I was observed. With the addition of TSLP-blocking antibodies, a numerical decrease in the fold change of TSLP (103 ± 71 to 65 ± 41, mean ± SD) and RIG-I (61 ± 11 to 53 ± 14) was observed when compared to matching isotype controls.

**Conclusions:** This data shows, for the first time, the effect of neutralizing TSLP on the airway epithelial response to a viral mimetic. Therefore, blocking TSLP signalling within the epithelium microenvironment may be an important mechanism in directing the early immune response to viral infections and preventing the initiation of virus-induced asthma exacerbations.

## 10 Development of nasal and ocular symptoms with cat dander exposure in the specialized particulate control environmental exposure unit (SPaC-EEU)

### Lubnaa Hossenbaccus^1,2^, Sarah Garvey^2^, Terry Walker^2^, Hannah Botting^2^, Lisa Steacy^2^, Anne K. Ellis^1,2^

#### ^1^Queen’s University, Kingston, ON; ^2^Allergy Research Unit, Kingston Health Sciences Centre; KGH Site, Kingston, ON

##### **Correspondence:** Sarah Garvey, Lisa Steacy

*Allergy, Asthma & Clinical Immunology* 2025, **21(Suppl 1)**:10

**Background:** The Specialized Particulate Control Environmental Exposure Unit (SPaC-EEU) is a validated cat allergen exposure facility. Here we present ocular symptom outcomes and comparisons with nasal symptoms.

**Methods**: Forty-six successfully screened participants (31 cat-allergic and 15 non-allergic controls) completed the study. They were exposed to cat dander for three hours in the SPaC-EEU and recorded symptom scores at timepoints between baseline to 24 h post-onset of allergen exposure. Nasal symptoms, including runny nose/post-nasal drip, nasal congestion/stuffiness, sneezing, and itchy nose, were compiled as a Total Nasal Symptom Score (TNSS). Total Ocular Symptom Score (TOSS) captured the ocular symptoms of itchy/gritty eyes, watery/tearing eyes, and red/burning eyes. At each timepoint, participants ranked their symptoms on a scale from 0 to 3 for a maximum total of 12 and 9, respectively, for TNSS and TOSS.

**Results:** TOSS was significantly increased (p < 0.05) for allergics compared to non-allergic controls from 30 min to 6 h, peaking at 3 h (mean TOSS = 3.1). Compared to their own baseline, allergic participants experienced significantly increased (p < 0.05) TOSS from 30 min to 4 h. Allergic participants had more pronounced and longer-lasting nasal symptoms than ocular symptoms with cat dander exposure in the SPaC-EEU. Nasal and ocular symptoms for allergics were significantly positively correlated (r = 0.9835, p < 0.0001) and a TOSS of 6 or higher was associated with a minimum TNSS of 3.

**Conclusions:** Allergic participants experienced both nasal and ocular symptoms with cat dander exposure in the SPaC-EEU, though nasal symptoms occurred more rapidly and with greater magnitude. The development of ocular symptoms with this controlled model mimics real-life cat exposure, confirming the utility of the SPaC-EEU for studying rhinoconjunctivitis.

## 11 Initiation of dupilumab led to reduced corticosteroid and antibiotic use over 12 months in patients with chronic rhinosinusitis with nasal polyps in US real-world practice

### Stella E. Lee^1^, Joseph K. Han^2^, Joshua M. Levy^3^, Zachary M. Soler^4^, Matthew Chow^5^, Lucia De Prado Gomez^6^, Nehal Kamal^7^, Scott Nash^8^, Mark Corbett^9^, Amr Radwan^10^, Juby A. Jacob-Nara^9^

#### ^1^Division of Otolaryngology — Head & Neck Surgery, Brigham and Women’s Hospital, Harvard Medical School, Boston, MA, USA; ^2^Department of Otolaryngology and Head and Neck Surgery, Eastern Virginia Medical School, Norfolk, VA, USA; ^3^National Institute on Deafness and Other Communication Disorders, NIH, Bethesda, MD, USA; ^4^Department of Otolaryngology – Head and Neck Surgery, Medical University of South Carolina, Charleston, SC, USA; ^5^Sanofi, Mississauga, ON, ^6^Sanofi, Madrid, Spain; ^7^Sanofi, Jeddah, Saudi Arabia; ^8^Regeneron Pharmaceuticals Inc., Tarrytown, NY, USA; ^9^Sanofi, Bridgewater, NJ, USA; ^10^Regeneron Pharmaceuticals Inc., Uxbridge, United Kingdom

##### **Correspondence:** Matthew Chow

*Allergy, Asthma & Clinical Immunology* 2025, **21(Suppl 1)**:11

**Background:** Dupilumab improved outcomes in patients with severe chronic rhinosinusitis with nasal polyps (CRSwNP) in randomized clinical trials and in real-world practice. This study compared the burden of corticosteroid and antibiotic use in patients with CRSwNP before and after initiation of dupilumab in the US, where there is currently limited evidence on dupilumab effectiveness.

**Methods**: A retrospective observational cohort study in adults with CRSwNP who initiated dupilumab 300 mg every 2 weeks between June 2019 and June 2022. Data from the OM1 Real-World Data Cloud and the Reg-ENT Registry were used. In this analysis, CRSwNP-related use of oral corticosteroids (OCS) was defined as within 5 days of a CRS and/or NP diagnosis or within 30 days of a sinus surgery. CRSwNP-related OCS use and prescription of antibiotics in the 12 months before and after initiation of dupilumab (pre- and post-dupilumab periods, respectively) are summarized descriptively.

**Results:** The cohort comprised 1016 patients. The proportion of patients with CRSwNP-related OCS use decreased from 59.1% in the pre-dupilumab period to 17.7% in the post-dupilumab period; 76.5% of patients with CRSwNP-related OCS use pre-dupilumab discontinued OCS use post-dupilumab. CRSwNP-related OCS cumulative number of prescriptions/fills reduced from 553 in the pre-dupilumab period to 132 in the post-dupilumab period (76.1% decrease). The proportion of patients with a prescription for antibiotics reduced from 64.6% in the pre-dupilumab period to 31.8% in the post-dupilumab period. Among patients with comorbid asthma (n = 579 [57.0%]), CRSwNP-related OCS use decreased from 58.7% in the pre‑dupilumab period to 18.7% in the post‑dupilumab period.

**Conclusions:** Patients with CRSwNP who initiated dupilumab had reduced corticosteroid and antibiotic use during the 12 months following dupilumab initiation compared with the 12 months pre-dupilumab, supporting the real-world effectiveness of dupilumab in the US.

## 12 Baseline characteristics of patients with chronic rhinosinusitis with nasal polyps and coexisting asthma initiating dupilumab in the AROMA global registry

### Enrico Heffler^1^, Tanya M. Laidlaw^2^, Shigeharu Fujieda^3^, Matthew Chow^4^, Scott Nash^5^, Changming Xia^5^, Micah Johnson^5^, Lucia De Prado Gomez^6^, Paul J. Rowe^7^, Yamo Deniz^5^, Juby A. Jacob-Nara^7^, Amr Radwan^8^

#### ^1^Personalized Medicine, Asthma and Allergy, Humanitas Research Hospital, Milan, Italy; ^2^Brigham and Women’s Hospital, Harvard Medical School, Boston, MA, USA; ^3^Department of Otorhinolaryngology, Head and Neck Surgery, University of Fukui, Fukui, Japan; ^4^Sanofi, Mississauga, ON; ^5^Regeneron Pharmaceuticals Inc., Tarrytown, NY, USA; ^6^Sanofi, Madrid, Spain; ^7^Sanofi, Bridgewater, NJ, USA; ^8^Regeneron Pharmaceuticals Inc., Uxbridge, United Kingdom

##### **Correspondence:** Matthew Chow

*Allergy, Asthma & Clinical Immunology* 2025, **21(Suppl 1)**:12

**Background:** Dupilumab is approved for patients with uncontrolled chronic rhinosinusitis with nasal polyps (CRSwNP). Patients with CRSwNP and coexisting asthma have been reported previously to have more severe CRSwNP and more frequent asthma exacerbations. This analysis describes the baseline characteristics of patients initiating dupilumab for CRSwNP in the AROMA prospective global registry study (NCT04959448).

**Methods**: AROMA is enrolling adult patients with CRSwNP initiating dupilumab and following them for up to 36 months. Baseline assessments include demographics, disease characteristics, and history of coexisting type 2 inflammatory diseases including asthma.

**Results:** As of February 2023, the study had enrolled 303 patients; 214 (70.6%) had a history of asthma and 210 (69.3%) had ongoing asthma at baseline. Of the 214 with a history of asthma, the mean (SD) age of asthma diagnosis was 36.6 (17.3) years. In the year prior to screening, the mean (SD) number of systemic corticosteroid treatment days for severe asthma exacerbation was 2.9 (9.56) days and the mean (SD) number of days of hospitalization for severe asthma was 0.1 (0.37). Among the entire AROMA population, baseline mean (SD) 6-item Asthma Control Questionnaire score (n = 168) was 1.36 (1.20) and mean (SD) Mini Asthma Quality of Life Questionnaire (n = 168) domain scores were: symptoms, 4.99 (1.45); activity limitation, 5.60 (1.34); emotional function, 5.21 (1.53); environmental stimuli, 4.95 (1.61). Mean (SD) fractional exhaled nitric oxide (n = 51) was 53.3 (63.8) ppb. The mean percent predicted forced expiratory volume in 1 s (FEV_1_) was 88.8%, and 92.9% of patients were ≥ 60% predicted FEV_1_.

**Conclusions:** More than two-thirds of adult patients with CRSwNP initiating dupilumab in the Global AROMA Registry have coexisting asthma, some of whom reported use of systemic corticosteroids for severe asthma exacerbations.

## 13 The role of synthetic surfactant on sleep apnea in a Brown Norway Rat model of asthma

### Mustafa R. Al-Saiedy, Andrea Chiu, Evan Nelson, Francis Green

#### University of Calgary, Calgary, AB

##### **Correspondence:** Mustafa R. Al-Saiedy

*Allergy, Asthma & Clinical Immunology* 2025, **21(Suppl 1)**:13

**Background:** Sleep apnea is a breathing disorder characterized by cessation in breathing or shallow and infrequent breathing during sleep (1). Asthma and OSA often coexist, with OSA being more common in asthmatics and associated with asthma severity and control (2,3). Brown Norway rats (BN) serve as models for studying both sleep apnea and asthma. Inhaled 8% CO2 combined with nebulized surfactant (Perflubron) has shown promise in dilating airways and reducing lung resistance in animal models of chronic allergic asthma, suggesting a potential new approach for treating sleep apnea.

**Methods**: BN rats were sensitized to ovalbumin (OVA), receiving 1 mL intraperitoneal injection of a solution containing 100μL OVA, 1.5 g Al(OH)3, and 10μL Bordetella pertussis toxin in 10 mL of 0.9%NaCl. On the 21st, 28th, and 35th days post-sensitization, rats were exposed to nebulized OVA for 10 min in a 15 L chamber. After exposure, rats stayed in the chamber for 15 min before transferral to a whole-body plethysmograph for pulmonary function testing. All rats had a minimum one-week washout period between OVA challenges. Different treatments were administered 1 week after the sensitization/washout period. The study received ethics approval, protocol MO9101.

**Results:** The response to Perflubron-8% CO2 treatment was remarkably rapid, commencing immediately upon administration of the treatment, and persisting throughout the 10-min treatment period and for 5 min post treatment. Both the number and duration of apneas in animals treated with Perflubron-8 exhibited significant improvement from those treated with other combinations and medical air.

**Conclusions:** The current study demonstrated that inhaled Perflubron combined with 8% CO2 is a potent formulation for reducing apneic events in both number and duration following OVA allergen challenge in the BN rat model of allergic asthma. However, this treatment effect is relatively short-lived, except at the higher doses.

**References:**

1. *Khan MT, Franco RA. Complex sleep apnea syndrome. Sleep Disord. 2014;2014:798,487.*
2. Prasad B, Nyenhuis SM, Weaver TE. Obstructive sleep apnea and asthma: associations and treatment implications. Sleep Med Rev. 2014 Apr;18(2):165–71.
3. Prasad B, Nyenhuis SM, Imayama I, Siddiqi A, Teodorescu M. Asthma and Obstructive Sleep Apnea Overlap: What Has the Evidence Taught Us? Am J Respir Crit Care Med. 2020 Jun 1;201(11):1345–57.

## 14 Factors associated with epinephrine administration in pediatric anaphylaxis

### Nuha M. Abdulhaq^1^, Basim Alsaywid^2^, Connor Prosty^3^, Christine McCusker^1^, Adam Bretholz^4^, Ann E. Clarke^5^, Mohammed Kaouache^1^, Judy Morris^5^, Rodrick Lim^6^, Edmond Chan^7^, Ran Goldman^8^, Andrew O’ Keefe^9^, Jennifer Gerdts^10^, Julia Upton^11^, Elana Hochstadter^12^, Jocelyn Moisan^13^, Xun Zhang^14^, Jennifer Protudjer^15^, Elissa Abrams^16^, Elinor Simons^16^, Shira Benor^17^, Moshe Ben-Shoshan^1,3^

#### ^1^Department of Pediatrics, Division of Allergy Immunology, McGill University Health Centre, Montreal, Canada. Canada, Montreal, QC; ^2^Department of Education and Research Skills Directory, Saudi National Institute of Health, Riyadh, Saudi Arabia, Riyadh, Saudi Arabia; ^3^Faculty of Medicine, McGill University, Montreal, Montreal, QC; ^4^Department of Pediatrics, McGill University Health Centre, Montreal, Canada, Montreal, QC; ^5^Department of Medicine, Division of Rheumatology, Cumming School of Medicine, University of Calgary, Calgary, Canada, Calgary, AB; ^6^Division of Pediatric Emergency Medicine, Department of Pediatrics, Children’s Hospital at London Health Science Centre, London, Ontario, Canada, London, ON; ^7^Division of Allergy and Immunology, Department of Pediatrics, BC Children’s Hospital, University of British Columbia, Vancouver, British Columbia, Canada, Vancouver, BC; ^8^Division of Clinical Pharmacology and Emergency Medicine, Department of Pediatrics, BC Children’s Hospital, University of British Columbia, Vancouver, British Columbia, Canada, Vancouver, BC; ^9^Department of Pediatrics, Faculty of Medicine, Memorial University, St John’s, Newfoundland & Labrador, Canada, St John’s, NL; ^10^Executive Director, Food Allergy Canada, Toronto, Ontario, Canada, Toronto, ON; ^11^Division of Immunology and Allergy, Department of Pediatrics, The Hospital for Sick Children, Department of Paediatrics, University of Toronto, Toronto, ON, Canada, Toronto, ON; ^12^Department of Pediatric Emergency Medicine, The Hospital for Sick Children, University of Toronto, Ontario, Canada, Toronto, ON; ^13^Regional Medical Director of Emergency Medical Services of Outaouais, Outaouais, Quebec, Canada, Outaouais, QC; ^14^Centre for Outcomes Research and Evaluation, Research Institute of McGill University Health Centre, Montreal, Quebec, Canada, Montreal, QC; ^15^Department of Pediatrics and Child Health, Children’s Hospital Research Institute of Manitoba, Winnipeg, Manitoba, Canada, Winnipeg, MB; ^16^Department of Pediatrics and Child Health, Section of Allergy and Clinical Immunology, Children’s Hospital Research Institute of Manitoba, Winnipeg, Manitoba, Canada, Winnipeg, MB; ^17^Allergy and Clinical Immunology Unit, Dana-Dwek Children’s Hospital, Tel Aviv Medical Center, Tel Aviv University, Tel Aviv, Israel, Tel Aviv, Israel

##### **Correspondence:** Nuha M. Abdulhaq

*Allergy, Asthma & Clinical Immunology* 2025, **21(Suppl 1)**:14

**Background:** This study aims to examine the relationship between triggers, reaction type, severity, and location, and the total doses of epinephrine given.

**Methods**: This is a cross-sectional study utilizing data from the Cross Canada Anaphylaxis Registry. The study examined epinephrine administration and factors associated with it in pediatric patients with anaphylaxis. Data analysis involved descriptive statistics, bivariate analysis, and statistical tests to identify significant associations.

**Results:** A total of 4,469 registered anaphylaxis events from the Cross Canada Anaphylaxis Registry were assessed between 2011 and 2024. The majority of events occurred in male pediatric patients (59.7%), with a median age of 5.5 years, compared to female patients (40.3%) with a median age of 7.9 years (p < 0.0001).

In terms of epinephrine administration during anaphylaxis events, a significant proportion required only one dose (60.6%), while a smaller percentage required multiple doses. Patients with known food allergies and asthma were more likely to require multiple epinephrine doses (p < 0.0001 and p = 0.014, respectively).

The location of reaction influenced the dosage of epinephrine, with children experiencing reactions at school or daycare centers being administered more doses (p < 0.0001). The most common triggers identified were peanut, tree nut, egg, and milk. Peanut-triggered reactions were more likely to be treated with more epinephrine doses (p < 0.0001).

Hospital admission rates were relatively low (3.4%), but patients who received three or more doses of epinephrine were more likely to be admitted, with higher rates observed for those who received four or more doses (p < 0.0001). Furthermore, a small percentage of our cohort (0.8%) required ICU admission, with significantly higher rates observed in patients who received multiple epinephrine doses (p < 0.0001).

**Conclusions:** Our study highlights the need for personalized management strategies in pediatric anaphylaxis, considering triggers, location, and individual patient characteristics to optimize epinephrine dosing and enhance patient outcomes.

## 15 Incorporating weight management in routine asthma care, a pilot study reporting on the correlation of poor asthma control and increased BMI

### Michail Staroselskiy^1,2^, Zobia Nawaz^1^, Tasnia Salim^1^, Naila Ali^1^, Reshma Rasheed^1^

#### ^1^Chapel Street Surgery UK, Billericay, United Kingdom; ^2^Kharkiv National Medical University, Kharkiv, Ukraine

##### **Correspondence:** Michail Staroselskiy

*Allergy, Asthma & Clinical Immunology* 2025, **21(Suppl 1)**:15

**Background:** Prevalence rates of asthma and obesity are rising nationally and internationally. Obesity adversely affects asthma control and outcomes. Weight management is not a feature of Canadian, American, European, Scottish, United Kingdom and Global guidelines as part of routine care. Despite the known association none of the aforementioned guidelines advocate weight optimisation as routine best practice. This analysis was undertaken as a pilot for a quality improvement weight reduction initiative for asthma patients with high BMI.

**Methods**: Electronic patient records of asthmatic patients (n = 272) were analysed for worsening Asthma Control Test (ACT) scores with rising Body Mass Index (BMI), and concomitant asthma treatment escalation most likely due to the raised BMI.

**Results:** We found patients with BMI > 25 had a 72.28% average to have either an increased risk of exacerbation, lower ACT scores or be on Long-acting beta agonists and inhaled corticosteroids. The first audit cycle showed of 272 asthma patients, 68.75% had asthma with a BMI > 25 with a mean average of 27.41 BMI. Out of the 272 patients 36.39% had a BMI > 30 with a mean average of 35.27 BMI. These patients were offered referral to weight management.

**Conclusions:** Holistic management of patients will lead to improved long-term outcomes, mental health and QoL scores. This quality improvement initiative offering patient education and weight management strategies is now incorporated into routine care. We believe weight optimisation should be offered as part of routine asthma care and clinician awareness, this will improve asthma outcomes for patients.

## 16 Nasal epithelial expression of alarmin cytokines in response to biologic treatment in asthma

### Hazel Marriott^1^, Marc Duchesne^1^, Luke Gerla^1^, Isobel Okoye^2^, Irvin Mayers^1^, Jalal Moolji^1^, Adil Adatia^1^, Paige Lacy^1^

#### ^1^Division of Pulmonary Medicine, Department of Medicine, University of Alberta, Edmonton, AB; ^2^School of Interdisciplinary Science, McMaster University, Hamilton, ON

##### **Correspondence:** Hazel Marriott

*Allergy, Asthma & Clinical Immunology* 2025, **21(Suppl 1)**:16

**Background:** The secretion of epithelial alarmin cytokines, thymic stromal lymphopoietin (TSLP), interleukin (IL)-25, and IL-33, heralds the onset of the asthma inflammatory cascade and immune effector infiltration. Recently, our group has shown that TSLP and IL-25 are elevated in nasal epithelia of severe asthmatics. We hypothesize that nasal alarmin cytokine expression decreases in response to biologic therapy.

**Methods**: Nasal epithelial cells derived from nasal brushings were collected from severe asthmatics identified as candidates for biologic therapy upon presentation to clinic. Patients were followed up at 3 months after initial presentation. Biologic therapy included mepolizumab, benralizumab, and tezepelumab administered according to clinical guidelines. Intracellular expression of alarmin cytokines was assessed by flow cytometry using nasal brushing cells immunolabeled with antibodies against TSLP, IL-25, IL-33, and cytokeratin 8, an epithelial cell marker, and analyzed using a Fortessa-SORP cytometer with FlowJo version 10.10. Clinical characteristics including spirometry, fractional exhaled nitric oxide (FeNO), and asthma control questionnaire (ACQ)-6 score were collected at each visit.

**Results:** At the 3 month follow-up visit, patients who had received biologics were found to have a reduction in nasal TSLP, IL-25 and IL-33 expression with mean fold changes of ‑3.19 ± 2.17, ‑2.81 ± 1.28 and ‑2.43 ± 1.05 (mean ± SD, *n* = 4), respectively. These changes were associated with an average improvement in ACQ-6 score of 1.01 ± 1.24 (*n* = 4) and a reduction in FeNO of 38 ppb (*n* = 2). No clinically relevant changes were observed in peripheral blood eosinophils or spirometry values.

**Conclusions:** Patients responsive to biologic therapy demonstrated reduced expression of TSLP, IL-25, and IL-33 in nasal brushings as early as 2 doses after initiation of therapy, suggesting nasal cytokine expression may provide insight into early patient responses to biologics. Further work is ongoing with more patients to confirm these findings.

## 17 Investigating mast cell-epithelial crosstalk and mucus plugging in severe asthma

### Yinglan Xie^1^, Lise L. Eriksen^3^, Xiaotian Ju^1^, Swasthika Swaminathan^1^, Melanie Kjarsgaard^2^, Lisa Harper^2^, Parameswaran Nair^1^, Celeste Porsbjerg^3^, Manali Mukherjee^1^

#### ^1^Department of Medicine, McMaster University, Hamilton, ON; ^2^Research Institution of St. Joseph’s Healthcare Hamilton, Hamilton, ON; ^3^Department of Lung and Infectious Diseases, Copenhagen, Denmark

##### **Correspondence:** Yinglan Xie

*Allergy, Asthma & Clinical Immunology* 2025, **21(Suppl 1)**:17

**Background:** Mast cell (MC)-epithelial interactions and their effect on mucus plugging is less known. We recently reported tryptase (MC degranulation marker) in severe asthma sputa suggesting submucosal MCs may underlie airway inflammation [1]. We hypothesized that increased MCs degranulation activates epithelium and impact mucin production.

**Methods**: MC cell-line LAD2 (T8157) was validated for tryptase release using prototypical MC activators 10ug/mL C48/80 and 0.5ug/mL IgE. Calu-3 cells (HTB-55) were differentiated to pseudo-stratified epithelium in air–liquid interface (ALI) for 19 days before being co-cultured ± stimulants or with supernatants from degranulated LAD2 cells for 48 h. The 21-day ALI monolayers were assessed for intracellular mucin (MUC5AC, MUC5B) and alarmins (TSLP, IL-33, IL-25) within the total epithelial cells (EpCAM^+^), secretory cells (EpCAM^+^TUBA^−^ CD271^−^) and goblet cells (EpCAM^+^TUBA^−^CD271^−^CD66c^+^TSPAN8^+^) by flow cytometry, further confirmed by ELISA (basolateral supernatant). Apical fluid was evaluated for mucin protein content (MUC5AC/5B, #NBP2-76,703, #NBP2-76,705, NovusBiologicals).

**Results:** LAD2 cells express tryptase (by immunostaining with TPSAB antibody) and secrete upon stimulation with C48/80 and IgE. An increase in goblet (33%) and secretory (26%) cells population was observed following co-culture with both intact MC and degranulated MCs *vs.* untreated. MUC5AC^+^ and MUC5B^+^ secretory (23%, 35%) and goblet cells (29%, 18%) were increased by IgE-stimulated MCs vs untreated. Indeed, in the apical fluid there was an increased detection of MUC5AC (2.81 ± 0.74 vs 5.17 ± 1.09 ng/mL/ug protein, p = 0.03) and MUC5B (10.15 ± 4.48 vs 18.58 ± 1.98 ng/mL/ug protein, p = 0.04). IgE-induced MC-degranulation caused increase in intracellular expression of TSLP + (33%), IL-33 + (34%), IL-25 + (13%) goblet cells and TSLP^+^ secretory cells (23%) compared to control.

**Conclusions:** To our knowledge this is first study to report MC degranulation directly increases the secretion of both mucins and alarmins directly in a human bronchial epithelial ALI model. Though preliminary, the result warrant a thorough investigation into the role of MCs in epithelial activation and subsequent mucus hypersecretion.

**References:**

1. Kjarsgaard M, Radford K, Zhang K, Huang C, Lavigne N, Mukherjee M, et al. Sputum Tryptase in Patients With Moderate to Severe Asthma. A31 GLOBAL AND ENVIRONMENTAL INFLUENCES IN ASTHMA. American Thoracic Society; 2023. p. A1301–A1301. https://www.atsjournals.org/doi/10.1164/ajrccm-conference.2023.207.1_MeetingAbstracts.A1301

## 18 Nasal gene expression of IL-4 is elevated in house dust mite-allergic participants after allergen exposure in the Environmental Exposure Unit

### Rachel Lucyshyn^1^, Lubnaa Hossenbaccus^2,3^, Cortney Haird^3,4^, Anne K. Ellis^2,3,4^

#### ^1^Department of Medicine, University of Ottawa, Ottawa, ON; ^2^Department of Medicine, Queen’s University, Kingston, ON; ^3^Allergy Research Unit, Kingston Health Sciences Center—KGH Site, Kingston, ON; ^4^Department of Biomedical and Molecular Sciences, Queen’s University, Kingston, ON

##### **Correspondence:** Rachel Lucyshyn.

*Allergy, Asthma & Clinical Immunology* 2025, **21(Suppl 1)**:18

**Background:** We previously investigated participants with house dust mite (HDM) allergic rhinitis (AR) exposed to airborne HDM (modest or higher target concentrations) for three hours in the Specialized Particulate Control Environmental Exposure Unit. Allergic participants demonstrated significant differences in serum cytokine concentrations and significantly higher symptom scores compared to non-allergic participants [1,2]. The current project aimed to assess interleukin *IL)-4* and *IL-25* nasal gene expression in the same participants to explore whether previous findings are reflected transcriptionally and further understand HDM-AR pathophysiology.

**Methods**: Nasal brushing samples were collected 1–2 weeks pre-exposure and immediately post-exposure during the aforementioned study [1,2]. Ribonucleic acid was isolated from nasal epithelial cells then reverse transcribed to complementary DNA. Quantitative polymerase chain reaction with relevant controls determined relative normalized expression (&Delta;&Delta;Cq) of gene targets *IL-4*, *IL-25*, and reference gene *ubiquitin C*. Expression was compared between allergic or non-allergic status, pre- or post-exposure, and modest or higher HDM target concentrations. Correlations with serum IL-4 and IL-13 concentrations and symptom scores were explored.

**Results:** Samples from 41 allergic and 10 non-allergic participants were evaluated. Allergic participants showed significantly higher post-exposure *IL-4* expression compared to non-allergic participants (p = 0.0235) and to pre-exposure (p = 0.0244). *IL-25* expression was not significantly different based on allergic status nor timepoints. In allergic participants, change in gene expression for both targets were not significantly different between HDM concentrations. *IL-4* expression was significantly negatively correlated (r = -0.8986, p = 0.0278) with serum IL-4 concentrations and *IL-25* expression was significantly positively correlated (r = 0.4171, p = 0.0426) with serum IL-13 concentrations.

**Conclusions:** *IL-4* expression demonstrated significant differences between allergic status and timepoints, confirming involvement in the nasal HDM-AR response. *IL-25* expression did not show significant differences between conditions. Correlations between expression and certain serum cytokines could speak to cytokine pathways and tissue differences.

**References:**

1. *Hossenbaccus L, Linton S, Thiele J, Steacy L, Walker T, Malone C, *et al*. Clinical validation of controlled exposure to house dust mite in the environmental exposure unit (EEU). Allergy Asthma Clin Immunol2021; 17(1):34.*
2. Hossenbaccus L, Linton S, Thiele J, Steacy L, Walker T, Malone C, et al. **Biological Responses to House Dust Mite Exposure in the Environmental Exposure Unit** *J Allergy Clin Immunol.* 2022; **149**(2):AB22

## 19 Update on the observed incidence of anaphylaxis and serum sickness in patients receiving omalizumab in a Canadian tertiary allergy/asthma clinic

### Stephanie Santucci^1^, Andrew Comiskey^2,4^, Timothy Olynych^1,5^, Duazylle Torres^3^, Hazelyn Torres^3^, Suzanne Kelly^2^, Rachel Friedrich^2^, William Yang^1,3,5^

#### ^1^Yang Medicine, Ottawa, ON; ^2^Red Maple Trials Inc, Ottawa, ON; ^3^Ottawa Allergy Research Corporation, Ottawa, ON; ^4^Emory University, Atlanta, GA, USA; ^5^University of Ottawa, Ottawa, ON

##### **Correspondence:** Stephanie Santucci

*Allergy, Asthma & Clinical Immunology* 2025, **21(Suppl 1)**:19

**Background:** Omalizumab is a recombinant humanized mAb against IgE. It is approved for the treatment of severe allergic asthma, severe chronic spontaneous urticaria and more recently chronic rhinosinusitis with nasal polyps. Omalizumab binds circulating IgE (96%) regardless of specificity. It forms small biologically inert omalizumab/IgE complexes. These may activate the complement system.

In post-marketing analyses, FDA reported an estimated incidence of anaphylaxis of 0.2%. In 2007, the FDA issued a black-box warning regarding the potential risk of anaphylaxis. Omalizumab Joint Task Force (OJT) reported an anaphylaxis incidence of 0.09%; clinical experience reported 0.1% incidence of anaphylaxis.

**Methods**: In our tertiary clinic specializing in allergy and asthma, we administer omalizumab to patients who meet specific criteria. Asthma patients typically have elevated IgE levels between 30–700 IU/L (adults), 30–1300 IU/L (adolescents > 6-year-old), significant airway reversibility of > 12%, require frequent oral prednisone treatment and have had multiple emergency room visits. Additionally, we administer omalizumab to patients with severe chronic spontaneous urticaria (CSU), who have not responded to conventional H1-antihistamines three to four times the recommended dose, or are dependent on oral steroids.

These patients were closely monitored. Asthmatic patients underwent spirometry testing every three months, and CSU patients reporting UAS7 scores every six months. This regular assessment was conducted to guarantee the safety and efficacy of the treatment being administered.

**Results:** In our tertiary clinic, we have administered approximately more than 2.5 million omalizumab injections in the past two decades without any reported anaphylaxis requiring medical intervention or serum sickness.

**Conclusions:** In summary, omalizumab exhibits a high level of safety and efficacy, with no risk of anaphylaxis or serum sickness.

## Allied Health

## 20 Global Access to Psychological Services for food allergy (GAPS) – Canadian focus

### Zoe Harbottle^1,2,3^, Cathérine Lemoine-Courcelles^1,2^, Jennifer D. Gerdts^4^, Mary Jane Marchisotto^5^, Helen A. Brough^6,7^, Christina J. Jones^8^, Linda J. Herbert^9,10^, Rebecca C. Knibb^11^, Jennifer L. Protudjer^1,2,12^

#### ^1^Department of Pediatrics and Child Health, University of Manitoba, Winnipeg, MB; ^2^Children’s Hospital Research Institute of Manitoba, Winnipeg, MB; ^3^Department of Food and Human Nutritional Sciences, University of Manitoba, Winnipeg, MB; ^4^Food Allergy Canada, Toronto, ON; ^5^MJM Advisory LLC, New York, NY, USA; ^6^Children’s Allergy Service, Evelina London Children’s Hospital, Guy’s and St Thomas’ NHS Foundation Trust, London, United Kingdom; ^7^Paediatric Allergy Group, Department of Women and Children’s Health, School of Life Course Sciences, King’s College London, London, United Kingdom; ^8^School of Psychology, Faculty of Health & Medical Sciences, University of Surrey, Surrey, United Kingdom; ^9^Division of Psychology & Behavioural Health, Children’s National Hospital, Washington, DC, USA; ^10^Department of Pediatrics, George Washington University School of Medicine, Washington, DC, USA; ^11^Institute of Health and Neurodevelopment, Aston University, Birmingham, United Kingdom; ^12^Institute of Environmental Medicine, Karolinska Institutet, Stockholm, Sweden

##### **Correspondence:** Zoe Harbottle

*Allergy, Asthma & Clinical Immunology* 2025, **21(Suppl 1)**:20

**Background:** Food allergy has been associated with increased stress and anxiety and reduced quality of life in both those with food allergy and their caregivers. Given these findings, Phase II of the Global Access to Psychological Services (GAPS) Study aimed to qualitatively explore the psychological implications of food allergy and to understand what is necessary in terms of psychological support and resources to best help caregivers of children, and adults with food allergy. Herein, we report on findings from Canada.

**Methods**: In this phase of GAPS, caregivers of children with food allergy and adults with food allergy were recruited via patient organizations, social media, and email follow-up with participants from the first phase of the study who consented to be contacted for future phases. Semi-structured interviews were conducted to explore participants psychological service needs. All interviews were audio recorded, transcribed verbatim, and analyzed thematically. Aston University Research Ethics Board approved this study.

**Results:** Thirty-three interviews were completed, including 16 with caregivers, and 17 with adults with food allergy. On average, caregivers were 44.9 ± 6.7 years old and adults were 32.2 ± 12.5 years old. Four themes were identified: food allergy anxiety remains present in all life stages; food allergy is perceived to limit opportunities; allied healthcare: an important piece in the food allergy management team; and multimodal approach to support.

**Conclusions:** Although anxiety is a constant in the lives of many managing food allergy, appropriate supports are lacking. There is a need for access to allied healthcare professionals including psychologists and dietitians specializing in food allergy. In addition to professional guidance, peer support is also perceived to be necessary. To be effective for all individuals, resources are desired in a variety of formats. The need for support increases during periods where anxiety may be exacerbated.

## 21 An update on the CSACI food allergy educator program

### Jennifer L. Protudjer^1^, Douglas P. Mack^2^, Lori Connors^3^, Jasmin Lidington^4^, Harold Kim^5^

#### ^1^University of Manitoba, Winnipeg, MB; ^2^McMaster University, Hamilton, ON; ^3^Dalhousie University, Halifax, NS; ^4^Canadian Society of Allergy and Clinical Immunology, Ottawa, ON; ^5^University of Western Ontario, Kitchener, ON

##### **Correspondence:** Jennifer L. Protudjer

*Allergy, Asthma & Clinical Immunology* 2025, **21(Suppl 1)**:21

**Background:** In response to the need for continued professional development training in allergy, the Canadian Society of Allergy and Clinical Immunology (CSACI) introduced a food allergy educator program for physicians and allied health professionals working in allergy, in Fall 2023. In this submission, we present an update on the first two iterations of the foundational course.

**Methods**: In September 2023 and in April 2024, we launched an 8-week foundational course in food allergy, the content of which was delivered by world leaders in food allergy. This foundational course covered cover topics ranging from food allergy epidemiology, diagnosis and management, to the psychosocial burden of food allergy. At time of enrollment, participants completed a pre-test; one week following the completion of the course, participants completed a post-test (passing grade 75%). Data were analysed descriptively with comparisons made using a paired t-test (Stata Version 17.0, College Station, TX). As an educational course, the University of Manitoba Research Ethics Board deemed research ethics board approval unnecessary subsequent to review of the course proposal and overview.

**Results:** The inaugural cohort for the foundational course was Fall 2023 and included 10 learners and 9 faculty. Corresponding numbers for Spring 2024 were 12 and 8. The pre-course test mean score was 73.7% (range 50.0%-95.0%). As of April 2024, only the Spring 2023 learners have completed the post-test, for which the mean score was 86.5%, and which corresponds to a significant improvement in food allergy knowledge from the pre-test (p < 0.01).

**Conclusions:** In the first year of our needs-informed and evidence-based food allergy educator program, food allergy knowledge significantly improved amongst learners. A foundational course will be offered again in Fall 2024, and advanced practice course is also forthcoming.

## Other Allergy/Immunology

## 22 The ANTI Anticholinergic Project: targeting order sets to reduce diphenhydramine use in older adults

### Andrew Wong-Pack^1^, Rosemary Tanzini^2^, Elizabeth Logan^3^, Karim Ladha^4^, Camilla Wong^5^

#### ^1^Department of Medicine, Division of Clinical Immunology and Allergy, McMaster University., Hamilton, ON; ^2^Department of Pharmacy, St. Michael’s Hospital., Toronto, ON; ^3^Perioperative Services, Unity Health, St. Michael’s Hospital., Toronto, ON; ^4^Department of Anesthesia, Women’s College Hospital., Toronto, ON; ^5^Department of Medicine, Division of Geriatrics, St. Michael’s Hospital., Toronto, ON

##### **Correspondence:** Andrew Wong-Pack

*Allergy, Asthma & Clinical Immunology* 2025, **21(Suppl 1)**:22

**Background:** Medications with anti-cholinergic properties have many adverse effects, including sedation, delirium, cognitive impairment, and falls in the geriatric population^1−5^. When used concurrently, the risk becomes cumulative^4,6^. Diphenhydramine is listed on the Beers criteria as potentially inappropriate medication for use in older adults due to its anti-cholinergicproperties^1^. We hypothesized that by removing diphenhydramine on multiple order sets at our hospital, this would reduce the prescribing of this agent in the geriatric population (age > 65).

**Methods**: On August 30, 2022, we removed diphenhydramine from multiple electronic order sets. The order sets were revised to have alternatives for opioid-induced pruritus, including IV nalbuphine, oral cetirizine, and oral bilastine. Patients aged 65 years and older who were prescribed diphenhydramine during their admission were included.

The pre-intervention cohort included all patients meeting the above criteria between March 2022 and August 2022. The post-intervention cohort involved patients prospectively from September 2022 to February 2023. Standardized chart review identified the total number of doses of diphenhydramine received stratified by age, gender, and route of administration. The study was deemed not to require REB approval given it was a QI initiative through ReQUIST.

**Results:** Pre-intervention there were 569 total diphenhydramine doses in adults aged > 65 (N = 569). 182 doses were in males (75 PO, 107 IV), and 387 in females (172 PO 215 IV). Post-intervention there were 474 total diphenhydramine doses in adults aged > 65 (N = 474). 215 doses were in males (74 PO, 141 IV), and 259 in females (123PO, 136 IV). Overall, there was an 18.2% (N = 95) reduction in total diphenhydramine doses.

**Conclusions:** Our study is the first QI initiative explicitly looking at reducing inpatient prescriptions of diphenhydramine in older adults. However, numerous variables contribute to diphenhydramine use beyond our targeted order sets. Ultimately, further initiatives are needed to facilitate culture change in the widespread use of diphenhydramine for medical inpatients.

**References:**

1. Fick, D. M., Semla, T. P., Steinman, M., Beizer, J., Brandt, N., Dombrowski, R., DuBeau, C. E., Pezzullo, L., Epplin, J. J., Flanagan, N., Morden, E., Hanlon, J., Hollmann, P., Laird, R., Linnebur, S., & Sandhu, S. (2019). American Geriatrics Society 2019 Updated AGS Beers Criteria® for Potentially Inappropriate Medication Use in Older Adults. *Journal of the American Geriatrics Society*, *67*(4), 674–694. 10.1111/jgs.15767
2. Collamati, A., Martone, A. M., Poscia, A., Brandi, V., Celi, M., Marzetti, E., Cherubini, A., & Landi, F. (2016). Anticholinergic drugs and negative outcomes in the older population: from biological plausibility to clinical evidence. *Aging Clinical and Experimental Research*, *28*(1), 25–35.
3. 10.1007/s40520-015-0359-7
4. Agostini, J. V., Leo-Summers, L. S., & Inouye, S. K. (2001). Cognitive and other adverse effects of diphenhydramine use in hospitalized older patients. *Archives of Internal Medicine*, *161*(17), 2091–2097. 10.1001/archinte.161.17.2091
5. Hilmer, S. N., Mager, D. E., Simonsick, E. M., Ling, S. M., Windham, B. G., Harris, T. B., Shorr, R. I., Bauer, D. C., & Abernethy, D. R. (2009). Drug Burden Index Score and Functional Decline in Older People. *American Journal of Medicine*, *122*(12), 1142–1149. 10.1016/j.amjmed.2009.02.021
6. Simons, F. E. R., Fraser, T. G., Maher, J., Pillay, N., & Simons, K. J. (1999). Central nervous system effects of H1-receptor antagonists in the elderly. *Annals of Allergy, Asthma and Immunology*, *82*(2), 157–160. 10.1016/S1081-1206(10)62590-2

## 23 FPIES in a Canadian center: clinical presentation, management, and resolution

### Angela Mulé^1^, Adnan Al Ali^1^, Vicky Le Blanc^2^, Christine McCusker^1^, Pasquale Mulé^1^, Catherine Prattico^1^, Xun Zhang^1^, Moshe Ben-Shoshan^1^

#### ^1^Montreal Children’s Hospital, McGill University Health Centre, Montreal, QC; ^2^BC Children’s Hospital, University of British Colombia, Vancouver, BC

##### **Correspondence:** Angela Mulé

*Allergy, Asthma & Clinical Immunology* 2025, **21(Suppl 1)**:23

**Background:** Data on food protein induced enterocolitis syndrome (FPIES) are sparse. We aimed to evaluate sociodemographic characteristics, co-morbidities, and triggers of children presenting with FPIES. Tolerance to baked goods and resolution a year following diagnosis were also assessed.

**Methods**: Children with physician-diagnosed FPIES were enrolled and followed at the Montreal Children’s Hospital and an affiliated clinic. Families were queried on the food trigger and co-morbidities, as well as clinical characteristics of reaction and management. A severe reaction was defined as vomiting 4 or more times, altered behaviour/lethargy, pallor, dehydration, and need for IV fluids [1].

**Results:** Between November 2021 and June 2024, 66 children with a confirmed history of FPIES were enrolled. Patient ages ranged from 1 to 156 months old (median 7 months), and 45% were males. Food triggers included egg (32%), milk (20%), shellfish (20%), oat (15%), fish (12%), soy/grains (9%), fruit (8%), peanut (8%), and rice (2%). Six patients (9%) had 2 or more food triggers. Co-morbidities included eczema (31 patients, 46%), asthma (11 patients, 16%), and other allergens (15 patients, 22%). The most common symptoms were vomiting (92%), lethargy (26%), pallor (24%), diarrhea (15%), and dehydration (11%). Symptoms developed in the majority of cases within 1–4 h of exposure. Seven patients (18%) had a severe reaction. Among 22 patients who tried baked goods (milk, egg, oat, soy/grains), 15 (68%) were tolerant. Amongst 49 patients who were followed, 32 patients (65%) ingested the culprit allergen and 6 patients (19%) had a reaction within a year subsequent to their last visit.

**Conclusions:** The most common food triggers are egg, milk, and shellfish. The majority of reactions are not severe and baked goods containing the culprit food are often tolerated. Resolution of FPIES was reported in most patients one year following diagnosis.

**References:**

Nowak-Węgrzyn A, Chehade M, Groetch ME, et al. International consensus guidelines for the diagnosis and management of food protein-induced enterocolitis syndrome: Executive summary-Workgroup Report of the Adverse Reactions to Foods Committee, American Academy of Allergy, Asthma & Immunology. J Allergy Clin Immunol. 2017;139(4):1111–1126.e4. doi: 10.1016/j.jaci.2016.12.966

## 24 Early experiences from connexion nordique: education for local capacity building in northern Quebec

### Clara Long^1^, Michael Aw^1^, Adnan Al Ali^2^, Megan Park^3^, Maryam El Alaoui^4^, Michelle Kwok^2^, Michael Fein^2^

#### ^1^McGill University Division of Internal Medicine, Montreal, QC; ^2^McGill University Division of Allergy and Immunology, Montreal, QC; ^3^University of Toronto Department of Medicine, Toronto, ON; ^4^McGill University School of Population and Global Health, Montreal, QC

##### **Correspondence:** Clara Long

*Allergy, Asthma & Clinical Immunology* 2025, **21(Suppl 1)**:24

**Background:** Indigenous communities in Canada face a higher burden of atopic diseases, with access to care being the primary barrier to effective diagnosis and treatment [1, 2]. In Quebec’s Indigenous health regions—Nunavik and Cree Territory of James Bay—access to allergy care is limited, resulting in long wait times, cultural disconnection, and missed appointments. Nunavik has two hospitals serving fourteen communities, while James Bay has one hospital serving nine communities. Connexion Nordique, a quality improvement project by the McGill University Health Centre, aims to enhance allergy care accessibility for Quebec’s Indigenous populations. The education arm focuses on community partnerships to build local capacity. We present the initial experiences of this educational initiative.

**Methods**: Connexion Nordique began with site visits to build relationships with healthcare providers, community leaders, and patients. Initial needs were assessed through surveys and preliminary interviews with doctors and nurses. Based on these assessments, reference materials addressing common allergy presentations were created and supplemented with live educational sessions. This study uses mixed methodology including survey, focus groups, and resource utilization metrics.

**Results:** Site visits in five communities were conducted from January 2023-February 2024. Protocols for anaphylaxis and penicillin allergy were locally adapted and distributed throughout communities. In April 2024, the first live educational seminar on chronic urticaria was held, with 16 Nunavik primary care physicians in attendance. A focus group revealed positive reception, with feedback that severe/refractory cases are not suitable for local management.

**Conclusions:** Rural and Indigenous health disparities are well documented, with ongoing knowledge gaps in allergic disorders within the Canadian context [3]. The project will expand educational sessions and resources and explore telemedicine for enhanced specialist access. Future steps include implementing workshops and practicums for drug allergy delabeling to empower local physicians, thus linking education to outcomes. Ongoing data collection will guide program refinements.

**References:**

1. Jafri S, Janzen J, Kim R, Abrams EM, Gruber J, Protudjer JLP. Burden of Allergic Disease in Racial and Ethnic Structurally Oppressed Communities Within Canada and the United States: A Scoping Review. The Journal of Allergy and Clinical Immunology: In Practice. 2022 Nov 1;10(11):2995–3001.
2. Harrington DW, Wilson K, Elliott SJ, Clarke AE. Diagnosis and treatment of food allergies in off-reserve Aboriginal children in Canada. Canadian Geographies / Géographies canadiennes. 2013;57(4):431–40.
3. Pongdee T, Brunner WM, Kanuga MJ, Sussman JH, Wi CI, Juhn YJ. Rural Health Disparities in Allergy, Asthma, and Immunologic Diseases: The Current State and Future Direction for Clinical Care and Research. J Allergy Clin Immunol Pract. 2024 Feb;12(2):334–44.

## 25 Gliadin in hen’s egg and cow’s milk

### Kevin K. Zhang^1^, Peter Vadas^2^

#### ^1^Temerty Faculty of Medicine, Toronto, ON; ^2^Division of Clinical Immunology and Allergy, St. Michael’s Hospital, Toronto, ON

##### **Correspondence:** Kevin K. Zhang

*Allergy, Asthma & Clinical Immunology* 2025, **21(Suppl 1)**:25

**Background:** Intact gliadin has been detected in human breast milk. We postulated that non-degraded gliadin may also be present in animal products, such as cow’s milk and hen’s eggs. If present in these foods, gliadin in milk and eggs could represent an unrecognized source of gluten exposure in patients with celiac disease (CD) refractory to a strict gluten-free diet.

**Methods**: Gliadin was quantified using the Neogen Veratox ELISA. Egg white and yolk fractions were sampled separately. Both white and brown eggs, laid by White Leghorn and Rhode Island Red hens, were sourced from 10 different providers, including hens that were free-roaming and hens raised in cages. Eggs were considered “gluten-free” if they contained 10 ppm gliadin or less. Cow’s milk was sampled from 8 different providers. The limit of detection of this assay was 2.5–40 ppm.

**Results:** Gliadin was detected in 26 of 131 eggs tested (19.8%), with no difference between the white and yolk fractions (p = 0.562). There was also no difference in gliadin content between brown and white eggs (p = 0.437). Gliadin was present in only 2 of the 69 (2.9%) samples from caged hens, compared to 28 of 190 (14.7%) samples from free-roaming hens (p = 0.008). Notably, 11 of 131 eggs (8.4%) contained > 10 ppm gliadin, with 6 eggs containing gliadin at concentrations over 40 ppm. Gliadin was not detectable in any samples of cow’s milk.

**Conclusions:** Significant concentrations of intact gliadin were detected in hen’s eggs. Assuming a gliadin concentration of 40 ppm or higher, two large eggs could contain over 8 mg of gluten. The safe daily gliadin intake for patients with refractory CD is not known but may be considerably less than patients with diet-responsive CD. Consequently, hen’s eggs may represent a previously unrecognized source of gliadin exposure in individuals with CD.

## 26 Gene alterations in inborn errors of immunity and their impact on pediatric cancers: implications for oncogenesis, progression, and outcomes

### Samuel Lokanc^1^, Patrick Sipila^1^, Ritul Sharma^1^, Geoff Cuvelier^2^, Luis Murguia-Favela^2^, Nicola Wright^2^, Eyal Grunebaum^3^, Aru Narendran^1,2^

#### ^1^University of Calgary, Calgary, AB; ^2^Alberta Children’s Hospital, Calgary, AB; ^3^Hospital for Sick Children, Toronto, ON

##### **Correspondence:** Samuel Lokanc, Patrick Sipila

*Allergy, Asthma & Clinical Immunology* 2025, **21(1)**:28

**Background:** Inborn errors of immunity (IEI) are genetic disorders impairing the immune system, leading to increased susceptibility to infections, autoimmunity, inflammation, and malignancies. Over 500 specific gene mutations have been associated with various IEIs, suggesting a complex genetic landscape for cancer susceptibility. This study aims to investigate IEI-related gene alterations and differential gene expression in the somatic transcriptomes of cancers and their potential contributions to tumor development, progression, and survival.

**Methods**: RNA-sequencing data from adult and pediatric cancers were accessed from the TCGA and TARGET databases, using the ‘TCGAbiolinks’ package in R alongside normal tissue data from GTEx via the’recount2’ package. Samples were filtered and preprocessed for relevance and quality. Differential expression analysis (DEA) of 592 IEI-related genes was conducted using the’limma-voom’ pipeline. Gene ontology (GO) analysis identified overrepresented and underrepresented functions in the cancer population.

**Results:** Of the 592 IEI-related genes, 50.2% (297/592) were significantly downregulated, while 38.9% (232/592) were significantly upregulated across all cancer types. The top overrepresented GO functions included humoral immune response and complement activation, while the top underrepresented GO functions included DNA damage response and DNA repair. Complement system-related genes were notably dominant, with 7 of the top 25 downregulated genes and 8 of the top 25 upregulated genes being complement components. These findings suggest that complement system activation may be advantageous to tumors, aligning with literature indicating that sublytic complement activation can support tumor growth by inhibiting TNF-mediated apoptosis, which evidently were among the most downregulated genes across all cancer types (TNFRSF9, TNFSF11, TNFRSF11A, TNFAIP3).

**Conclusions:** This study presents a bioinformatic analysis of somatic transcriptomes in cancer, identifying distinct IEI-associated alterations and their potential contribution to tumor growth and progression. The findings provide important initial information for future investigations to advance the care of a key group of cancer patients.

## 27 Knowledge assessment tools in atopic dermatitis patient education: a scoping review

### Jasmin Khela, Bethany Wilken, Yuka Asai

#### Queen’s University, Kingston, ON

##### **Correspondence:** Jasmin Khela

*Allergy, Asthma & Clinical Immunology* 2025, **21(Suppl 1)**:27

**Background:** Atopic dermatitis (AD) is a chronic and inflammatory skin disease which requires continuous self-management by patients/caregivers. Patient education in AD improves clinical outcomes, treatment adherence, and self-management practices. Patient-reported outcome measures have been used to assess the effectiveness of AD patient education interventions, however they have limited use in assessing learning outcomes, such as knowledge. The literature on knowledge outcome measures for AD patient education interventions has not been examined to date.

**Methods**: We performed a scoping review of the literature on knowledge assessment tools for AD patient education interventions following the PRISMA-ScR framework. Search databases included MEDLINE, Embase, CINAHL, Education Source, Web of Science, Grey Matters, Clinical Trials.gov, and the International Clinical Trials Registry Platform. Of the 3914 articles identified from the search strategy, 20 studies were eligible for data extraction.

**Results:** Most studies were randomised controlled trials originating in the United States, Europe, and/or Asia, and published in the years of 2003–2023. Studies commonly assessed caregivers’ knowledge of AD and included assessments of clinical outcome measures. Similar methods were employed for assessing subjective knowledge across studies. Likewise, studies assessing AD patients’/caregivers’ objective knowledge used comparable methods. Multiple-choice and true/false question formats were used in objective knowledge assessments, and Likert-type scales were common for assessing subjective knowledge. Objective knowledge assessments consisted of more questions than subjective knowledge outcome measures. Content assessed in knowledge outcome measures was relatively consistent across studies. Delivery of subjective and objective AD knowledge assessments was by telephone, in clinic, and/or online. In pre- and post-test study designs, identical knowledge outcome measures were administered.

**Conclusions:** This scoping review highlights the diverse components of knowledge assessment tools for AD patient education interventions. Further studies on developing and validating high-quality AD knowledge outcome measures are needed for assessing the true effects of patient education interventions on improving patients’ knowledge.

## 28 Enhancing pediatric trainee knowledge and comfort with key allergy and immunology topics

### Ashna Asim, Vy Kim

#### University of Toronto, Toronto, ON

##### **Correspondence:** Ashna Asim

*Allergy, Asthma & Clinical Immunology* 2025, **21(Suppl 1)**:28

**Background:** Clinical immunology and allergy (CIA) topics are included in the Royal College Objectives for Pediatric training. This project aimed to better understand educational needs, relating to CIA topics, of pediatric trainees during their CIA rotation at the University of Toronto.

**Methods**: Review of the CIA curriculum was done through the lens of Kern’s Framework for Curriculum Development. In Phase I of this project, a needs assessment was done of pediatric residents (n = 28).

Study data were collected and managed using Research Electronic Data Capture (REDCap) hosted at The Hospital for Sick Children. The survey consisted of eight questions including: close-ended questions, Likert scale questions, and open-ended questions. The survey was distributed from July to November 2022 and results were analyzed.

For Phase II of this project, a plan-do-study-act (PDSA) framework was applied. An 11-item knowledge-based REDCap survey was developed to assess trainee competence pre/post-CIA rotation to measure learning outcomes prior to and after e-module implementation.

**Results:** In the Phase I survey, 75% of residents felt uncomfortable diagnosing inborn errors of immunity (IEI) and only 35.7% of pediatric residents felt comfortable in an approach to recurrent infections. Approach to recurrent infections and IEI were selected as the top 2 high-yield topics (92.9% and 78.6% respectively). Most residents (82%) favoured hand-outs and didactic teaching, with case-based e-modules selected by over half of residents. As didactic teaching is already incorporated, a case-based clinical immunology e-module was developed. Phase II knowledge-based survey data collection is in process.

**Conclusions:** This project identified the need to optimize teaching of CIA topics to pediatric trainees. This project can therefore provide a blueprint for enhancing CIA curriculum for pediatric trainees at other academic institutions. By applying a PDSA model, this project demonstrates the importance of re-assessment and re-adjustments of curriculum to optimize learner needs.

**References:**

PA Harris, R Taylor, R Thielke, J Payne, N Gonzalez, JG. Conde, Research electronic data capture (REDCap) – A metadata-driven methodology and workflow process for providing translational research informatics support, J Biomed Inform. 2009 Apr;42(2):377–81. 

## 29 Enhancing accessibility and cultural safety in community allergy and immunology care—identifying patient reported barriers

### Brittany Curry^1^, Sean Duke^1^, Rosemary Invik^2^, Zaneta Lim^3^, Samira Jeimy^4^, Scott B. Cameron^2,5^, Victoria E. Cook^2,5^

#### ^1^Department of Pediatrics, University of British Columbia, Vancouver, BC; ^2^Community Allergy Clinic, Island Health Authority, Victoria, BC; ^3^Community Pediatrics Clinic, Island Health Authority, Nanaimo, BC; ^4^Division of Clinical Allergy and Immunology, Western University, London, ON; ^5^Division of Allergy, Department of Pediatrics, University of British Columbia, Vancouver, BC

##### **Correspondence:** Brittany Curry

*Allergy, Asthma & Clinical Immunology* 2025, **21(Suppl 1)**:29

**Background:** Allergic disorders are highly prevalent in Canada, resulting in long wait times to receive allergy and immunology care. This situation can be especially challenging for patients who face additional barriers to healthcare access, including rural and remote populations, low socioeconomic status, poor health literacy, and racism and discrimination [1,2]. Inequitable access to care results in worsened health outcomes. Existing literature addresses interventions to broadly reduce disparities in allergy care; however, few publications identify practical interventions that community providers can employ to ensure delivery of culturally safe and accessible care within culturally and geographically diverse populations [3–6]. We are applying quality improvement (QI) methodology to improve accessibility and cultural safety of community allergy and immunology care in Northern and Island Health regions of British Columbia.

**Methods**: Given the lack of literature describing barriers and interventions in our specific context, we began with a current state analysis to improve our understanding of primary and secondary drivers impacting accessibility and cultural safety. Beginning in January 2023, patients referred to our community allergy practice were invited to answer a question regarding perceived barriers to care. Deidentified free text responses will undergo thematic analysis to categorize barriers. Additionally, we will perform semi-structured interviews from July to September 2024 with patients who have received hybrid virtual/in person allergy/immunology care in the Northern Health region to gain a deeper understanding of their experience accessing care. Deidentified interview transcripts will undergo thematic analysis.

**Results:** Barriers to care identified in our preliminary analysis of the written patient responses included lack of transportation, language barrier, rural and remote location, and a history of racial discrimination. Semi-structured interviews are in progress.

**Conclusions:** Next steps involve identifying potential interventions that can be evaluated in plan-do-study-act (PDSA) cycles, facilitating continuous improvement of accessibility and cultural safety in community allergy and immunology care.

**References:**

1. Warren CM, Turner PJ, Chinthrajah RS, Gupta RS. Advancing food allergy through epidemiology: understanding and addressing disparities in food allergy management and outcomes. J Allergy Clin Immunol Pract. 2021;9(1):110–118.
2. Fenton N, Elliott S, Vine M, Hampson C, Latycheva O, Barker K, et al. **Assessing needs: asthma in first nations and inuit communities in Canada**. Pimatisiwin A J Aborig Indig Community Heal. 2012;**10**(1)71-77.
3. Ogbogu PU, Capers Q, Apter AJ. **Disparities in asthma and allergy care: what can we do?** J Allergy Clin Immunol Pract. 2021;**9**(2):663–669.
4. Udemgba C, Sarkaria SK, Gleeson P, Bryant-Stephens T, Ogbogu PU, Khoury P, et al. **New considerations of health disparities within allergy and immunology.** J Allergy Clin Immunol. 2023;**151**(2):314–23.
5. Pappalardo AA, Codispoti CD, Mahdavinia M. **Health care access in allergy and immunology: problems and potential solutions.** J Allergy Clin Immunol. 2023;**153**(2):401–403.
6. Ogbogu PU, Noroski LM, Arcoleo K, Reese BD, Apter AJ. **Methods for cross-cultural communication in clinic encounters.** J Allergy Clin Immunol Pract. 2022;**10**(4):893–900.

## 30 Inappropriate penicillin allergy delabeling: an LHSC CTU quality improvement initiative

### Brianna Barsanti-Innes, Sherjeel Anwar, Erin Spicer, Samira Jeimy

#### Western University, London, ON

##### **Correspondence:** Brianna Barsanti-Innes

*Allergy, Asthma & Clinical Immunology* 2025, **21(Suppl 1)**:30

**Background:** Penicillin allergy delabeling in other centers in Canada has been shown to be safe and effective through strategies including thorough patient histories and validated point of care scores, direct oral challenges, skin testing, and/or referral to Allergy and Immunology. Inpatient admission under internal medicine at a tertiary care hospital offers an opportunity to implement these strategies within a quality improvement framework, with the aim to improve both immediate patient treatment and downstream individual and health system outcomes.

**Methods**: Our aim is to reduce the inappropriate penicillin allergy documentation for patients admitted to Internal Medicine Clinical Teaching Units at University Hospital, London Health Sciences Centre by 80% by June 2025. We will identify patients admitted to CTU between September 2024 and June 2025, who have a label of penicillin allergy on the hospital EMR. We will then utilize the PEN-FAST score to stratify patients into low and moderate/high risk for penicillin allergies. Patients with a low score will undergo direct oral challenges prior to delabeling. Those with higher PEN-FAST scores will be referred to the division of Allergy for further assessment. Patient education tools and standardized family physician and outpatient pharmacy communication will be implemented to minimize the risk of relabeling.

**Results:** Baseline data review of patients admitted to CTUs at University Hospital from May 2022 until May 2023 revealed that 13% (473/3661 patients) had EMR documentation of a reaction to a penicillin antibiotic, with 92% of these reactions being described as “unknown”. We anticipate that a significant proportion of these patients will be appropriate for delabeling through our quality improvement initiative.

**Conclusions:** Through this quality improvement initiative we anticipate delabeling a significant proportion of inappropriate penicillin allergies thereby increasing the appropriate use of penicillin/beta-lactam antibiotics to improve both individual and system healthcare delivery at our center.

**References:**

1. Trubiano JA, Vogrin S, Chua KYL, et al. Development and Validation of a Penicillin Allergy Clinical Decision Rule. JAMA Intern Med. 2020;180(5):745–752. doi:10.1001/jamainternmed.2020.0403
2. Jeimy S, Ben-Shoshan M, Abrams EM, Ellis AK, Connors L, Wong T. Practical guide for evaluation and management of beta-lactam allergy: position statement from the Canadian Society of Allergy and Clinical Immunology. Allergy Asthma Clin Immunol. 2020;16(1):95. Published 2020 Nov 10. doi:10.1186/s13223-020-00494-2

## 31 Evaluating oral antihistamine prescriptions for older adults during hospitalization

### Joella Ho^1^, Kylie McNeill^1^, Marcel Miron-Celis^1,2^, Derek Dyks^3^, Shirley Huang^1,2,4^

#### ^1^Department of Medicine, University of Ottawa, Ottawa, ON; ^2^Ottawa Hospital Research Institute, Ottawa, ON; ^3^Department of Pharmacy, The Ottawa Hospital, Ottawa, ON; ^4^Division of Geriatric Medicine, The Ottawa Hospital, Ottawa, ON

##### **Correspondence:** Joella Ho

*Allergy, Asthma & Clinical Immunology* 2025, **21(Suppl 1)**:31

**Background:** First-generation antihistamines, such as diphenhydramine, have increased negative effects—including delirium in older adults, drowsiness, and anticholinergic burden—compared with newer generation antihistamines, such as loratadine [1–3]. The 2023 American Geriatric Society Beers Criteria® [4] and a 2019 Canadian Society of Allergy and Immunology position statement [5] recommend avoiding diphenhydramine and preferentially using newer generation antihistamines, respectively. With this project, we sought to evaluate if antihistamines are being prescribed appropriately for older patients admitted to a large academic hospital and if not, to determine where we can improve prescriber awareness and compliance with the Beers Criteria® and CSACI recommendations.

**Methods**: We identified and queried the cohort of interest, defined as patients over the age of 70 who were prescribed oral antihistamines during admission to non-surgical services between April 1 and June 30, 2023, using MDClone, a source of validated health administrative data at our hospital. A retrospective chart review was then used to verify details including the admitting service, administered doses of antihistamines, and the indication for antihistamine prescription.

**Results:** Diphenhydramine was prescribed more frequently than loratadine (91 versus 72 prescriptions). The two most common indications for prescribing diphenhydramine were pruritus (34%) and other unspecified reasons (40%), while the two most common indications for prescribing loratadine were pruritus (30%) and allergic rhinitis (33%). Of 44 prescriptions for treating urticaria or allergic rhinitis, 31 (70%) appropriately used loratadine as per the CSACI position statement. Admitting services with the most room for improvement were Family Medicine, Neurology, Hospitalist, and Medicine.

**Conclusions:** This study highlights the need for improved provider awareness of indications for antihistamines, risks of older generation antihistamines, and the availability of newer generation antihistamines. Using a quality improvement framework, we developed several change ideas to improve inpatient prescribing practices among medical practitioners at our institution.

**References:**

1. Agostini JV, Leo-Summers LS, Inouye SK. Cognitive and other adverse effects of diphenhydramine use in hospitalized older patients. *Arch Intern Med*. 2001;**161**:2091–2097.
2. Ramos H, Moreno L, Pérez-Tur J, Cháfer-Pericás C, García-Lluch G, Pardo J. CRIDECO anticholinergic load scale: An updated anticholinergic burden scale. Comparison with the ACB scale in Spanish individuals with subjective memory complaints. *J Pers Med*. 2022;**12**:207.
3. Simons FE, Simons KJ. Histamine and H1-antihistamines: celebrating a century of progress. *J Allergy Clin Immunol*. 2011;**128**:1139–1150.e4.
4. 2023 American Geriatrics Society Beers Criteria® Update Expert Panel. American Geriatrics Society 2023 updated AGS Beers Criteria® for potentially inappropriate medication use in older adults. *J Am Geriatr Soc*. 2023;**71**:2052–2081.
5. Fein MN, Fischer DA, O’Keefe AW, Sussman GL. CSACI position statement: Newer generation H_1_-antihistamines are safer than first-generation H_1_-antihistamines and should be the first-line antihistamines for the treatment of allergic rhinitis and urticaria. *Allergy Asthma Clin Immunol*. 2019;**15**:61.

## 32 Effectiveness of a provincial policy to improve epinephrine use for anaphylaxis in schools in Alberta, Canada: a pre-post study

### Joseph Najem^1^, Alexandre Ton That^1^, Connor Prosty^1^, Ann E. Clark^9^, Judy Morris^2^, Jocelyn Gravel^3^, Rodrick Lim^4^, Edmond S. Chan^5^, Ran D. Goldman^5^, Andrew O’Keefe^6^, Jennifer Gerdts^10^, Derek K. Chu^7^, Julia Upton^8^, Elana Hochstadter^8^, Jocelyn Moisan^11^, Adam Bretholz^12^, Christine McCusker^13^, Xun Zhang^15^, Jennifer L. Protudjer^14^, Elissa M. Abrams^16^, Elinor Simons^16^, Andrew Dixon^17^, Moshe Ben-Shoshan^18^

#### ^1^McGill University, Montreal, QC; ^2^Sacré-Coeur Hôpital, Montreal, QC; ^3^Centre Hospitalier Universitaire Sainte-Justine, Montreal, QC; ^4^Children’s Hospital at London Health Science Centre, London, ON; ^5^British Columbia Children’s Hospital, Vancouver, BC; ^6^Memorial University, St. John’s, NL; ^7^McMaster University, Hamilton, ON; ^8^University of Toronto, Toronto, ON; ^9^Cumming School of Medicine, Calgary, AB; ^10^Food Allergy Canada, Toronto, ON; ^11^Emergency Medical Services of Outaouais, Outaouais, QC; ^12^Department of Pediatrics, Montreal Children’s Hospital, Montreal, QC; ^13^Division of Allergy and Clinical Immunology, Department of Pediatrics, Montreal Children’s Hospital, Montreal, QC; ^14^Department of Pediatrics and Child Health, Children’s Hospital Research Institute of Manitoba, Winnipeg, MB; ^15^Centre for Outcomes Research and Evaluation, Research Institute of McGill University Health Centre, Montreal, QC; ^16^Department of Pediatrics and Child Health, Section of Allergy and Clinical Immunology, Children’s Hospital Research Institute of Manitoba, Winnipeg, MB; ^17^Department of Pediatrics, University of Alberta, Edmonton, AB; ^18^Division of Allergy and Clinical Immunology, Department of Pediatrics, Montreal Children’s Hospital, McGill University Health Centre, Montreal, QC

##### **Correspondence:** Joseph Najem

*Allergy, Asthma & Clinical Immunology* 2025, **21(Suppl 1)**:32

**Background:** Protection of Students with Life-Threatening Allergies Act, enacted in 2020, obligates every school board across Alberta, Canada to hold a common use stock of at least one epinephrine autoinjector (EAI) and to maintain a protocol for the prevention and management of anaphylaxis. The effectiveness of this policy remains unclear. Herein, we aimed to evaluate whether pre-hospital EAI use increased following the implementation of the Act.

**Methods**: As part of the Cross-Canada Anaphylaxis (C-CARE), which includes a site in Edmonton, Alberta. Pediatric cases of anaphylaxis, defined as the involvement of ≥ 2 systems and/or hypotension, that occurred at school and presented to the Stollery Children’s Hospital’s Emergency Department in Edmonton, Alberta from 2016–2023 were retrospectively identified. Data were collected on demographics, comorbidities, symptomatology, pre- and intra-hospital management, and outcome of anaphylaxis by standardized chart review. Multivariable logistic regression was used to assess pre-hospital anaphylaxis management before versus after the enactment of the Act on January 1, 2020. Adjusted for age and previously known food allergy and reported as adjusted odds ratios (aOR) and 95 percent confidence intervals (95%CI). This study was approved by the McGill University Research Ethics Board.

**Results:** Of the 44 cases of anaphylaxis in school identified over the study period, 54.5% occurred post-Act. The median age was 12.9 (interquartile range = 9.5–15.5) and 56.8% were female. There was no difference in pre-hospital antihistamine use pre versus post Act (aOR = 0.98, 95%CI = 0.3–3.3). However, there was greater post-act use of pre-hospital EAI in schools versus pre-act (60.0% versus 30.0%, aOR = 3.9, 95%CI 1.1–13.9). No reactions resulted in hospital admittance.

**Conclusions:** Implementation of the Act was associated with an increase in pre-hospital EAI use in cases of pediatric anaphylaxis occurring at school. These preliminary results support the effectiveness of policy interventions for anaphylaxis management.

## 33 Evaluating the performance of large language models in clinical immunology and allergy: a pilot study

### Joshua V. Yu^1^, Rongbo Zhu^2^

#### ^1^Department of Medicine, Faculty of Medicine, McMaster University, Hamilton, ON; ^2^Division of Clinical Immunology and Allergy, Department of Medicine, Western University, London, ON

##### **Correspondence:** Joshua V. Yu

*Allergy, Asthma & Clinical Immunology* 2025, **21(Suppl 1)**:33

**Background:** Large language models (LLMs) have gained significant popularity and widespread use in recent years. Despite their potential, the accuracy of readily accessible LLMs in answering questions specific to Clinical Allergy and Immunology (CIA) has not been formally validated. This study aims to evaluate the performance of LLMs in this domain, marking a preliminary step toward broader medical validation in the field of CIA.

**Methods**: We used 299 publicly available questions from the American College of Allergy, Asthma, and Immunology (2018–2023) and 49 questions from an internationally standardized question bank. Two LLMs, GPT-3.5-turbo and GPT-4o, were tested using both a zero-shot approach and multi-agent retrieval-augmented generation (RAG), a technique that allows LLMs to access curated contextual information before responding. For RAG, resources included PDFs of publicly available society guidelines and a comprehensive allergy-immunology textbook. Text embeddings were generated with the open-source *mixedbread-ai/mxbai-embed-large-v1* model, and context retrieval queries were generated using GPT-4o. We calculated the proportion of correct answers for each approach and analyzed differences using Cochran’s Q test and post-hoc McNemar’s tests, corrected for multiple comparisons.

**Results:** Zero-shot GPT-3.5 scored 60%, significantly lower than GPT-4o, which scored 80% (p < 0.001). GPT-3.5 with RAG on all available sources scored 74%, significantly better than zero-shot GPT-3.5 (p < 0.001); however, it scored significantly worse than zero-shot GPT-4o (p = 0.04). GPT-4o with RAG scored 82%, which was not significantly different than zero-shot GPT-4o.

**Conclusions:** Both GPT-4o and GPT-3.5 augmented with RAG demonstrate proficiency in answering allergy-immunology questions. Future research should include subgroup analysis of model performance by question type and exploration of variations in RAG implementation and prompting. Comparing these models to human performance and evaluating more LLMs will further quantify their accuracy.

## 34 No increased risk of atopic conditions with parent-reported childhood immunizations in the CHILD cohort study

### Keely Loewen^1,2^, Scarlet Deluz^3^, Thomas Rawliuk^3^, Theo J. Moraes^4^, Stuart Turvey^5^, Piushkumar Mandhane^6^, Meghan B. Azad^2,3^, Padmaja Subbarao^4^, Elinor Simons^1,2,3^

#### ^1^Section of Allergy & Clinical Immunology, Department of Pediatrics & Child Health, University of Manitoba, Winnipeg, MB; ^2^Department of Pediatrics & Child Health, University of Manitoba, Winnipeg, MB; ^3^Children’s Hospital Research Institute of Manitoba, Winnipeg, MB; ^4^Hospital for Sick Children and University of Toronto, Toronto, ON; ^5^University of British Columbia, Vancouver, BC; ^6^University of Alberta, Edmonton, AB

##### **Correspondence:** Keely Loewen

*Allergy, Asthma & Clinical Immunology* 2025, **21(Suppl 1)**:34

**Background:** Childhood immunization plays an important role in prevention of communicable infections. Many studies and meta-analyses have found no association between vaccines and atopic conditions. We explored possible association between routine childhood immunization and atopic conditions in the CHILD Cohort Study.

**Methods**: CHILD recruited expecting parents delivering infants in 2009–2012. We included children with completed questionnaires and clinical assessments including history, physical examination, and skin prick testing to food and aeroallergens at ages 1, 3, and 5 years. Completeness of routine immunizations was determined by parental report. Atopic dermatitis, asthma, allergic rhinitis, and clinical food allergy were diagnosed by study physicians. We used multivariable logistic regression to compare associations between immunizations in infancy and early childhood, and diagnoses of these allergic conditions, adjusting for study centre, sex, exclusive breastfeeding at 6 months, and prenatal smoke exposure.

**Results:** Complete routine immunization was reported by parents for 2332 infants (71.5%) receiving 2-, 4-, and 6-month immunizations, 2029 children (62.2%) receiving 1-year immunizations, and 835 children (25.6%) receiving school-age immunizations by age 5 years. The remaining participants were considered to have unconfirmed, delayed, or incomplete immunization.

There was no increased risk of moderate-severe atopic dermatitis (msAD), wheeze, or sensitization at age 1 year with complete immunization up to and including age 6 months. There was no increased risk of msAD (OR 0.51, 95% CI 0.35–0.75), wheeze (OR 1.39, 95% CI 0.82–2.37), sensitization (OR 0.94, 95% CI 0.74–1.18), asthma (OR 0.63, 95% CI 0.46–0.87), allergic rhinitis (OR 0.64, 95% CI 0.39–1.07), or food allergy (OR 0.85, 95% CI 0.58–1.26) at age 3 years with complete immunizations up to and including age 1 year, or at age 5 years with complete immunizations up to and including school age.

**Conclusions:** CHILD participants did not have an increased risk of atopic conditions with parent-reported complete routine childhood immunizations.

## 35 Characterization of pollen rupture in an allergen exposure chamber

### Nicholas Ogrodnik, Laura Haya, Owen Duncan, Ghena El Tayech, Andrew Comiskey, Suzanne Kelly, Rachel Friedrich, Alissa Belanger, Jimmy Yang, William Yang

#### Red Maple Trials, Ottawa, ON

##### **Correspondence:** Nicholas Ogrodnik

*Allergy, Asthma & Clinical Immunology* 2025, **21(Suppl 1)**:35

**Background:** Thunderstorm asthma (TA) is known to induce asthmatic reactions in large populations following thunderstorms due to the inhalation of ruptured pollen grains, leading to sudden surges in acute respiratory illnesses which burden local health-care providers [1]. With the evolution of the global climate crisis, projections show increased aeroallergen concentrations and frequency of thunderstorms [2]. As such, TA outbreaks are becoming an increasing concern. This study attempts to characterize ruptured pollen in our Allergen Exposure Chamber (AEC) to: (1) Ensure allergen exposures in our AEC remain naturalistic; and (2) Understand the causes of pollen rupture which can lead to TA.

**Methods**: Timothy grass pollen integrity was studied during controlled aerosolization in our AEC at a relative humidity of 40–55%. The source of rupturing and rupture mechanism were investigated by aerosolizing pollen through our custom dispersion system and collecting samples at endpoints throughout the AEC. Samples were stained, imaged and processed using custom counting and characterization algorithms to determine rupture percentage and characterize rupture features.

**Results:** Pollen aerosolized in the AEC had no significant rupture. Following staining of samples collected on slides placed on the AEC floor, approximately 12% of pollen ruptured due to osmotic pressure. Also, rotational impact samplers caused additional mechanical rupture, showing rupture levels of approximately 18%. Furthermore, our custom counting algorithm was validated, showing an average percent error of less than 5% when compared to manual counts.

**Conclusions:** The aerosolization of grass pollen in our AEC demonstrated naturalistic exposure conditions which did not cause significant rupture of the pollen. Osmotic rupture was induced by staining after pollen was collected, whereas, mechanical rupture can be induced during collection by mechanisms which increase mechanical stresses. Additional data will be collected on ruptured pollen to distinguish featural differences between osmotic and mechanical rupture to advance our characterization algorithm.

**References:**

1. Marks GB, Colquhoun JR, Girgis ST, Koski MH, Treloar AB, Hansen P, Downs SH, Car NG. Thunderstorm outflows preceding epidemics of asthma during spring and summer. Thorax. 2001 Jun 1;56(6):468–71.
2. D’Amato G, Holgate ST, Pawankar R, Ledford DK, Cecchi L, Al-Ahmad M, Al-Enezi F, Al-Muhsen S, Ansotegui I, Baena-Cagnani CE, Baker DJ. Meteorological conditions, climate change, new emerging factors, and asthma and related allergic disorders. A statement of the World Allergy Organization. World allergy organization journal. 2015 Dec;8:1–52.

## 36 The DAM-C study: a survey study on drug allergy management in Canada

### Sinthiha Krishnan^1^, Samira Jeimy^6^, Lana Rosenfield^2^, Juan C. Ruiz^3^, Christine Song^5^, Matthieu Picard^4^, Erika Lee^5^

#### ^1^University of Toronto, Faculty of Medicine, Toronto, ON, ^2^University of Manitoba, Winnipeg, MB, ^3^University of British Columbia, Vancouver, BC, ^4^University of Montreal, Montreal, QC, ^5^University of Toronto, Department of Medicine, Toronto, ON, ^6^Western University, Department of Medicine, London, ON

##### **Correspondence:** Sinthiha Krishnan, Erika Lee

*Allergy, Asthma & Clinical Immunology* 2025, **21(Suppl 1)**:36

**Background:** Unverified drug allergy labels can negatively impact patient care. Though drug allergy testing can effectively clarify the diagnosis, it can also be costly and time-consuming. This study aimed to characterize the current practices of drug allergy testing and identify barriers to testing among allergists in Canada.

**Methods**: A structured online survey was distributed to members of the Canadian Society of Allergy and Clinical Immunology to evaluate drug allergy testing practices. Participation was voluntary and anonymous, with survey data collected and analyzed using REDCap software at the University of Toronto.

**Results:** Of the 65 respondents, most practice in Ontario (41%) and Quebec (39%). Of these, 28 (43%) allergists reported both community and hospital-based practices, with 37% and 20% practicing solely in each respective setting. Most allergists (n = 62) conduct at least one drug allergy consultation per week and perform allergy testing in their offices (n = 52). Regardless of the practice settings, allergists conduct various drug allergy skin testing, which range from 22%-55% in community to 53–81% in hospital-based clinics. They also perform drug challenges, with 80% in community practice and 92% in hospital settings. Barriers to office-based drug allergy testing include insufficient nursing support (n = 27, 63%), lack of access to required drugs for skin testing (n = 24, 56%), high costs of drug reagents (n = 21, 49%), and billing challenges (n = 19, 44%). Barriers to observed drug challenges include billing issues (n = 23, 55%), concerns regarding hospital access (n = 18, 43%), lack of access to necessary drugs (n = 10, 24%), and inability to perform skin testing (n = 8, 19%).

**Conclusions:** Our study showed that although a majority of allergists conduct various types of drug allergy testing in their offices, skin testing and challenges are more commonly performed in hospital-based clinics. The main barriers to drug allergy testing are a lack of resources, personnel and financial support.

## 37 Comparison between pediatric and adult mastocytosis

### Sundus M. NoorSaeed^1,2^, Roy Khalaf^3^, Abdulaziz S. Alrafiaah^1,4^, Barbara Miedzybrodzki^5^, Elena Netchiporouk^5^, John Sampalis^6^, Michael Fein^7^, Moshe Ben-Shoshan^1^

#### ^1^Department of Pediatrics, Division of Allergy and Clinical Immunology and Dermatology, Montreal Children’s Hospital, Montreal, QC; ^2^Department of Pediatrics, King AbdulAziz University Hospital, Jeddah, Saudi Arabia; ^3^Faculty of Medicine, McGill University, Montreal, QC; ^4^Department of Pediatrics, College of Medicine, Majmaah University, Riyadh, Saudi Arabia; ^5^Division of Dermatology, McGill University Health Centre, Montreal, QC; ^6^Division of Surgical Research, Surgical Epidemiology, McGill University, Montreal, QC; ^7^Division of Adult Allergy & Clinical Immunology, McGill University Health Centre, Montreal, QC

##### **Correspondence:** Sundus M. NoorSaeed

*Allergy, Asthma & Clinical Immunology* 2025, **21(Suppl 1)**:37

**Background: Introduction**: Mastocytosis is a rare disorder characterized by the abnormal accumulation and proliferation of mast cells in various tissues. Mastocytosis is classified as cutaneous (CM) or systemic (SM). Data on the clinical presentation and management of mastocytosis are sparse.

**Objective:** We aimed to compare the clinical characteristics, flare-up triggers, and management of CM and SM in children versus adults.

**Methods**: Children and adults with mastocytosis were recruited from dermatology and allergy clinics at the Montreal Children’s Hospital and Montreal General Hospital respectively. Data were collected at study entry on demographics, clinical characteristics, and management by a standardized questionnaire.

**Results:** We recruited 71 participants; 47 children (younger than 18 years old) and 24 adults. Among children; the median age was 2.6 (1.4–6.7) versus 49.3 (38.3–56.8) among adults. The majority of children (63.8%) were males versus (33.3)% males among adults. The majority of patients in both groups presented with CM (94.4% among pediatric and 54.2% among adults). SM was present in 5.6% of children and 45.8% of adults. The major causes of flares reported in children were food (30%), trauma (19%), and medication (11%). Among adults the main causes of flare were medication (54.2%), food (54.2%), alcohol (50%), and trauma (33.3%). Antihistamines were the most common medications used in children (36%) and in adults (46%) followed by adjunctive steroid therapy during flares (13.8% and 20.8% respectively). Omalizumab was administered to one pediatric patient and two adult patients.

**Conclusions:** In both pediatric and adult populations, the main form of mastocytosis is cutaneous. Mainstay treatment antihistamine and topical corticosteroid. We aim to continue and expand the registry to acquire data on the characteristics, management, and triggers of mastocytosis among Canadians.

## 38 Authorship gender diversity in allergy and immunology: analysis of articles published in the Canadian Journal of Allergy, Asthma & Clinical Immunology

### Ranya Al Jumaily^1^, Natalie DeGruse^2^, M. Elise Graham^3^, Samira Jeimy^4^

#### ^1^Department of Internal Medicine, Western University, London, ON; ^2^Schulich School of Medicine and Dentistry, Western University, London, ON; ^3^Department of Otolaryngology-Head and Neck Surgery, Schulich School of Medicine and Dentistry, London, ON; ^4^Division of Clinical Immunology & Allergy, Department of Medicine, Western University, London, ON

##### **Correspondence:** Ranya Al Jumaily, Natalie DeGruse

*Allergy, Asthma & Clinical Immunology* 2025, **21(Suppl 1)**:38

**Background:** Gender diversity in authorship is crucial for providing varied perspectives and equitable representation in medical research. While previous studies have explored gender diversity in authorship within various medical journals, there has been limited focus on Allergy journals. This study aims to address this gap by examining trends in women authorship within the Canadian journal of Allergy, Asthma & Clinical Immunology (AACI) from 2011–2023.

**Methods**: Using the Web of Science database, we retrieved metadata for articles published in AACI from 2011–2023. Gender prediction tools were applied to determine author gender. Cases with uncertain predictions underwent additional online searches. Authors whose gender remained undetermined were excluded from analysis.

**Results:** Out of 929 publications, 38 were excluded due to undetermined author gender. 891 articles with 5541 total authors were included. Women constituted 43.5% (2410) of total authors, 50.8% of first authors (453), 33.8% of last authors (301), and 29.4% of single authors (5). Over time, there was an increase in women as first authors (42.9% in 2011 to 59.6% in 2023; p = 0.16) and last authors (14.3% in 2011 to 46.2% in 2023; p = 0.006), with a significant rise in last authors. Notably, the proportion of women-women first and last authorship pairs significantly increased from 4.8% in 2011 to 26.9% in 2023 (p = 0.03). Articles with women as last authors were significantly associated with women as first authors (OR = 1.56, p = 0.002), and more recent (< 5 years) publications (OR = 1.47, p = 0.009). They were less likely to be authors of review articles (OR = 0.65, p = 0.41).

**Conclusions:** Our findings highlight positive trends towards gender parity in AACI authorship roles. Despite progress, disparities persist among last authors, single authors, and review articles. The association between women first and last authors suggests potential mentorship and collaborative trends that could further bridge the gap. Continuous efforts are crucial to advancing gender equity in allergy and immunology research.

## 39 Gender and geographic representation in editorial boards of allergy and immunology journals

### Ranya Al Jumaily^1^, Natalie DeGruse^2^, M. Elise Graham^3^, Samira Jeimy^4^

#### ^1^Department of Internal Medicine, Western University, London, ON; ^2^Schulich School of Medicine and Dentistry, Western University, London, ON; ^3^Department of Otolaryngology-Head and Neck Surgery, Schulich School of Medicine and Dentistry, London, ON; ^4^Division of Clinical Immunology & Allergy, Department of Medicine, Western University, London, ON

##### **Correspondence:** Ranya Al Jumaily, Natalie DeGruse

*Allergy, Asthma & Clinical Immunology* 2025, **21(Suppl 1)**:39

**Background:** Previous studies have explored gender and geographic diversity across academic disciplines’ editorial boards, yet no such investigation has focused specifically on Allergy and Immunology journals. This study aims to fill this gap by examining gender and geographic disparities among editorial board members of Allergy and Immunology journals.

**Methods**: Using the Clarivate Journal Citation Reports database, Allergy and Immunology journals were identified, and editorial board members were sourced from their respective websites. Data collected included names, roles, affiliations, locations, and predicted gender based on the genderize.io database tool. Pearson correlations were used to assess relationships between journal metrics and editorial board characteristics.

**Results:** 36 journals were analyzed, encompassing 1,546 editorial board members. Notably, the study found no information suggesting representation of non-binary and transgender individuals among editorial board members. Women editors comprised 23.4% of editors-in-chief (11/47), 36.89% of deputy/associate editors (90/244), and 32.99% of other editorial board members (414/1,255).

Most editorial boards (83.33%) were predominantly men (> 55% men), with only 8.33% having a women majority and another 8.33% achieving gender parity (45–55% women). Journal Impact Factor showed no significant correlation with the proportion of women editors (r = -0.15, p = 0.376).

Countries with the highest number of editors included the United States (348, 22.51%), Poland (136, 8.8%), South Korea (102, 6.6%), Spain (94, 6.08%), and Germany (91, 5.89%). In these countries, the proportions of women editors were 34.77%, 33.82%, 37.25%, 29.79%, and 19.78%, respectively. Developed countries accounted for 84.86% of editors (n = 1,312), while developing countries constituted 15.14% (n = 234). Proportions of women editors were 31.78% in developed countries and 41.88% in developing countries.

**Conclusions:** This study reveals significant gender and geographic imbalances within Allergy and Immunology journal editorial boards. Addressing these disparities is essential for fostering inclusivity and equity in academic publishing. Future initiatives should prioritize enhancing diversity by creating more opportunities for underrepresented groups in editorial roles.

## Case Reports*

## 40 Anaphylaxis to bivalent omicron COVID-19 vaccine: a case report in a 59-year-old woman

### Ranya Al Jumaily^1^, Mark Kuprowski^2^

#### ^1^Department of Internal Medicine, Western University, London, ON; ^2^Division of Clinical Immunology & Allergy, Department of Medicine, Western University, London, ON

##### **Correspondence:** Ranya Al Jumaily

*Allergy, Asthma & Clinical Immunology* 2025, **21(Suppl 1)**:40

**Background:** Anaphylactic reactions to COVID-19 vaccines are rare but have been reported. We present a case of a 59-year-old woman, with no previous history of vaccine reactions, who experienced anaphylaxis following her fourth dose of the Bivalent Omicron vaccine.

**Case Presentation:** We describe a case of a 59 year old woman, with previous allergic history of mild seasonal allergies, who presented withdiffuse diaphoresis, chest tightness, neck angioedema, and presyncope within 5 min of Bivalent Omicron vaccine administration. No hives, facial angioedema, or respiratory symptoms were noted. Immediate epinephrine administration, antihistamines, and steroids led to resolution within 2–3 h. Prior to this, she had tolerated three previous COVID vaccinations without incident. Intradermal skin testing with Pfizer Comirnaty XBB 1.5 0.3 mcg per 0.3 mL 1:100 concentration done at St Joseph’s Allergy & Immunology clinic revealed positive reactions to the Pfizer Comirnaty vaccine, confirming a true allergy.

**Conclusions:** This case highlights that anaphylaxis to COVID-19 vaccines, though rare, can occur even in individuals with no prior history of allergic reactions. Clinicians should be vigilant in identifying and managing such cases, emphasizing the importance of thorough evaluation, personalized patient care, and appropriate follow-up. This, however, does not preclude possibility of future retesting and consideration of administration of alternative vaccines if required.

## 41 Mugwort-spice syndrome in a patient with oseltamivir hypersensitivity reaction

### Ming H. Bi, Lundy McKibbin

#### University of Manitoba, Winnipeg, MB

##### **Correspondence:** Ming H. Bi

*Allergy, Asthma & Clinical Immunology* 2025, **21(Suppl 1)**:41

**Background:** Sensitivities to spices often represent pollen food allergy syndrome, due to cross-reactivity between vegetal proteins and pollens in the *Asteraceae* family. IgE-mediated Oseltamivir reactions are rarely reported; a previous case documents a patient with anaphylaxis to Oseltamivir who was also found to have sensitization to Chinese star anise *(Illicium verum)*, a source of shikimic acid which acts as a precursor in oseltamivir production, as well as mugwort, celery, carrot, and other spices, thereby suggesting celery-carrot-mugwort-spice syndrome as a risk factor for oseltamivir hypersensitivity [1].

**Case Presentation:** We present a second case of a patient who developed a possible IgE-mediated reaction to oseltamivir with celery-carrot-mugwort-spice syndrome. A 66-year-old male was assessed by the allergy service for multiple possible food allergies in the context of eosinophilic esophagitis. His comorbidities include atopic dermatitis and gout. Review of the patient’s allergy history revealed symptoms of rhinitis and a possible reaction to oseltamivir approximately 8–10 years prior, with the patient experiencing immediate facial erythema and edema after the first dose of oseltamivir. Subsequent skin testing was positive with regards to oseltamivir (5 × 5 mm wheal + flare) and mugwort (8 × 8 mm wheal + flare), while Chinese star anise produced no wheal but a flare measuring 7 × 7 mm. Negative control was 0 mm without flare. Serum specific IgE was positive to anise (1.19 kU/L), mugwort (0.91kU/L), parsley (1.86kU/L), carrot (0.7kU/L), celery (0.7kU/L), short ragweed (0.86kU/L), Western ragweed (0.6kU/L), and giant ragweed (1.5kU/L).

Conclusions: IgE-mediated hypersensitivity reactions to oseltamivir, Illicium verum sensitization, and mugwort-spice syndrome may be associated. Further investigation is required into cross-reactivity between Illicium verum and pollens from the Asteraceae family. Testing for Illicium verum, pollens and foods associated with mugwort-spice syndrome in patients with oseltamivir reaction can be considered, especially in individuals with symptoms of environmental inhalant allergies.

## 42 Organizing pneumonia secondary to infliximab in a pediatric patient with ulcerative colitis

### Brian Lee^1^, Isaac Martin^1,2^, Kevin Bax^1^, Samira Jeimy^1^

#### ^1^University of Western Ontario, London, ON; ^2^University of Toronto, Toronto, ON.

##### **Correspondence:** Brian Lee

*Allergy, Asthma & Clinical Immunology* 2025, **21(Suppl 1)**:42

**Background:** Organizing pneumonia (OP) can be secondary to many different etiologies, including drug reactions, infections, and extraintestinal manifestation of inflammatory bowel disease. We present here a case of OP in a pediatric patient with ulcerative colitis (UC) on infliximab.

**Case Presentation:** A 12-year-old female was admitted to the hospital with several weeks of fever, dyspnea, and chest pain. She presented to the hospital previously with similar symptoms, and bilateral patchy infiltrates were found on CT thorax. She underwent several courses of antimicrobials for community-acquired pneumonia without significant improvement.

Past medical history is significant for UC for which she was started on mesalamine and infliximab five months prior. Last dose of infliximab was four weeks prior to admission.

On admission, temperature was 37.7 °C, respiratory rate of 34 breaths/minute, and SpO2 was 94% on room air.

Ceftriaxone was started without significant improvement. Bronchoscopy did not show significant cellularity. Bronchoalveolar lavage cultures were negative for bacteria, mycobacteria, fungal organisms, *Pneumocystis jirovecii*, and cytomegalovirus, as well as a standard panel of respiratory viruses on multiplex PCR. Repeat CT thorax showed worsening bilateral infiltrates without pulmonary emboli.

The Naranjo Adverse Drug Reaction Probability Scale score was 3 for both infliximab and mesalamine, which were held pending infectious workup.

Lung biopsy showed fibroblastic polyps expanding the airspaces consistent with OP. Mesalamine was restarted during the admission to prevent UC flare while infliximab was held. No corticosteroids were given. Repeat CT thorax done two months after admission demonstrated near resolution of the bilateral interstitial infiltrate.

**Conclusions:** To our knowledge, this is the first reported case of OP secondary to infliximab in a pediatric patient, which was resolved simply by withdrawing the offending agent. Diagnosis may be challenging due to competing risk factors, and clinicians prescribing infliximab in this population should remain vigilant about this potential adverse reaction.

## 43 Case study: evolving contributions of cannabis-exposure to peanut allergy development

### Danielle Ben-Shoshan^1^, Nha U. Nguyen-Luu^2^

#### ^1^Brock University, St.Catharines, ON; ^2^Clinique d’allergie et d’asthme de Montréal, Montreal, QC

##### **Correspondence:** Danielle Ben-Shoshan

*Allergy, Asthma & Clinical Immunology* 2025, **21(Suppl 1)**:43

**Background:** Nonspecific lipid transfer proteins (LTPs) represent potent small, heat-stable proteins, found in various plant foods such as fruits, vegetables, nuts, and seeds.Recent studies suggest that LTP-related allergies are affecting patients world-wide(3). The primary sensitizers include different pollen including mugwort (Art v3), Can s3 acquired from marijuana through smoke exposure and Pru p 3 from peach via the oral sensitization. “Ara h 9” is a new member of the LTP allergen family that is an emergent peanut allergen.

**Case Presentation:** A 2-year-old boy presented during August 2023 with redness within minutes of peanut ingestion. Three weeks later, the boy presented with recurrent eyelids swelling after playing outside. The boy’s mother administered Epipen (0.15 mg) during one of these episodes. The boy began oral immunotherapy for peanuts that was stopped due to recurrent episodes of oral itching and abdominal pain.

**Social habits:** The child, who was living with his grandparents until 18 months old, was exposed to passive cannabis smoke from grandmother.

**Confirmatory tests:** Skin tests were positive for peanut, mugwort and cannabis.

Laboratory tests included blood count that revealed mildly elevated eosinophils ( 600/mL), normal tryptase. In addition, tests reported normal total IgE (30kU/L) but.

elevated specific peanut IgE (5.20 kU/L) and elevated sIgE to Ara h 9 (7.98kU/L). Specific IgE to Ara h1, Ara h2, Ara h3 and Ara h8 were negative.

**Conclusions:** In this case two factors, mugwort allergy and cannabis sensitivity, might have contributed to Ara h 9 sensitivity and peanut allergy. Our case highlights that mugwort sensitization can occur at a young age, possibly because mugwort and cannabis share non specific LTP allergens. Ara h9 allergy may be more challenging to desensitize given the continuous inhalant exposure to mugwort and cannabis. There is not much data about LTP desensitization.

## 44 Case series of heterozygous FOXN1 variants with mild immunodeficiency phenotypes found on newborn SCID screening

### Yue Du^1^, Andrew Wong-Pack^2^, Jenny Garkaby^3^

#### ^1^McMaster University Department of Internal Medicine, Hamilton, ON; ^2^McMaster University Department of Allergy and Immunology, Hamilton, ON; ^3^McMaster Children’s Hospital Pediatric Immunology and Allergy, Hamilton, ON

##### **Correspondence:** Yue Du

*Allergy, Asthma & Clinical Immunology* 2025, **21(Suppl 1)**:44

**Background:** FOXN1 belongs to the Foxhead-box (FOX) family of transcription factors and is fundamental for development of mature T-cells in the thymus. Autosomal recessive biallelic variants in the FOXN1 gene have been associated with a severe combined immunodeficiency (SCID) phenotype. In contrast, individuals with a heterozygous variant may demonstrate a less severe phenotype. Patients can present with frequent respiratory infections and associated congenital T-cell lymphopenia which may improve with age. We present two patients with heterozygous variants in FOXN1 presenting with a mild clinical phenotype.

**Case Presentation:** Case Presentation: Probands P1 and P2, ages six and two, are two unrelated pediatric patients referred to the immunology service due to a positive newborn screen for SCID. Both were found to have heterozygous variants in FOXN1. Initial laboratory investigations showed T-cell lymphopenia. In P1 the lymphopenia improved with age. In P2, the lymphopenia has persisted. A surveillance approach was taken to manage both patients as our centre had previous experiences with similar presentations in the past. Both P1 and P2 have only had minor infections including upper respiratory tract illnesses and otitis media. Neither have had severe infections requiring hospitalizations or antibiotics. Additionally, P1 and P2 have tolerated live viral vaccines without any adverse effects.

**Conclusions:** There are increasing early reports of heterozygous FOXN1 variants due to the progressive implementation of newborn screening. Our cases, along with others previously published, illustrate a cohort of patients with mild immunodeficiency and variable lymphopenia. Our series expands the phenotypic and genetic spectrum of patients with heterozygous FOXN1 mutations adding to the growing database of FOXN1 variants. It will be important to continue to identify and monitor these patients to further explore the natural history of these variants and hopefully provide reassurance to families regarding the relatively benign trajectory of this presentation.

## 45 Variable allergenicity of different brands of peanut butter and their impact on primary prevention and early infant oral immunotherapy

### Kavya Yatham^1^, Raymond Mak^2^, Edmond Chan^2^

#### ^1^University of British Columbia, Vancouver, BC; ^2^Division of Allergy and Immunology, Department of Pediatrics, University of British Columbia, BC Children’s Hospital, Vancouver, BC

##### **Correspondence:** Kavya Yatham

*Allergy, Asthma & Clinical Immunology* 2025, **21(Suppl 1)**:45

**Background:** To prevent developing a peanut allergy, current guidelines recommend early introduction of peanut around 4–6 months of age with regular ingestion. We present an infant with regular ingestion of peanut butter who developed anaphylaxis after ingesting another brand.

**Case Presentation:** A 7-month female had successfully tolerated ~ 1/3 teaspoon of Kraft peanut butter, (“extra creamy” 4 g protein/15 g serving) 2–3 times a week since age 5–6 months. However, after licking a tiny amount of Adams peanut butter (4 g protein/15 g serving), she developed perioral urticaria to the chest and hands, tongue swelling with gasping sounds suggesting respiratory compromise, and emesis. Her parents administered 1 dose of epinephrine. She was assessed in the ED and required no further treatment.

Skin prick testing was positive to Adams (5 mm) and peanut extract (3 mm) but negative to Kraft, tree nuts and common seeds (the latter were tested due to hesitancy and to rule out cross-contamination). Peanut sIgE and component testing (Ara h 1, 2, 3, 8, 9) were negative, suggesting fresh food skin testing was more sensitive than sIgE. She was diagnosed with higher threshold peanut allergy, with a clinical reaction related to differences in allergen concentration between various brands of peanut butter. After in-office confirmation of no reaction to 300 mg protein Kraft, we recommended daily Kraft ingestion as maintenance oral immunotherapy with plans to perform oral challenge to Adams. Three months later, repeat skin testing to Adams, 5 other brands, and peanut extract was negative. She was challenged with an age-appropriate serving (~ 1500 mg protein) of Adams and passed.

**Conclusions:** This case suggests variable allergenicity of peanut products may be relevant for primary prevention, infants reacting to only certain brands should undergo fresh food skin testing. Oral immunotherapy with tolerated brands can convert skin test responses and induce tolerance of other brands quicker in infants than in older children.

## 46 Thimerosal hypersensitivity: overcoming barriers to influenza vaccination in Canada – a case report with practical implications

### Madeline Robinson^1^, Lundy McKibbin^2,3^, Gahmeng Khuu^4^

#### ^1^University of Manitoba, Department of Internal Medicine, Winnipeg, MB; ^2^University of Manitoba, Department of Internal Medicine, Section of Allergy & Clinical Immunology, Winnipeg, MB; ^3^University of Manitoba, Department of Pediatrics and Child Health, Section of Pediatric Allergy & Clinical Immunology, Winnipeg, MB; ^4^Shared Health, Allergy & Clinical Immunology, Winnipeg, MB

##### **Correspondence:** Madeline Robinson

*Allergy, Asthma & Clinical Immunology* 2025, **21(Suppl 1)**:46

**Background:** Influenza vaccination is an important preventative health measure with proven benefits to individual patients and their communities. Hypersensitivity to influenza vaccination is rare and can be due to the preservative thimerosal. Vaccine hypersensitivity is diagnosed via intra-dermal skin testing followed by supervised graded administration if indicated.

**Case Presentation:** We present a case of thimerosal hypersensitivity resulting in allergic reaction to the Fluzone Quadrivalent influenza vaccine in a healthy 69-year-old female patient. She was referred to Allergy & Clinical Immunology with a longstanding history of reaction to influenza vaccination, including posterior oropharyngeal edema and pruritis. As a result, she had avoided vaccination for many years. Skin-prick and intradermal testing to the Fluzone Quadrivalent influenza vaccine was positive, but the patient tolerated Graded Administration of the High-Dose Fluzone Quadrivalent vaccine used for patients over age 65. On review of product monographs, it was determined that the Fluzone Quadrivalent vaccine contained thimerosal, but the high-dose version did not. Repeat intradermal testing using the high-dose vaccine was negative, and the patient has since tolerated routine vaccination using the thimerosal-free formulation.

**Conclusions:** Patients with reported reactions to influenza vaccines should undergo allergy testing, followed by graded challenge, to confirm hypersensitivity. While thimerosal hypersensitivity is rare, it can pose a significant barrier to influenza vaccination and thus negatively impact patient health. In Canada, regular strength Fluzone Quadrivalent influenza vaccine contains thimerosal whereas the high-dose formulation recommended for patients over age 65 does not. We note that thimerosal-free versions are readily available for all ages and may be useful in some cases of vaccine allergy. We hope to increase awareness about thimerosal-mediated vaccine reactions and demonstrate an opportunity to avoid future time-consuming and resource-intensive desensitization visits—ultimately safely offering viable alternatives for Canadian patients with thimerosal hypersensitivity.

## 47 Managing mucosal allergic inflammation in a patient with chronic spontaneous urticaria

### Melanie M. Wong, Paul K. Keith

#### McMaster University, Hamilton, ON

##### **Correspondence:** Melanie M. Wong

*Allergy, Asthma & Clinical Immunology* 2025, **21(Suppl 1)**:47

**Background:** Patients with chronic spontaneous urticaria (CSU) often have elevated levels of aeroallergen sensitization, total IgE, and IgG autoantibodies to FcεR1α, IgE, and thyroid peroxidase, suggesting a potential link between CSU and T2-driven inflammatory conditions.

**Case Presentation:** We present an update to a previously described case of a 47-year-old female with a history of recurrent CSU, allergic rhinitis, and intermittent asthma who presented to the ED in December 2019 with lip and tongue angioedema and generalized urticaria. She received intramuscular epinephrine and was referred to Allergy & Clinical Immunology for further workup.

Initial investigations revealed positive autoantibodies to thyroglobulin and dsDNA, normal TSH, low C3, and normal IgE at 11. Her blood eosinophil count was normal at 0.3 at highest, but eosinophil percentage was elevated at 5%. Skin prick tests were positive for grass, ragweed, and dust mite. She was counseled on allergen avoidance strategies and prescribed intranasal fluticasone furoate, montelukast and rupatadine. Her symptoms worsened during the 2020 grass and ragweed pollen seasons, requiring two courses of prednisone. She was offered omalizumab but declined. Treatment was intensified with increased rupatadine dosage and inhaled fluticasone furoate for asthma control. Her CSU symptoms improved by 2021, requiring only one course of prednisone during ragweed season and demonstrating better control during grass season.

A break in the case occurred in 2022 after initiating ragweed and dust mite sublingual immunotherapy. This led to marked improvement in CSU symptoms during the 2023 and 2024 ragweed pollen seasons. Since spring 2022, our patient has not experienced any recurrence of CSU flares.

**Conclusions:** In a patient with a history of allergic rhinitis and aeroallergen sensitization, her CSU symptoms improved with management of mucosal allergic inflammation. The patient’s successful treatment with allergen immunotherapy suggests that specific allergen avoidance and desensitization may play a beneficial role in CSU management.

## 48 Dupilumab in pediatric eosinophilic gastrointestinal disease: a case series

### Ryan Scanlan, Collin Terpstra

#### McMaster University, Hamilton, ON

##### **Correspondence:** Ryan Scanlan

*Allergy, Asthma & Clinical Immunology* 2025, **21(Suppl 1)**:48

**Background:** Eosinophilic gastrointestinal disorders (EGID) in children are challenging due to their variable presentation, impact on growth and lack of approved treatments. Dupilumab, effective in treating EoE by inhibiting TH2-mediated inflammation, is promising in non-EoE EGID due to shared inflammatory pathophysiology.

## Case Presentation:

*Case 1:* A 13-month-old male with severe atopy, including severe eczema food allergy to peanuts and wheat, developed concerns for EGID by 21 months. He required pureed foods and had frequent bowel movements. Blood work and biopsies confirmed EGID, showing elevated eosinophils (900–1500), IgE 1633 and eosinophilic gastric infiltration with 41 eos/hpf. By three years old, he was failing to thrive and relied on prescription meal replacements. Trial of topical ICS had failed. After obtaining Dupilumab at 3 years and 8 months, his food intake dramatically improved within two weeks, subsequent gastric biopsies showed resolution of eosinophilic infiltration. Over a year on Dupilumab, his blood work normalized (Eos 300, IgE 183), and he made significant progress in eating, aided by a multidisciplinary team.

*Case 2:* A 9-month-old male with failure to thrive and gastrointestinal symptoms since 1-month-old required G-tube with hydrolyzed formula to stabilize. Multiple food introductions triggered symptoms like emesis and abdominal pain. Endoscopy demonstrated eosinophilic gastric infiltration with 30 eos/hpf with esophageal sparing, peripheral eosinophils were 300. Initial treatment with Montelukast, topical ICS and focused elimination improved weight, but symptoms returned by age three. Dupilumab was started at age four, leading to significant improvement. The patient increased food intake, reduced G-tube dependency, and normalized stools, after one year of treatment, he was in remission and G-tube was removed.

**Conclusions:** These cases suggest dupilumab’s efficacy in managing pediatric EGID. Reporting clinical outcomes following off-label use of dupilumab in pediatric patients with EGID will contribute to developing evidence-based therapeutic strategies in this area of clinical equipoise.

## 49 Cold-Derm: A proposed simple and simultaneous bedside test for cold-induced urticaria, symptomatic dermatographism, and cold-dependent dermatographism

### Payam Salimi^1^, Julie Hong^1^, Lundy McKibbin^1,2^

#### ^1^Department of Internal Medicine, University of Manitoba, Winnipeg, MB; ^2^Department of Pediatrics and Child Health, University of Manitoba, Winnipeg, MB

##### **Correspondence:** Payam Salimi

*Allergy, Asthma & Clinical Immunology* 2025, **21(Suppl 1)**:49

**Background:** Cold-dependent dermatographism is a rare, atypical form of cold-induced urticaria, where dermatographism is only elicitable on skin that has been previously cooled.[1] Despite having a compelling clinical history of urticaria, patients with isolated cold-dependant dermatographism may have negative cold stimulation and pressure testing if performed separately, leading to diagnostic challenges. Today, aside from sparse case reports, there is limited data regarding cold-dependant dermatographism. [2] A significant proportion (15.3—28.4%) of patients assessed for cold-induced urticaria are noted to have negative cold stimulation testing.[3,4] This suggests, that there may be a significant number of patients with undiagnosed cold-dependant dermatographism.

**Case Presentation:** We present a 32-year-old, otherwise healthy, male with chronic intermittent urticaria for years who underwent a novel bedside test that simultaneously assessed for cold-induced urticaria as **well as** both cold-dependent and independent dermatographism. He notes that urticaria will often occur after cold exposure and after scratching.

The patient underwent the “Cold-Derm” test which involved applying cold stimulation via an ice cube to the patient’s forearm for 5 min. Upon removal of the ice cube, linear pressure is applied above, through, and below the area of cold exposure. Linear pressure is similarly applied on the contralateral forearm (as an ambient temperature control). The test was interpreted 10 min post-provocation (15 min after initial ice cube application). There was marked dermatographism confined only to the area that was cooled, without urticaria seen elsewhere. There was no urticaria on the ambient temperature arm and no urticaria at the site of the ice cube aside in the small area of linear pressure.

**Conclusions:** The patient had testing consistent with cold-dependent dermatographism (with negative cold stimulation and pressure testing when performed in isolation). The “Cold-Derm” test is a simple and accessible bedside test and if adopted, may significantly enhance the diagnosis of cold-dependent dermatographism.

## 50 A case of delayed post hypoxic leukoencephalopathy following anaphylaxis to cow’s milk

### Candice Luo, Kevin Thomas, Lundy McKibbin

#### University of Manitoba, Winnipeg, MB

##### **Correspondence:** Candice Luo

*Allergy, Asthma & Clinical Immunology* 2025, **21(Suppl 1)**:50

**Background:** Anaphylaxis is a potentially fatal systemic allergic reaction with various presentations [1]. Reported consequences from anaphylaxis include increased risk of repeat anaphylaxis, anxiety, and post-traumatic stress [2]. However, to date, there is no reported case of anaphylaxis leading to delayed post hypoxic leukoencephalopathy (DPHL).

**Case Presentation:** A 25-year-old female with known cow’s milk allergy presented to hospital after developing grade IV anaphylaxis from accidental ingestion of a beverage with undisclosed cow’s milk from a popular fast food chain. After developing anaphylaxis, she self-injected with an expired epinephrine auto-injector. Upon presentation to the emergency department, she was found in bradycardic pulseless electrical activity lasting 6 min. She was urgently resuscitated and had a complex hospital stay for a total of 48 days.

On day 8 of admission, she developed numerous neurological symptoms that would be ultimately diagnosed as DPHL via MRI brain imaging. She developed sequelae of DPHL, including seizures and functional limitations leading to decreased mobility, speech impairment, and reduced bilateral upper extremity motor strength. She required prolonged rehabilitation at the Acquired Brain Injury Rehabilitation Unit as an inpatient and has ongoing outpatient rehabilitation with significant functional recovery.

Following discharge, Phadia ImmunoCAP™ serum specific IgE testing was completed for cow’s milk and its components. Results confirmed cow’s milk allergy with alpha lactalbumin at 9.3 KU/L, beta lactoglobulin 8.2 KU/L, casein 21.2 KU/L, whey 16.5 KU/L, cow’s milk 17.2 KU/L. She has since carried two up-to-date Epinephrine auto-injectors at all times without subsequent accidental exposure.

**Conclusions:** Delayed post-hypoxic leukoencephalopathy is an extremely rare syndrome following a hypoxic brain injury that can have life-altering consequences. This case highlights the importance of continued advocacy and patient education on the importance of maintaining up-to-date epinephrine auto-injectors to prevent severe debilitating consequences of anaphylaxis.

## 51 From fruit to infarction: unraveling a kiwi-induced cardiac crisis

### Devyani Bakshi, Jennifer Du, Melanie Wong, Vivian G. Szeto, Susan Waserman

#### McMaster University, Hamilton, ON

##### **Correspondence:** Devyani Bakshi, Melanie Wong.

*Allergy, Asthma & Clinical Immunology* 2025, **21(Suppl 1)**:51

**Background:** Myocardial infarction (MI) secondary to anaphylaxis is an uncommon but serious complication, with epinephrine-induced MI and Kounis syndrome being possible causes. Epinephrine can rarely induce coronary artery spasm, while Kounis syndrome involves MI caused by a hypersensitivity allergic reaction or immune reaction.

**Case Presentation:** A 61-year-old female with poorly controlled type 2 diabetes, dyslipidemia, hypertension, fatty liver disease, and oral allergy syndrome to kiwi developed a MI following an anaphylactic reaction to kiwi. She presented to the emergency department with dyspnea, abdominal pain, nausea, vomiting, and facial flushing within five minutes of consuming a kiwi smoothie. Initial treatment included, 0.5 mg intramuscular epinephrine (1:1000), 40 mg IV methylprednisolone, and 50 mg IV diphenhydramine. She subsequently developed substernal chest pain, and an electrocardiogram showed ST depressions in the inferolateral leads. Her troponin levels increased from 3 ng/L to 317 ng/L, indicating myocardial injury. Complete blood count demonstrated a leukocytosis of 15.2 × 10^9^/L with borderline eosinophils of 0.4 × 10^9^/L and D-dimer of 19,421 mg/L FEU. Electrolytes, creatinine, eGFR, urea, CT pulmonary angiography, and infectious workup were all unremarkable.

She developed a hypertensive emergency, pulmonary edema, and required vasopressors to stabilize her blood pressure. She received 125 mg IV methylprednisolone, aspirin 80 mg, and 2.5 mg fondaparinux. A transthoracic echocardiogram showed normal ejection fraction but mild to moderate valvular abnormalities. Post-discharge, skin prick testing was positive to kiwi and serum specific IgE levels to kiwi was elevated at 4.09kU/L (normal range < 0.35kU/L).

Conclusions: Distinguishing Kounis syndrome from epinephrine-induced vasospasm leading to MI post anaphylaxis can be difficult, especially in patients with medical comorbidities. Given her sensitization to kiwi this may be a case of Type II Kounis syndrome. However, given our patient’s age and medical comorbidities, epinephrine-induced vasospasm was the likely etiology, although it is difficult to be certain. This case highlights the importance of early recognition and management of complications in anaphylaxis.

## 52 An unusual case of burning mouth syndrome (BMS) to toothpaste: association between sodium lauryl sulfate and BMS

### Erin Moskovic^1^, Kyle Seigel^2^, Gordon Sussman^1^

#### ^1^Gordon Sussman Clinical Research Inc., Toronto, ON; ^2^University of Toronto, Toronto, ON

##### **Correspondence:** Erin Moskovic

*Allergy, Asthma & Clinical Immunology* 2025, **21(Suppl 1)**:52

**Background:** Burning mouth syndrome (BMS) is a condition characterized by a burning sensation on the tongue, dryness of the mouth, and alteration of taste [1]. The etiology of BMS is largely unknown, but case studies have suggested there may be a relationship between sodium lauryl sulfate, a common surfactant agent found in toothpaste, and the development of inflammatory reactions of the tongue [2]. We hypothesize that sodium lauryl sulfate may contribute to the onset of BMS in patients who use toothpaste containing the detergent.

**Case Presentation:** A 73-year-old woman was referred to the clinic for evaluation of BMS caused by the use of toothpaste. In November of 2023, after using toothpaste the patient experienced symptoms of dry and swollen lips, a burning and itchy sensation of the tongue, and altered taste perception due to a white film in the mouth. The patient noted that various toothpastes induced the BMS reaction, including Arm & Hammer, Crest, Tom’s of Maine, and Colgate toothpastes. A patch test revealed a positive result to the toothpastes, causing symptoms of vesicles, itchiness, and redness to appear on the skin that persisted for 48 h. It should be noted that sodium lauryl sulfate is a common ingredient among all mentioned toothpastes, and therefore may be a likely cause of BMS experienced by the patient.

**Conclusions:** Our results suggest that sodium lauryl sulfate may contribute to the development of BMS. Studies are needed to further investigate the underlying mechanism of sodium lauryl sulfate in its ability to trigger this painful and uncomfortable condition.

**References:**

1. Grushka M, Epstein JB, Gorsky M. **Burning Mouth Syndrome.** *Am Fam Physician.* 2002;**65**:615–621.
2. Brown RS, Smith L, Glascoe AL. **Inflammatory Reaction of the Anterior Dorsal Tongue Presumably to Sodium Lauryl Sulfate within Toothpastes: A Triple Case Report.** *Oral Surg Oral Med Oral Pathol Oral Radiol.* 2018;**125(2)**:E17-E21.

## 53 COVID-19-induced cold urticaria in the adult population

### Erin Moskovic, Gordon Sussman

#### Gordon Sussman Clinical Research Inc., Toronto, ON

##### **Correspondence:** Erin Moskovic

*Allergy, Asthma & Clinical Immunology* 2025, **21(Suppl 1)**:53

**Background:** Cold urticaria is a condition characterized by angioedema, redness, itchiness, and raised welts on the skin when exposed to the cold [1]. Pediatric studies have investigated the link between COVID-19 infection and urticaria in children [2], but little is reported on virus-induced urticaria in the adult population. We report a case of an adult patient presenting with a condition of cold urticaria after infection with COVID-19.

**Case Presentation:** A 47-year-old woman developed various skin eruptions following repetitive exposure to the cold. The patient has minimal prior history of reactions to cool temperatures, experiencing minor symptoms of redness after about 45 min of exposure to the cold. In December of 2023, the patient was infected with COVID-19 and experienced intensified reactions to the cold thereafter. In the winters, in rooms with air conditioning, and on cooler summer days, the patient experiences severe welts, redness, itchiness, and intense pain after 5–6 min of exposure to the cold. A TempTest was used to assess the presence and level of severity of cold-induced urticaria. The test results showed that the patient experienced skin eruptions to the temperature threshold (27 degrees Celsius), indicating the presence of severe cold urticaria. The results suggest that exposure to COVID-19 may have contributed to the onset of cold urticaria in the patient.

**Conclusions:** Our results show that infection with COVID-19 in the adult population may be associated with the development of cold urticaria. On-going research is needed to investigate and better understand any potential relationship between virus-induced cold urticaria in the adult population.

References

1. Claudy A. **Cold Urticaria.** *J Investig Dermatol Symp Proc.* 2001;**6(2)**:141–142.
2. Panda M, Agarwal A, Hassanandani T. **Dermatological Manifestations of COVID-19 in Children.** *Indian Pediatr.* 2022;**59**:393–399.

## 54 A case of relapsing Drug Reaction with Eosinophilia and Systemic Symptoms (DRESS) with severe peripheral hypereosinophilia responding to IV methylprednisolone

### Jung Min (Julie) Hong^1^, Karver Zaborniak^1^, Lundy McKibbin^2^

#### ^1^University of Manitoba Department of Internal Medicine, Winnipeg, MB, ^2^Department of Internal Medicine, Pediatrics and Child Health, Winnipeg, MB

##### **Correspondence:** Jung Min (Julie) Hong

*Allergy, Asthma & Clinical Immunology* 2025, **21(Suppl 1)**:54

**Background:** Drug reaction with eosinophilia and systemic symptoms (DRESS) is a delayed T-cell-mediated hypersensitivity with onset 2–8 weeks after culprit drug exposure and a mortality rate of 3–10%. [1] Relapse can occur despite cessation of culprit drug exposure. When accompanied by severe peripheral hypereosinophilia, the increased risks of end organ damage and thromboembolic complications typically warrant use of systemic glucocorticoids. [2] Research is lacking on the ideal dose, route and type of glucocorticoids.

**Case Presentation:** A 48-year-old male of Asian descent with history of gout and metabolic dysfunction-associated steatotic liver disease (MASLD) was diagnosed with DRESS, (RegiSCAR = 9) after presenting with facial edema, diffuse pruritic maculopapular rash, transaminitis and acute kidney injury in the setting of allopurinol exposure. Despite initial improvement on oral prednisone 60 mg daily and strict allopurinol avoidance, he had severe clinical relapse within 2 weeks. While readmitted to hospital and maintained on uninterrupted 60 mg of prednisone daily, his eosinophil count rose from 1.22 × 10^9^/L to 15.94 × 10^9^/L (normal range 0.0–0.4 × 10^9^/L) over the course of 7 days. Hypereosinophilia of this severity raises potential for life- and organ-threatening sequelae. While other non-traditional immunosuppressive regimens were considered, a change from oral prednisone 60 mg to an equivalent IV methylprednisolone dose was initiated by the allergist on call. Within 12 h of change to methylprednisolone, the peripheral eosinophil count dramatically decreased, returning to normal range, 0.33 × 10^9^/L, within 5 days. The patient was discharged home shortly thereafter.

**Conclusions:** Relapsing DRESS with severe peripheral hypereosinophilia that proves unresponsive to oral prednisone may rapidly improve with change to equivalent dosing of methylprednisolone. The rapid response to methylprednisolone is hypothesized to either be a consequence of more optimal hepatic metabolism or due to increased bioavailability via the IV route as a consequence of gut edema.

## 55 Alpha-gal syndrome: a case report from the Canadian Prairies

### Molly Rayner, Gregory Peters

#### University of Saskatchewan, Saskatoon, SK

##### **Correspondence:** Molly Rayner

*Allergy, Asthma & Clinical Immunology* 2025, **21(Suppl 1)**:55

**Background:** Alpha-gal syndrome (AGS) is an IgE mediated allergy to galactose-alpha-1,3-galactose, also called alpha-gal. This condition is characterized by delayed allergic reactions following consumption of mammalian meat. In North America, the primary cause of AGS is tick bite exposure to the lone star tick. Historically, the lone star tick was found in the eastern and southern United States, but more recently has expanded its geographic range due to climate change. It is suggested that future cases of AGS could be detected in Canada, where the lone star tick is not currently established.

**Case Presentation:** We describe a case of a 72-year-old Saskatchewan female referred for assessment of urticaria. She experienced a tick bite two years earlier. Six months after the bite, she developed episodes of urticaria. Her hives would occur within 2 h following ingestion of mammalian meat, and other reactions included facial flushing, shortness of breath, presyncope and nausea. Prior to her bite she tolerated red meat with no reactions. She had not travelled to the United States in over 5 years and did not recall getting a tick bite outside of Saskatchewan. Serum specific IgE testing showed she was strongly positive to galactose-alpha-1,3-galactose with an IgE > 100 kU/L. She was advised to avoid mammalian meat and carry an epinephrine autoinjector.

**Conclusions:** Currently there are no written case reports of AGS in the Canadian Prairies. The lone star tick is the primary cause of AGS in North America, but various tick species have been linked to AGS worldwide. It is possible that other tick species could contribute to AGS. With the geographic expansion of tick species, an increase in tick-borne diseases, including AGS, is predicted. This highlights the importance of considering AGS as a cause for recurrent urticaria and the need for reporting AGS cases to determine incidence rates in Canada.

## 56 Mystery anaphylaxis—a case of peanut and fenugreek cross-reactivity

### Navjeet Gill, Irene Chair, Ryan Lo

#### Allergy and Immunology, University of British Columbia, Vancouver, BC

##### **Correspondence:** Navjeet Gill

*Allergy, Asthma & Clinical Immunology* 2025, **21(Suppl 1)**:56

**Background:** Fenugreek is a member of the *Leguminosae* family of legumes commonly used in Indian and Mediterranean cuisine. Fenugreek is a rare food allergen with few cases reported in the literature. Homology among fenugreek and peanut allergens has been reported.

**Case Presentation:** A 13-year-old boy with confirmed peanut allergy (SPT 12 × 4 mm, IgE > 100 kIU/L) and a medical history of asthma and allergic rhinitis developed three episodes of anaphylaxis after eating Indian food. Two of these episodes required intramuscular epinephrine and emergency room visit. No clear exposure to peanut was identified in these episodes. There was concern regarding allergy to spices in curry. Initial suspected allergen was tamarind, which yielded negative skin prick testing to fresh sample. Skin prick testing to a variety of fresh spices subsequently was positive to fenugreek (7 mm × 5 mm) and curry mix (5 mm × 5 mm); and negative to ginger, turmeric and cumin. Fenugreek is a common spice used in Indian cuisine which unifies his previous anaphylactic episodes.

**Conclusions:** Fenugreek sensitization is believed to be a consequence of cross-reactivity in patients with peanut allergy. Peanut is known to be widely-cross reactive with legumes, such as lupin, and foods outside the legume family. We highlight cross-reactivity between peanut and fenugreek, lupin, tree nuts, and sesame seeds. We present a case of cross-reactivity between peanut and fenugreek, an infrequently reported cross-reactivity in the legume family. Peanut proteins have high cross-reactive potential beyond the legume family and clinicians should keep a high index of suspicion for cross-reactivity in peanut-allergic patients.

## 57 A case of pollen food allergy syndrome in Nunavik

### Nofar Kimchi, Michelle Kwok

#### McGill University, Montreal, QC

##### **Correspondence:** Nofar Kimchi, Michelle Kwok

*Allergy, Asthma & Clinical Immunology* 2025, **21(Suppl 1)**:57

**Background:** Climate change is expected to increase global temperatures and alter aeroallergen distribution, posing a significant threat to human health by impacting respiratory and food allergies (1–4).

We present a 14-year-old female from Nunavik, Northern Quebec, with Pollen Food Allergy Syndrome (PFAS) to birch, tree, and ragweed pollen, despite living above the tree line.

**Case Presentation:** A 14 year old girl who presented with recurrent angioedema after consuming various fruits including apricot and banana as well as tree nuts. Family history includes a cousin with PFAS to tree pollen, hazelnut, pistachio, kiwi, and orange. Initial reaction involved eyelid edema following peanut ingestion and cold exercise, treated with certizine. Subsequent episodes included lower lip edema after mango and orange intake, managed with diphenhydramine. Pruritus post-exposure to orange and peach juice was alleviated with diphenhydramine.

Her skin prick test was positive for birch, ragweed, 11-tree mix, hazelnuts, almonds, Brazil nut, pecan, sesame, bananas, orange, kiwi, apricot, and cucumber. An oral apricot challenge induced facial hives and angioedema, resolving within an hour post-30 mg cetirizine. The challenge used incremental doses up to 1 whole apricot.

**Conclusions:** Climate change impacts pollen allergenicity and distribution. Studies on ragweed in Winnipeg and Saskatoon showed a 27-day extension of the pollen season between 1995 and 2009 (5). American research demonstrated that birch trees have begun pollinating 1–2 weeks earlier, with increases in annual mean and daily peak pollen concentrations (6). Understanding these shifts is crucial for predicting and managing allergy symptoms, especially in regions previously unaffected by these allergenic plants.

Changes in pollen exposure over time are expected to alter sensitization patterns, particularly affecting Indigenous and remote populations. It is crucial to intensify our management approaches and strengthen healthcare providers’ capabilities. Collaboration with other healthcare professionals, environmental scientists, and policymakers are necessary to address these changes effectively.

## 58 Dupilumab-induced hypereosinophilia treated with mepolizumab: a case report

### Ryan Gauld^1^, Jumana Sarraj^1^, Harold Kim^1,2^

#### ^1^Western University, London, ON; ^2^McMaster University, Hamilton, ON

##### **Correspondence:** Ryan Gauld

*Allergy, Asthma & Clinical Immunology* 2025, **21(Suppl 1)**:58

**Background:** Dupilumab is a fully humanized anti-interleukin-4 receptor alpha monoclonal antibody that blocks interleukin-4 (IL4) and interleukin-13 (IL13) signaling. It is well known that dupilumab can cause eosinophilia in 1 – 14% of clinical trial participants and up to 27.8% in real world studies [1]. Dupilumab likely blocks eosinophil migration into tissue by inhibiting the production of eotaxin and vascular-cell adhesion molecules mediated by IL4 and IL13 [2]. While most cases of eosinophilia are asymptomatic and self-limiting, some can persist for up to 12 months, with a few reports of dupilumab-induced hypereosinophilia causing clinical manifestations and organ damage [1,3]. Current management strategies recommend oral corticosteroids for dupilumab-induced hypereosinophilia with blood eosinophils ^3^ 3.0 × 10^9^/L [1]. However, the associated morbidity and mortality of corticosteroid use necessitates the identification of alternative treatments [4]. Here, we present the first known case of dupilumab-induced hypereosinophilia managed with a single dose of mepolizumab.

**Case Presentation:** A 10-year-old female with eosinophilic esophagitis, allergic rhinitis, eczema, and severe asthma developed hypereosinophilia following initiation of dupilumab. She previously failed high-dose inhaled corticosteroids, omalizumab, and mepolizumab. Her baseline eosinophil count was 1.3 × 10^9^/L, which decreased to 0.4 – 0.9 × 10^9^/L on mepolizumab. Upon transitioning to dupilumab 200 mg subcutaneously every 2 weeks, her eosinophil count rose to 4.3 × 10^9^/L. Despite the eosinophilia, she remained stable without symptoms of eosinophilic tissue infiltration. There was one of her doses of mepolizumab remaining. She received this single dose of mepolizumab 40 mg subcutaneously, reducing her eosinophil count to 0.5 × 10^9^/L within one month.

**Conclusions:** In this case, mepolizumab was likely effective in treating the dupilumab-induced hypereosinophilia. Long-term follow up is necessary to determine if a single dose of mepolizumab is sufficient for a sustained response.

## 59 Hypereosinophilia associated with gluteal intramuscular myxoma

### Sheikha Alkhudher, Harold Kim, Karl Maxemous

#### Western University, London, ON

##### **Correspondence:** Sheikha Alkhudher

*Allergy, Asthma & Clinical Immunology* 2025, **21(Suppl 1)**:59

**Background:** Intramuscular myxoma is one of the rarest benign soft tissue tumours in the musculoskeletal system, occurring between the fourth and seventh decade in female patients. It can be found in the large muscles of the thigh and diagnosed by histopathological testing. The definitive treatment is surgical excision with rare local recurrence reported. The association between intramuscular myxoma and abnormal peripheral blood cell counts has been scarcely reported. There are few reported cases of eosinophilia associated with cardiac myxomas. We report a case of hypereosinophilia associated with gluteal intramuscular myxoma.

**Case Presentation:** A 68-years-old Caucasian female patient with newly diagnosed uncontrolled asthma, unintentional weight loss, fatigue, non-specific chest pain, and paresthesia was referred to our clinic for the assessment of hypereosinophilia with peak level at 3.8 × 109/L. Several tests were carried out to rule out causes of hypereosinophilia including CT Abdomen/Pelvis that revealed a cystic lesion in the right gluteus maximus that was assessed further by MRI Pelvis with gadolinium. It showed a large right gluteal lesion that’s complex, cystic and solid. The lesion’s biopsy confirmed the diagnosis of intramuscular myxoma. In the interim, despite medical treatment and seeing various subspecialities to investigate and treat her symptoms, her condition continued to worsen. She eventually underwent surgical resection of the intramuscular myxoma. Soon after, her eosinophil count started to decline until it normalized and her symptoms dramatically resolved with no further recurrence of her symptoms or the tumor.

**Conclusions:** The patient’s hypereosinophilia and associated symptoms can be attributed to an intramuscular myxoma. There’s extensive literature demonstrating the association between eosinophilia and a variety of other pathologies including solid tumors and haematological malignancies. As far as we are aware, this is the first reported case of hypereosinophilia and associated symptoms attributed to a gluteal intramuscular myxoma. Myxoma should be considered a potential cause of Hypereosinophilia.

## 60 Two cases of acquired angioedema: one with splenic lymphoma and the other with Waldenström’s macroglobulinemia

### Suzanne Kelly^1^, Hazelyn Torres^2^, Andrew Comiskey^1,3^, Duazylle Torres^2^, Stephanie Santucci^2^, William Yang^1,2,4^

#### ^1^Red Maple Trials Inc., Ottawa, ON; ^2^Ottawa Allergy Research Corporation, Ottawa, ON; ^3^Emory University, Atlanta, GA, USA; ^4^University of Ottawa, Ottawa, ON

##### **Correspondence:** Stephanie Santucci

*Allergy, Asthma & Clinical Immunology* 2025, **21(Suppl 1)**:60

**Background:** Acquired angioedema with C1-inhibitor deficiency (AAE-C1-INH) is a rare condition resembling hereditary angioedema (HAE), but with low or dysfunctional C1-inhibitor (C1-INH) resulting from either due to autoantibody binding or lymphoproliferative diseases, mainly lymphatic malignancies. Being about tenfold rarer than HAE it is believed that AAE-C1-INH prevalence is between 1:100,000and 1:150,000. There is very limited knowledge about this disorder and no approved therapies for this condition.

**Case Presentation:** We present two cases of acquired angioedema, one patient diagnosed with splenic lymphoma and the other with Waldenstrom’s macroglobulinemia.

*Case 1:* An 83-year-old male a with history of abdominal pain and distention, facial and tongue swelling unresponsive to H1-antihistamines and oral steroids, absent C4, C1INH and CH 50. C1q was 38 (N:83–125) ug/L. He was found to have splenic lymphoma and was treated with radiation therapy. Lymphoma went into remission and angioedema subsided. Lymphoma and angioedema recurred, was treated with further radiation and the angioedema subsided again.

*Case 2:* A 52-year-old female presented with episodes of severe abdominal pain, vomiting, and swelling of hands, feet, lips and buttocks. C4 was < 0.01 (N:0.1–0.4 g/L), C1-INH 0.08 (N: 0.21–0.39 g/L), IgM 8.3 (N 0.4–2.3 g/L), IgG 4.3 (N:7–16 g/L), IgM-ACL > 150 (N: < 12 units), CRP 81 (N: < 10). She received eight 21-day cycles of combination chemotherapy for her lymphoplasmacytic lymphoma with rituximab (375 mg/m^2^), cyclophosphamide (750 mg/m^2^) and prednisone (60 mg/m^2^). She completed the treatment and received maintenance therapy with rituximab (375 mg/m^2^) every 3 months for 2 years. Her C4, C1-INH, serum IgM, IgG, IgM-ACL and CRP all returned to normal levels. Clinically, her angioedema and other symptoms have all subsided.

**Conclusions:** Acquired angioedema is a very rare occurrence with lymphoproliferative disorder. Treating the underlying disorder with radiation therapy and rituximab may lead to complete resolution of acquired angioedema.

## 61 Mepolizumab is effective in Eosinophilic Granulomatosis with Polyangiitis (EGPA) and Eosinophilic Asthma: report of two cases

### Andrew Comiskey^1,3^, Suzanne Kelly^1^, Stephanie Santucci^2^, Hazelyn Torres^2^, Duazylle Torres^2^, William Yang^1,2,4^

#### ^1^Red Maple Trials Inc., Ottawa, ON; ^2^Ottawa Allergy Research Corporation, Ottawa, ON; ^3^Emory University, Atlanta, GA, USA; ^4^University of Ottawa, Ottawa, ON

##### **Correspondence:** Stephanie Santucci

*Allergy, Asthma & Clinical Immunology* 2025, **21(Suppl 1)**:61

**Background:** Eosinophilic granulomatosis with polyangiitis, (previously Churg-Strauss syndrome) is characterized by bronchial asthma, sinusitis, pulmonary filtrates, neuropathy and eosinophilic vasculitis of one or more end-organs. Eosinophils are thought to induce pathogenic effects in patients with eosinophilic polyangiitis via tissue and vascular infiltration and inflammation through a variety of mediators. Although systemic corticosteroids form the cornerstone of treatment for EGPA, most patients remain dependent on glucocorticoid therapy and relapses are common.

**Case Presentation:** We report two cases of EGPA treated with mepolizumab.

C*ase 1*: A 61-year-old female with history of poorly controlled, steroid dependent eosinophilic asthma with frequent exacerbations, requiring oral prednisone 30 mg daily, hyper-eosinophilic syndrome and type II diabetes as a result of oral prednisone use. IgE was 216 IU, eosinophilic count was 9.36*10 ^9^/L. She had arthralgia and peripheral neuropathy. She started on mepolizumab 300 mg every four weeks. Her asthma was well controlled with no further exacerbations and her EGPA went into remission. Prednisone is now unnecessary.

*Case 2*: A 78-year-old male with initial history of severe allergic asthma with many exacerbations requiring oral prednisone. IgE was 506 IU. Spirometry showed airway obstruction. He responded well to omalizumab subcutaneously 300 mg every 4 weeks. Eight years later, his asthma exacerbated and he developed severe peripheral neuropathy with difficulty walking. Eosinophil was 3.7 (N: 0.0–0.5 *10^9^/L), ESR was 27, CRP 27.5(N: < 8 mg/L), ANCA was positive, MPO antibodies were 34.2 (N: < 5.0 U/ml). Omalizumab was discontinued and mepolizumab 300 mg every four weeks plus methotrexate was started. His asthma became well controlled and neuropathy improved. He is able to walk and play golf.

**Conclusions:** The following two cases demonstrate that mepolizumab is effective in eosinophilic asthma and EGPA. Both patients are off oral corticosteroids and their quality of life has improved significantly.

## 62 A case of drug-induced enterocolitis syndrome with amoxicillin

### Tess Robart, Mary McHenry.

#### Dalhousie University, Halifax, NS

##### **Correspondence:** Tess Robart

Allergy, Asthma & Clinical Immunology 2025, **21(Suppl 1)**:62

**Background:** Drug-Induced Enterocolitis Syndrome (DIES) is a rare, non-IgE mediated drug-induced hypersensitivity reaction involving the gastrointestinal system. On review of existing literature, a total of 12 cases of DIES have been published. DIES presents similarly to food-protein induced enterocolitis syndrome (FPIES) and is characterized by severe delayed-onset emesis within 2–4 h after drug administration. DIES is considered a cell-mediated disorder, though specific pathogenic mechanisms remain unclear. It is often misdiagnosed, and likely under recognized in children.

**Case Presentation:** We present the case of a 10-year-old female with a history of delayed-onset projectile emesis at 3 years of age following an oral amoxicillin challenge for suspected penicillin allergy. Under the supervision of a pediatric allergist, she underwent negative epicutaneous and intradermal testing to penicillin at age 10, followed by an observed oral drug challenge to amoxicillin (125 mg). She had one episode of emesis 4 h after challenge testing. A repeat challenge was performed in clinic 6 months later, and again she had emesis, nausea and pallor 2 h after the amoxicillin dose. She was treated with a single dose of intramuscular ondansetron (0.15 mg/kg). She had one episode of diarrhea 4 h after challenge onset. She remained hemodynamically stable, had no associated extra-intestinal symptoms, and was able to maintain enteral hydration throughout. She was observed until 5.5 h post challenge and discharged home thereafter in good condition.

**Conclusions:** This case report adds to an expanding knowledge and recognition for DIES. History of delayed emesis after three oral penicillin challenges is highly suggestive for a diagnosis of DIES by amoxicillin. Similarly to FPIES, drug-induced enterocolitis syndrome may present with severe and potentially life-threatening disease with systemic shock. Further descriptions of its clinical course are required to increase disease understanding and encourage prompt recognition and treatment.

## 63 Case series and practice approach to atypical food protein-induced enterocolitis syndrome (FPIES)

### Vivian G. Szeto^1,2^, Harold Kim^1,2^

#### ^1^McMaster University, Hamilton, ON; ^2^Western University, London, ON

##### Correspondence: Vivian G. Szeto

*Allergy, Asthma & Clinical Immunology* 2025, **21(Suppl 1)**:63

**Background:** Food protein-induced enterocolitis syndrome (FPIES) is a non-IgE mediated food allergy. Symptoms of protracted vomiting are often delayed 1–4 h after ingesting the trigger food. The most common trigger foods include cow’s milk, soy, oat, and rice. Treatment includes avoiding the trigger food and retesting with an oral food challenge (OFC) 12–18 months after last exposure. In atypical FPIES, these patients present initially as acute FPIES, but will over time develop serum specific IgE to their trigger foods.

The aim is to to broaden the understanding of atypical FPIES and provide a practice approach to reduce the risk of developing atypical FPIES. We present a case series of patients with atypical FPIES. Parental consent was obtained for publication of these cases.

Case Presentation: 

*Case 1:* 24-month-old female with atypical FPIES to egg. Skin prick testing was 4 mm and serum specific IgE was 2.77 (normal < 0.35). Treated with our previously published modified OFC protocol for chronic FPIES. Patient tolerates 50 g of egg powder which is equivalent to 1 egg.

*Case 2:* 24-month-old-male with atypical FPIES to milk. Skin prick test was 16 mm and serum specific IgE was 0.91 (normal < 0.35). Treated with a proposed blended oral immunotherapy (OIT) protocol with our previously published modified OFC protocol for chronic FPIES. Patient tolerates 30 mL of milk.

*Case 3:* 48-month-old male atypical FPIEs to egg. Skin prick test was 3 mm with serum specific IgE 0.41 (normal < 0.35). Treated with previously published modified OFC protocol for chronic FPIES. Patient tolerates 40 g of egg powder and continues to updose.

Conclusions:

1. Consider skin prick testing and serum specific IgE levels in individuals who present with FPIES.
2. Propose the use of our blended OIT protocol with the modified OFC to treat atypical milk FPIES and higher risk FPIES to help outgrow and desensitize patients with allergic FPIES.

## 64 Genetic findings complicating therapeutic interventions for a pediatric patient with chronic granulomatous disease

### Kristine Jeganathan, Anne Pham-Huy

#### Children’s Hospital of Eastern Ontario, Ottawa, ON

##### **Correspondence:** Kristine Jeganathan

*Allergy, Asthma & Clinical Immunology* 2025, **21(Suppl 1)**:64

**Background:** Chronic Granulomatous Disease (CGD) is a rare, primary immunodeficiency disorder characterized by the inability of phagocytes to produce reactive oxygen species. The most common type of CGD is inherited in an X-linked recessive manner due to a mutation in the CYBB gene located in the Xp21.1 region of the X chromosome. Rarely, CGD can be caused by a deletion in the X chromosome with larger deletions involving neighboring genes, including DMD (Duchenne muscular dystrophy) and XK (McLeod Syndrome). These rare cases significantly affect patient prognosis.

**Case Presentation:** A 2-year-old boy presented to hospital with hepatomegaly, constipation, and vomiting. Imaging revealed a significant liver abscess, which cultured *Serratia marcescens*, prompting further immunological investigations. Neutrophil function tests demonstrated impaired oxidative burst, consistent with CGD. Subsequent genetic testing revealed a pathogenic large deletion at Xp21.1-p11.3, encompassing multiple OMIM Morbid genes, including DMD, Cilia- and flagella-associated protein (CFAP47 infertility), and XK (McLeod syndrome). The identification of this deletion provided a broader context for the patient’s phenotype and future health concerns.

Given the diagnosis of CGD, he was evaluated for curative therapy, hematopoietic stem cell transplantation (HSCT). He is followed by immunology, palliative care, and neurology. Current treatment planning discusses the benefits and risks of HSCT, given the prognosis of multiple life-limiting conditions. Untreated CGD can lead to severe life-threatening infections, while the risk for HSCT includes pulmonary fibrosis from chemotherapy, toxicities from the conditioning regimen, and graft vs host disease.

**Conclusions:** This case highlights the importance of comprehensive genetic testing for patients with CGD, to provide crucial information for immediate and long-term management. Large genetic deletions in patients with X-linked CGD are rare, but can complicate prognosis, and therapeutic planning. Multidisciplinary medical teams, including palliative care and medical ethics, should collaborate with the families to understand various perspectives and determine the best treatment plan for these patients.

## Food Allergy/Anaphylaxis

## 65 Strategies to expedite access to consultation for pediatric food allergy

### Kristine Jeganathan^1^, Matthew Griffin^2^, Deborah Leblanc^2^, Adele Nylen^2^, Edmond Chan^3,4^, Stephanie Erdle^3,4^, Scott Cameron^2,3^, Victoria Cook^2,3^

#### ^1^Department of Pediatrics, Children’s Hospital of Eastern Ontario, Ottawa, ON; ^2^Community Allergy Clinic, Island Health Authority, Victoria, BC; ^3^Division of Allergy, Department of Pediatrics, University of British Columbia, Vancouver, BC; ^4^British Columbia Children’s Hospital Research Institute, Vancouver, BC

##### **Correspondence:** Kristine Jeganathan

*Allergy, Asthma & Clinical Immunology* 2025, **21(Suppl 1)**:65

**Background:** Long wait times for patients < 2 years old increase the risk of delayed introduction of priority allergens, and postpone initiation of oral immunotherapy (OIT), which is most beneficial when started early [1–4]. Barriers to increased throughput in this population include lengthy initial visits and need for multiple follow-up appointments [5–6].

**Methods**: We evaluated a plan-do-study-act (PDSA) cycle targeting pre-assessment patient education. We developed educational materials covering epinephrine use, priority allergen introduction, and ladder therapy (https://www.allergyvic.com/qi-project). Information packages tailored to referral concerns were emailed to caregivers of patients < 2 years old referred for food allergy. Caregivers were instructed to implement recommendations while awaiting assessment, with email support. Process measures included visit length and number of follow-up visits prevented. Caregiver surveys assessed satisfaction and concordance between the information we provided and counseling by referring providers. Administrative burden was tracked through weekly office meeting discussions.

**Results:** Between April 15, 2024 and May 15, 2024, 50 information packages were sent. After reviewing packages, 4 patients felt further assessment was not required. Of 16 patients who underwent assessment in May 2024, 7 (44%) received and reviewed the information; post-intervention, average visit length was 44 min, reduced from pre-intervention average (65 min). Use of the intervention prevented 1 follow-up visit per patient. Caregiver surveys (N = 6) identified that contradictory advice was given by referring providers (unnecessary food avoidance), all caregivers found the received information packages helpful. Approximately 30% of referrals lacked an email address, increasing administrative burden.

**Conclusions:** Patient education prior to assessment can increase capacity through reduced visit length and reduced need for follow-up. Uptake of information is a barrier, with > 50% of patients not reading the provided material. We are therefore evaluating the impact of simplified email communication and implemented reminders. Referring providers often recommend avoidance of priority allergens, and our next PDSA cycle involves referring provider education.

**References:**

1. Cook VE, Yeo J, Mill C, Hildebrand K, Portales-Casamar E, Chan ES. Many children referred to a tertiary care pediatric allergy clinic do not have an allergy. *J Allergy Clin Immunol.* 2017;139(2).
2. Vickery BP, Berglund JP, Burk CM, Fine JP, Kim EH, Kim JI, Burks AW. Early oral immunotherapy in peanut-allergic preschool children is safe and highly effective. *J Allergy Clin Immunol.* 2017;139(1):173–181.
3. Hsu E, Soller L, Abrams EM, Protudjer JLP, Mill C, Chan ES. Oral food challenge implementation: the first mixed-methods study exploring barriers and solutions. *J Allergy Clin Immunol Pract*. 2020;8(1):149–156.
4. Abrams EM, Watson W, Vander Leek TK, Atkinson A, Primeau MN, Francoeur MJ, Chan ES. Dietary exposures and allergy prevention in high-risk infants. *Allergy Asthma Clin Immunol.* 2022;18(1):36.
5. Hiscock H, Perera P, Danchin MH, Sung V, Lee K, Stival C, Tang ML. Improving timely access to food allergy care: a pragmatic controlled trial. *Allergy*. 2020;75(6).
6. Cheon J, Cho CM, Kim HJ, Kim DH. Effectiveness of educational interventions for quality of life of parents and children with food allergy: a systematic review. *Medicine*. 2022;101(36).

## 66 SORT: safety of OIT in a randomized trial for peanut and milk

### Adnan Al Ali^4^, Danbing Ke^4^, Duncan Lejtenyi^4^, Liane Beaudette^4^, Eyal Grunebaum^2^, Julia Upton^1^, Edmond S. Chan^3^, Xun Zhang^4^, Casey Cohen^4^, Abigail Brodovitch^4^, Christine McCusker^4^, Bruce Mazer^4^, Moshe Ben-Shoshan^4^

#### ^1^Clinical Immunology and Allergy, Dept. of Pediatrics, The Hospital For Sick Children, Toronto, ON; ^2^Division of Immunology and Allergy, Department of Pediatrics, The Hospital for Sick Children, Department of Paediatrics, University of Toronto, Toronto, ON, Canada., Toronto, ON; ^3^Division of Allergy, Department of Pediatrics, BC Children’s Hospital, University of British Columbia, Vancouver, British Columbia, Canada, Canada, BC; ^4^Division of Allergy and Clinical Immunology, Department of Pediatrics, Montreal Children’s Hospital, McGill University Health Centre, Montreal, QC, Canada., Montréal, QC

##### **Correspondence:** Adnan Al Ali

*Allergy, Asthma & Clinical Immunology* 2025, **21(Suppl 1)**:66

**Background:** Oral immunotherapy (OIT) has emerged as a promising approach treating food allergy. However, concerns regarding safety and the lack of standardized protocols have impeded the widespread adoption of OIT.

**Methods**: We compared three OIT protocols for milk and peanut allergy. Protocols A and B followed a dose escalation with a target dose of 8000 mg and 2000 mg of milk protein and 300 mg and 30 mg of peanut protein respectively. Protocol C modified the protocol A by incorporating a segment of up-dosing with processed forms of milk (like muffin containing 400 mg protein per muffin), and peanut (as puff containing 80 mg protein per puff) Allergic reactions were classified as local reactions (limited to oral or throat tingling), allergic reactions (involving one organ system) and anaphylactic reactions, (2 or more organ system(s)/hypotension). The association between reaction severity and factors like food type, baseline characteristics, etc. were determined via the mixed effects model.

**Results:** There were 20 children recruited for milk and 19 for peanuts in total to date. The median age was 9.9 years (IQR: 3.8). Processed forms were associated with more local reactions (aOR: 2.02, 95% CI: 1.01–4.07, p = 0.048). However, processed forms significantly reduced all allergic reactions (aOR: 0.39, 95% CI: 0.22–0.67, p = 0.001). The maximal tolerated dose at baseline was associated with an increase in total reactions (aOR: 1.31, 95% CI: 1.01–1.69, p = 0.043).

**Conclusions:** Processed forms seem to offer a safe approach to OIT. The sample size was likely insufficient to detect any association with anaphylactic or more severe reactions due to their low incidence during OIT. Further research with larger sample sizes is necessary to compare appropriately between the three protocols.

**References:**

- 1. Panel NS. Guidelines for the diagnosis and management of food allergy in the United States: report of the NIAID-sponsored expert panel. Journal of Allergy and Clinical Immunology. 2010 Dec 1;126(6):S1-58. doi:10.1016/j.jaci.2010.10.007

## 67 Allergen-specific antibodies and the development of allergies: the CHILD cohort study

### Akash Kothari^1,2^, Myrtha E. Reyna^1,2^, Kristen Ericka San Diego^2^, Aimee Dubeau^2^, Lisa Hung^1, 2^, Carmen Li^1,2^, Julia E. Upton^2^, Xiaojun Yin^2^, Piush Mandhane^3^, Elinor Simons^4^, Stuart Turvey^5^, Meghan B. Azad^4^, Padmaja Subbarao^2^, Theo J. Moraes^1,2^, Thomas Eiwegger^1,2,6^

#### ^1^University of Toronto, Toronto, ON; ^2^Hospital for Sick Children, Toronto, ON; ^3^University of Alberta, Edmonton, AB; ^4^University of Manitoba, Winnipeg, MB; ^5^BC Children’s Hospital Research Institute, Vancouver, BC; ^6^Karl Landsteiner University of Health Sciences and University Hospital St. Pölten, Krems, Austria

##### **Correspondence:** Akash Kothari

*Allergy, Asthma & Clinical Immunology* 2025, **21(Suppl 1)**:67

**Background:** Maternal antibody transfer in pregnancy and breastmilk is implicated in the development of allergen-specific immune tolerance in the child. However, prospective human studies investigating the effect of maternal allergen-specific IgG (sIgG) transfer on allergic diseases in offspring are limited.

**Methods**: Maternal sIgG and sIgE were measured in serum at 18 weeks’ gestation (n = 479) and sIgE of the child was measured in serum/plasma at 1 year in a subset of the CHILD birth cohort (n = 475) using an allergen macro-array. In addition, breastmilk sIgG was also measured where available at 3 months postpartum (n = 279). Log ratios for each allergen were calculated if > 10 children were sensitized at 1 year of age (sIgE > 0.3 kUA/L). Hierarchical clustering of serum and breastmilk sIgG was used to analyze the impact on atopic outcomes at 1 year of age via multivariable logistic regression models.

**Results:** Mean maternal serum sIgG was not significantly different in IgE-sensitized children versus non-sensitized at 1 year of age for all but one allergen. Breastmilk/serum sIgG ratios were significantly lower among IgE-sensitized children versus non-sensitized for cashew (*p* = 0.018) and Ara h 2 (*p* = 0.007), and higher for Gal d 2 (*p* = 0.005) and almond (*p* = 0.019). Regression models suggest maternal serum sIgG, but not breastmilk sIgG as a significant risk factor for allergic sensitization (skin prick test or sIgE) at 1 year of age.

**Conclusions:** Relative sIgG in breastmilk versus serum may affect IgE sensitization in offspring. A higher abundance of maternal serum sIgG may reflect a higher propensity of allergic sensitization in the child. Breastmilk sIgG alone is not associated with a higher risk of IgE-sensitization at 1 year of age.

## 68 An observational descriptive study of fatal anaphylaxis in Canada: – a canadian registry for anaphylaxis fatalities (CRAFT)

### Alexandre Ton That^1^, Joseph Najem^1^, Khaled M Hazzazi^10^, Jennifer L. Protudjer^2^, Elissa Abrams^11^, Waleed Alqurashi^3^, Edmond Chan^5^, Anna K. Ellis^4^, Jennifer Gerdts^6^, Timothy Vander Leek^7^, Rod Lim^8^, Paul-Andre Perron^9^, Moshe Ben-Shoshan^1^

#### ^1^Faculty of Medicine, McGill University, Montreal, QC; ^2^Department of Pediatrics and Child Health, Children’s Hospital Research Institute of Manitoba, Winnipeg, MB; ^3^Department of Pediatrics and Emergency Medicine, University of Ottawa, Ottawa, ON; ^4^Kingston Health Sciences Centre, Kingston, ON; ^5^Division of Allergy, Department of Pediatrics, BC Children’s Hospital, University of British Columbia, Vancouver, BC; ^6^Food Allergy Canada, Toronto, ON; ^7^Department of Pediatrics, Stollery Children’s Hospital, University of Alberta, Edmonton, Alberta, Canada—Department of Pediatrics, University of British Columbia, Edmonton, AB; ^8^Division of Pediatric Emergency Medicine, Department of Pediatrics, Children’s Hospital at London Health Science Centre, London, ON; ^9^Bureau du coroner, Quebec, QC; ^10^King Faisal Specialist Hospital and Research Centre, Madinah, Saudi Arabia; ^11^Public Health Agency of Canada- Department of Pediatrics, Section of Allergy and Clinical Immunology, University of Manitoba-Department of Pediatrics, Division of Allergy and Immunology, University of British Columbia- Allergy Section, Canadian Pediatric Society- Canadian Society of Allergy and Clinical Immunology, Winnipeg, MB

##### **Correspondence:** Alexandre Ton That

*Allergy, Asthma & Clinical Immunology* 2025, **21(Suppl 1)**:68

**Background:** Anaphylaxis is a severe allergic reaction, which in rare occasions, is fatal. Yet, Canadian data on anaphylaxis fatalities are sparse. Herein, we aimed to describe causes, associated factors and management of fatal anaphylaxis in Canada.

**Methods**: The Canadian Registry for Anaphylaxis Fatalities (CRAFT) is the first Canadian registry to collect data through a standardized questionnaire including sociodemographic, triggers, clinical presentation, comorbidities and management. Data were collected through coroners’ reports, lay allergy associations, case reports from social media and medical literature. Probable/unlikely anaphylaxis was defined as anaphylaxis by a board-certified allergist meeting the National Institute of Allergy and Infection Diseases criteria. Descriptive statistics were used to determine prevalence amongst the data, including, age, location of anaphylaxis, allergen exposure, epinephrine use, comorbidities present and presence of alcohol/cannabis. The study was approved by McGill’s Research Ethics Board.

**Results:** Cases were obtained from Quebec (n = 16), Ontario (n = 29), Alberta (n = 2) and Saskatchewan (n = 5) between January, 2020-May, 2024. Overall, 80.4% of cases were probable anaphylaxis. Median age of the 41 probable cases was 45.0 years (interquartile range = 31.0–60.0), 53.6% were males. Among those, 36.6% cases occurred at home, 26.8% outdoors, 17.1% in healthcare settings, 2.4% at restaurants, 17.1% in another/unknown location. Allergen exposure occurred via insect stings (53.6%), ingestion of food (26.8%), intravenous (12.1%), and inhalation (7.3%), but with noted geographical differences. Insect stings were the main trigger across all provinces. Epinephrine was used in 58.5% of cases. Comorbidities including asthma/cardiac/other conditions were reported in 7.3%, 17.0%, and 44.3%, and 53.6% had a reported known allergy to the trigger. Alcohol/cannabis were present in 9.7% and 4.8% respectively.

**Conclusions:** Insect stings accounted for more anaphylaxis fatalities in Canada than food, contrasting with previous preliminary data. Most cases had comorbid conditions (68.6%). Epinephrine was not used in almost half of cases, highlighting the need for better guidelines, policies and education programs.

## 69 Peanut sensitivity goals: low vs standard dose peanut oral immunotherapy skin prick test adventures

### Diana Toscano Rivero^1^, Casey G. Cohen^1^, Wei Zhao^1^, Eisha A. Ahmed^1^, Danbing Ke^2^, Liane Beaudette^2^, Duncan Lejtenyi^2^, Thomas Eiwegger^3^, Moshe Ben-Shoshan^2^, Julia Upton^3^, Bruce D. Mazer^1,2^

#### ^1^Research Institute of the McGill University Health Centre, Department of Pediatrics and Experimental Medicine, Faculty of Medicine, McGill University, Montreal, Canada, Montreal, QC; ^2^Division of Allergy and Clinical Immunology, Department of Pediatrics, Montreal Children’s Hospital, Montreal, Quebec, Canada, Montreal, QC; ^3^Division of Immunology and Allergy, Department of Pediatrics, Hospital for Sick Children, University of Toronto, Toronto, Ontario, Canada., Toronto, ON

##### **Correspondence:** Diana Toscano Rivero

*Allergy, Asthma & Clinical Immunology* 2025, **21(Suppl 1)**:69

**Background:** Peanut oral immunotherapy (OIT) aims to decrease allergic reactions by gradually exposing individuals to escalating doses of peanuts until a maintenance dose of 300 mg is reached. However, dose-related adverse reactions frequently hinder this process, resulting in participant withdrawals. Data on clinical and immunological parameters associated with maintenance doses below 300 mg is limited.

**Methods**: A subgroup of 22 peanut-allergic subjects aged 7 to 21 years, who were part of a larger peanut OIT trial of low dose (30 mg, N = 10) or standard maintenance dose (300 mg, N = 12) were evaluated. The median participant age was 17 years for the 30 mg group (50% females) and 16 years for the 300 mg group (58% females). The analysis focused on median wheal sizes from skin prick tests (SPT) and median Ara h 2-specific IgG4 and IgE levels measured before and after one year of dose escalation.

**Results:** After a median escalation phase of 16 months, **wheal size significantly decreased** from a baseline of 9 mm (minimum 4 – maximum 20) to 5 mm (1 – 13) in the 30 mg group (p = 0.01), and **Ara h 2-specific-IgG4 significantly increased** from 34ug/mL (19 – 276) to 291ug/mL (26 – 1,230; p = 0.006). Similarly, in the 300 mg group, **wheal size significantly decreased** from a baseline of 8.5 mm (3 – 21) to 5 mm (1 – 12; p = 0.05), and **Ara h 2-specific-IgG4 significantly increased** from 91ug/mL (15 – 365) to 363ug/mL (8.2 – 11,375; p = 0.02). Ara h 2-specific-IgE did not change significantly in either group, from 243 ng/mL (0.75– 5,310) to 419 ng/mL (0.97 – 7,904) in the 30 mg group and from 498 ng/mL (2.76 – 3,198) to 374 ng/mL (1.6 – 5,155) in the 300 mg group (p > 0.05).

**Conclusions:** The results suggest that a 30 mg maintenance dose of peanut OIT induces comparable immunological and clinical responses similar to 300 mg in peanut-allergic individuals. Larger-scale studies with expanded sample sizes are warranted.

## 70 Evaluating the efficacy of a novel oral food challenge protocol for pediatric FPIES patients

### Emily G. Morris^1^, Peter W. Huan^1^, Jennifer L. Protudjer^2,3,4^, Harold Kim^5,6^

#### ^1^Department of Medicine, Western University, London, ON, ^2^Department of Pediatrics and Child Health, University of Manitoba, Winnipeg, MB, ^3^Children’s Hospital Research Institute of Manitoba, Winnipeg, MB, ^4^Karolinska Institutet, Stockholm, Sweden, ^5^Division of Clinical Immunology and Allergy, Department of Medicine, Western University, London, ON, ^6^Department of Medicine, McMaster University, Hamilton, ON

##### **Correspondence:** Emily G. Morris^1^, Peter W. Huan

*Allergy, Asthma & Clinical Immunology* 2025, **21(Suppl 1)**:70

**Background:** Oral food challenges (OFCs) are considered the gold standard for diagnosis of food protein-induced enterocolitis syndrome (FPIES). A modified approach to OFCs involves smaller, gradually increased doses to mitigate the risk of severe reactions. We aimed to measure the successful completion of this OFC protocol.

**Methods**: In a retrospective chart review, 41 patients < 18 years, who had at least 1 episode of acute FPIES between 2015–2023 were identified using an allergy clinic database. The patients underwent OFCs with home up-dosing every 2–4 weeks. Steps included 1%, 2%, 5%, 10%, 20%, 30%, 40%, 60%, 80%, and 100% of the final serving amount. The primary outcome measured successful completion, defined by absence of severe reactions during the OFC protocol and 1 year after. Data were analysed using logistic regression and reported as odds ratios (OR) and 95 percent confidence intervals (95%CI). Results were adjusted for multiple allergic comorbidities, age of FPIES onset, and biological sex.

**Results:** 41 patients began the OFC protocol, of whom 32 (78.05%) completed the protocol without significant reactions. Of the 9 (21.95%) who did not complete the protocol, 4 (44.4%) paused due to reactions, and 5 (55.6%) paused due to non-FPIES symptoms. No significant associations were identified between OFC completion and severity of symptoms (OR 1.20; 95%CI 0.26—5.45; p = 0.81); OFC completion and age at onset of symptoms (OR 0.98; 95%CI 0.92—1.04; p = 0.45); or OFC completion and age of starting OFC (fully adjusted OR 0.99; 95%CI 0.95—1.02; p = 0.53). Compared to foods other than milk/dairy, those who reacted to milk tended to be likely to not complete (OR 4.86; 95%CI 0.75—31.56; p = 0.09).

**Conclusions:** This study supports the potential for a gradual approach to OFCs in FPIES, evidenced by a high completion rate. Further research is needed to characterize safe approaches to OFC in FPIES.

## 71 The impact of artificial intelligence (AI) on parent education in oral immunotherapy (OIT)

### Jana Abi-Rafeh^1,2^, Diana Toscano-Rivero^1,2^, Bruce D. Mazer^1,2,3^

#### ^1^Meakins-Christie Laboratories, Montreal, QC; ^2^Translational Research in Respiratory Diseases Program, McGill University Health Centre Research Institute, Montreal, QC; ^3^Department of Pediatrics, Division of Allergy/Immunology/Dermatology, McGill University Health Center, Montreal, QC

##### **Correspondence:** Jana Abi-Rafeh

*Allergy, Asthma & Clinical Immunology* 2025, **21(Suppl 1)**:71

**Background:** There is a lack of national consensus on the management and practices of Oral Immunotherapy (OIT), including but not limited to the definition of desensitization and protocol guidelines. With a lack of standardized management guidelines, the impact of OIT on families is substantial. Parents have concerns regarding OIT practices, meal preparation, and social activities involving food. As such, it is imperative to continue assessing different means of education and accessible resources for parents with children undergoing OIT. ChatGPT is part of a new generation of AI technology which is now widely recognized for its ability to formulate human-like conversations and address a wide range of topics. With over 180-million users worldwide, studies have demonstrated the superior ability of ChatGPT to respond to a wide range patients’ health care questions. Currently, there knowledge gap in addressing the impact of ChatGPT on OIT education.

**Methods**: 14 common questions asked by parents regarding OIT were compiled and typed into ChatGPT-3.5. Chatbot answers were copied verbatim and assessed by allergy/immunology staff, residents and nurses from across Montreal, Toronto and Texas. We analyzed the responses from participants, who rated the ChatGPT answers using a 10-point Likert Scale.

**Results:** Questions are divided into three categories: basic, advanced and medical, with a preliminary average ranking of 8.14, 7.86 and 7.40, for each category, respectively. Moreover, when asked if participants would be open to using ChatGPT as a means of patient education, 45% said very likely, 45% said likely while 9% said somewhat unlikely. Further analysis discussing response readability, reproducibility, and understandability is subject to dataset completion.

**Conclusions:** Within this study, ChatGPT showed a great ability to respond to patient questions and was ‘recommended’ by over 90% of participants.

## 72 Socioeconomic status is associated with food allergy-anxiety in caregivers

### Michael A. Golding^1,2^, Lianne Soller^3^, Jennifer Y. Tong^3^, Edmond S. Chan^3,4^, Jennifer L. Protudjer^1,2,5^

#### ^1^Children’s Hospital Research Institute of Manitoba, Winnipeg, MB; ^2^University of Manitoba, Winnipeg, MB; ^3^University of British Columbia, Vancouver, BC; ^4^British Columbia Children’s Hospital Research Institute, Vancouver, BC; ^5^Karolinska Institutet, Stockholm, Sweden

##### **Correspondence:** Michael A. Golding

*Allergy, Asthma & Clinical Immunology* 2025, **21(Suppl 1)**:72

**Background:** Studies suggest that anxiety disorders are more common among individuals with lower socioeconomic status (SES), but the link between SES and food allergy anxiety in caregivers is not well studied. This study aimed to explore this relationship in caregivers of children initiating immunotherapy for food allergy.

**Methods**: This study analyzed data from the Food Allergy Immunotherapy (FAIT) Registry. This database is comprised of data collected from parents of children undergoing immunotherapy and includes information on the child’s clinical history, family demographics, and parental food allergy anxiety. Anxiety was quantified using IMPAACT, which is a self-report questionnaire that yields a total anxiety score and four subscale scores (i.e., cognitive, anxiety impact, behavioural avoidance, & child/parental coping). For the current study, we investigated the relationship between family income, caregiver education, and baseline level of caregiver food allergy anxiety (i.e., collected prior to immunotherapy) using a series of multiple regression analyses. This study was approved by the UBC Research Ethics Board.

**Results:** The sample included 51 parents, mostly of European (53%) or East Asian (25%) ethnicity. Most participants had at least an undergraduate degree (76%), and the majority had family incomes over $150,000 annually (63%). Multiple regression analyses showed significant relationships between household income and caregiver food allergy anxiety. Households earning over $200,000 annually reported significantly lower total anxiety levels compared to those earning less than $65,000 (b = -47.18, p = 0.03). Similarly, households earning $100,001-$150,000 (b = -16.96, p = 0.03), $150,001-$200,000 (b = -15.92, p = 0.03), and $200,000 + (b = -19.83, p = 0.01) reported significantly lower levels of behavioral avoidance than those earning less than $65,000 annually.

**Conclusions:** Findings suggests that lower levels of income are associated with higher levels of food allergy anxiety in caregivers. As this is a new finding, more research is needed to better understand the cause of these income-related differences.

## 73 Examining ethnicity differences as a factor of peanut oral immunotherapy

### Peter W. Huan^1^, Emily G. Morris^1^, Jennifer L. Protudjer^2,3,4^, Samira Jeimy^5^, Harold Kim^5,6^

#### ^1^Department of Medicine, Western University, London, ON; ^2^Department of Pediatrics and Child Health, University of Manitoba, Winnipeg, MB; ^3^Children’s Hospital Research Institute of Manitoba, Winnipeg, MB; ^4^Karolinska Institutet, Stockholm, Sweden; ^5^Division of Clinical Immunology and Allergy, Western University, London, ON; ^6^Faculty of Medicine, McMaster University, Hamilton, ON

##### **Correspondence:** Peter W. Huan, Emily G. Morris

*Allergy, Asthma & Clinical Immunology* 2025, **21(Suppl 1)**:73

**Background:** Peanut Oral Immunotherapy (pOIT) is effective for facilitating a state of desensitization or sustained unresponsiveness for patients living with peanut allergies. How ethnicity can affect the outcomes of pOIT is yet to be studied. We aimed to describe differences in rates of pOIT completion and side effects with consideration to patient ethnicity.

**Methods**: In a retrospective chart review, patients were identified as either completed or currently undergoing pOIT at an outpatient allergy clinic from 2017–2024. The primary outcome measured successful completion pOIT protocol, defined by absence of severe reactions during the buildup phase and 1 year after. Secondary outcomes included identifying reasons for discontinuing the protocol. Data were described, and analyzed using logistic regression, reported as odds ratios (OR) and 95 percent confidence intervals (95%CI). Results were adjusted for multiple allergic comorbidities, age at first reaction, biological sex, family history of food allergy, and other priority food allergies. This study was approved by the Western University Health Sciences Research Ethics Board (HSREB number 114291).

**Results:** Overall, 260 children, including 175 (67.3%) males, underwent the pOIT protocol, of whom 177 (68.1%) completed treatment. The mean age at the start of pOIT was 7.5 ± 5.1 years. Most children were White (72.3%), had multiple allergic comorbidities (78.1%) and had a family history of food allergy (73.1%). Compared to White children, non-White children had fewer pOIT completions (OR 0.28; 95%CI 0.08–1.00; p = 0.05); comparable odds of side effects as a reason for stopping pOIT (OR 1.22; 95%CI 0.36–4.18; p = 0.74) and significantly greater odds of stopping pOIT because of parental anxiety (OR 5.55; 95%CI 1.43–21.54; p = 0.01).

**Conclusions:** Non-White children had fewer pOIT completions compared to While children and were more likely to stop because of parental anxiety. Efforts to understand and address potential disparities regarding ethnicity and the completion of pOIT are warranted.

## 74 Real-world effectiveness analysis of preschool tree nut oral immunotherapy: An update with more patients

### Simonne L. Horwitz^1^, Lianne Soller^2^, Victoria E. Cook^2,3^, Scott B. Cameron^2,3^, Joanne Yeung^2,4^, Sandeep Kapur^5,6^, Mary McHenry^5,6^, Edmond S. Chan^2^, Raymond Mak^2^, Gregory A. Rex^5,6^, Tiffany Wong^2^, Stephanie C. Erdle^2^

#### ^1^Department of Pediatrics, The Hospital for Sick Children, University of Toronto, Toronto, ON; ^2^Division of Allergy, Department of Pediatrics, University of British Columbia, Vancouver, BC; ^3^Community Allergy Clinic, Victoria, BC; ^4^Vancouver Kids Allergy, Vancouver, BC; ^5^Division of Allergy, Department of Pediatrics, Dalhousie University/IWK Health Centre, Halifax, NS; ^6^Halifax Allergy and Asthma Associates, Halifax, NS

##### **Correspondence:** Simonne L. Horwitz

*Allergy, Asthma & Clinical Immunology* 2025, **21(Suppl 1)**:74

**Background:** We previously described the safety of preschool tree nut oral immunotherapy (TN-OIT) in a real-world setting and preliminary results of the real-world effectiveness of preschool TN-OIT after at least one year of daily maintenance TN-OIT in 47 patients. This is an update to previously presented data, with more patients.

**Methods**: Through a Canada-wide study, preschool-age children with 1) objective reactions to tree nut (TN) and positive skin prick tests or sIgE levels ≥ 0.35kU/L or 2) sIgE levels ≥ 5kU/L, received a follow-up oral food challenge (OFC) to cumulative 4000 mg TN protein (or a smaller amount deemed age-appropriate) after at least one year of 300 mg maintenance TN-OIT. Each patient completed TN-OIT and follow-up OFC to ≥ one TN (cashew/pistachio, walnut/pecan, hazelnut, almond, or macadamia nut); some patients completed TN-OIT to multiple TNs simultaneously. Desensitization effectiveness was defined as proportion of patients with successful follow-up OFC. Symptoms were classified using the modified World Allergy Organization Subcutaneous Immunotherapy Systemic Reaction Grading System (1, mildest; 5, fatal).

**Results:** 136 patients completed TN-OIT and underwent follow-up OFCs to at least one TN. 106/136 (77.9%) had a successful OFC to 4000 mg protein; an additional 18/136 (13.2%) had a successful OFC to a lesser, age-appropriate amount (on average 3200 mg protein), for a 91.2% (124/136) cumulative pass rate. 12/136 (8.8%) patients had a grade 1 or 2 reaction; there were no grade 3–5 reactions. 3/136 (2.2%) patients received epinephrine. Overall, 132/136 (97.1%) patients tolerated ≥ 1000 mg protein.

**Conclusions:** Our data in a greater number of patients demonstrates that real-world preschool TN-OIT is effective, with comparable outcomes to previously published peanut and sesame OIT data. Symptoms were mild in those who reacted during the follow-up OFC. Nearly all patients had their threshold increased sufficiently to protect against accidental exposures. TN-OIT should be considered for preschool-age children as an alternative to avoidance.

## 75 Milk protein-specific IgG4 is associated with adverse reaction severity during oral immunotherapy

### Wei Zhao^1^, Danbing Ke^2^, Casey Cohen^1^, Christine McCusker^2^, Julia E. Upton^4^, Edmond S. Chan^3^, Ann E. Clarke^6^, Philippe Bégin^5^, Eyal Grunebaum^4^, Moshe Ben-Shoshan^2^, Bruce D. Mazer^2^

#### ^1^The Research Institute of the McGill University Health Centre and the Department of Pediatrics, Faculty of Medicine, McGill University, Montreal, QC; ^2^Division of Allergy and Clinical Immunology, Department of Pediatrics, Montreal Children’s Hospital, McGill University Health Centre, Montreal, QC; ^3^Division of Allergy and Immunology, Department of Pediatrics, BC Children’s Hospital, University of British Columbia, Vancouver, BC; ^4^Division of Immunology and Allergy, Department of Pediatrics, Hospital for Sick Children, University of Toronto, Toronto, ON; ^5^Department of Pediatrics, Service of Allergy and Clinical Immunology, Centre Hospitalier Universitaire Sainte-Justine, Montreal, QC; ^6^Division of Rheumatology, Department of Medicine, University of Calgary, Calgary, AB

##### **Correspondence:** Wei Zhao

*Allergy, Asthma & Clinical Immunology* 2025, **21(Suppl 1)**:75

**Background:** Cow’s milk allergy is one of the leading causes of anaphylaxis in children. Oral immunotherapy (OIT) is an effective treatment option for food allergies, but its safety remains to be improved. Identifying any biomarkers linked to the risk of adverse reactions is useful in improving the success rate and safety of milk OIT. Since adverse reactions are most often observed during the escalation phase, which ends when participants reach 200 ml of cow’s milk, we aim to investigate the association of cow’s milk protein (CMP)-specific immunoglobulin G4 (IgG4) to adverse reactions.

**Methods**: We documented adverse reactions and collected serum samples during the escalation phase of our milk OIT. Levels of IgG4 specific for the main CMPs: α-Lactalbumin (ALA), β-Lactoglobulin (BLG), and casein, were measured using enzyme-linked immunosorbent assay (ELISA). Adverse reactions were graded as described in De Schrvyer et al*.* [1]. The association between reaction severity and factors including IgG4 levels was determined using the Poisson regression.

**Results:** Our study included 76 OIT participants (42% female and 58% male) with an average age of 11. Males were associated with a decrease in the number of moderate anaphylaxis reactions (MAR) (incidence rate ratio (IRR): 0.44, 95% confidence interval (CI) 0.30–0.65, P < 0.001). Reaching 200 ml was associated with a decreased incidence of MAR (IRR 0.52, CI 0.36–0.77, P = 0.001). Increased ALA-IgG4 and CAS-IgG4 were associated with decreased incidence of oral symptoms (IRR 0.91 for both, CI 0.88–0.94, 0.88–0.93 respectively, P < 0.001). Increased CAS-IgG4 was associated with a decreased incidence of MAR (IRR 0.88, CI 0.85–0.91, P < 0.001).

**Conclusions:** Our results indicate that sex and increased CAS-specific IgG4 are associated with decreased MAR during the escalation phase of milk OIT.

**References:**

1. De Schryver S, Mazer B, Clarke AE, St. Pierre Y, Lejtenyi D, Langlois A, et al. Adverse Events in Oral Immunotherapy for the Desensitization of Cow’s Milk Allergy in Children: A Randomized Controlled Trial. J Allergy Clin Immunol Pract. 2019 Jul 1;7(6):1912–9.

## 76 Pollen food allergy syndrome (PFAS) in a Southeastern Ontario cohort of pollen allergic adults: determining prevalence and exploring risk factors

### Martha C. Ortega Santos^1^, Hannah Botting^2^, Sarah Garvey^2^, Lisa Steacy^2^, Eman Badawod^3^, Anne Ellis^2,3^

#### ^1^Queen’s University, Kingston, ON; ^2^Kingston Health Sciences Centre—KGH Site, Kingston, ON; ^3^University of Toronto, Toronto, ON

##### **Correspondence:** Martha C. Ortega Santos

*Allergy, Asthma & Clinical Immunology* 2025, **21(Suppl 1)**:76

**Background:** Pollen Food Allergy Syndrome (PFAS) is a complex condition in pollen-allergic individuals caused by cross-reactivity with pollen proteins and is typically characterized by adverse oral reactions to fresh fruits, vegetables, and nuts. In Canada, the prevalence of PFAS remains to be elucidated and risk factors are yet to be determined.

**Methods**: This cross-sectional study was conducted on a pollen-allergic population in Southeastern Ontario. Between June and July 2021, participants completed an online self-reported survey, using Qualtrics XM™, regarding food and seasonal allergic rhinoconjunctivitis (SAR) symptoms and severity. To confirm SAR status, responses were matched to on-file skin prick tests (SPT) for birch, timothy grass, and ragweed pollen. As each season’s most prevalent allergy-inducing pollens they are representative of each local allergy season. Statistical analyses were performed on SPSS.

**Results:** Of 194 participants with confirmed SAR, 158(81.4%) were allergic to timothy grass,135(69.6%) to ragweed, and 100(51.5%) to birch. Among these pollen-allergic participants, 73(37.6%) reported food allergies, of which 22(11.3%) were determined to have highly probable PFAS. The most common PFAS food triggers were kiwi 6(27.3%), pineapple 6(27.3%), peach5(22.8%), strawberry 5(22.8%), and banana 4(18.2%). The mean age of participants with PFAS was 42.1 years (SEM 2.6) and those without was 43.7 years (SEM 0.9) [p = 0.573]. Total nasal symptoms scores (TNSS) for each season were not significant between participants with PFAS and without [spring TNSS, p = 0.637; summer TNSS, p = 0.915; fall TNSS, p = 0.752]. Male sex [odds ratio (OR), 1.03, p = 0.950], urban areas [OR, 0.53; p = 0.199], and pollen sensitization [p = 0.793] were all found not to be significant risk factors for PFAS.

**Conclusions:** The prevalence of PFAS in this Canadian pollen-allergic cohort was found to be 11.3%. Of the PFAS risk factors explored—age, sex, area, pollen sensitization, and SAR symptom scores—none were statistically significant. Results suggest the need to explore biological outcomes to assess PFAS risk further.

## 77 The Thresholds In Food Allergy evaluaTion And predictioN (TITAN) multicentre cohort study: Allergy research network initial descriptive data

### Derek K. Chu^1^, Heather Le^1^, Rebecca Lee^2^, Anahita Dehmoobad Sharifabadi^1^, Alexandro Chu^1^, Raquel Ocvirk^1^, Anish Samanthapudi^1^, Silvia Zhang^1^, Raymond Luo^1^, Susan Waserman^1^, Michael Cyr^1^, Mary Messieh^1^, Rae Brager^1^, Noreen Choe^7^, Shannon French^6^, Jenny Garkaby^1^, Paul Oykhman^5^, Rebecca Pratt^6^, Arthur Chung^3^, Andrea Marrin^4^, Lehana Thabane^1^, Gordon Guyatt^1^

#### ^1^McMaster University, Hamilton, ON; ^2^University of Toronto, Toronto, ON; ^3^Oakville Allergy and Asthma Specialists, Oakville, ON; ^4^Brantford Allergy Clinic, Brantford, ON; ^5^Richmond Hill Allergy Clinic, Richmond Hill, ON; ^6^AVIVA Medical Diagnostics and Specialist Clinic INC, Hamilton, ON; ^7^Burlington Allergy Clinic, Burlington, ON

##### **Correspondence:** Anahita Dehmoobad Sharifabadi

*Allergy, Asthma & Clinical Immunology* 2025, **21(Suppl 1)**:77

**Background:** Food allergies affect approximately 7% of Canadians. Food-specific immunoglobulin E (IgE) levels and skin prick tests (SPTs) are the initial tests to diagnose food allergy. The gold standard is an oral food challenge (OFC), which is resource-intensive. There is a need for multi-centre tertiary centre and community data to characterize OFC outcomes and its predictors. The objective of this study is to provide initial descriptive data from our multi-centre cohort study.

**Methods**: All consecutive patients underwent OFCs at a tertiary care centre and four community health clinics across the Greater Toronto Hamilton Area between January 1, 2018, and January 1, 2023. We obtained demographic data, comorbid allergic diseases, medications, and OFC outcomes. Local research ethics board approved this study. Data was entered into REDCap and analyses were done using R.

**Results:** Of 4108 OFCs recorded, we present preliminary analyses of the first 1700 with analyzable data. The patients had a mean age of 12 ± 16 years and 827 (49%) identified as female. Patients underwent OFCs most commonly to tree nuts, peanut, egg, or milk. Of all OFCs, 318 patients (19%) experienced an allergic reaction during the OFC. Of these, 6 (0.3%) visited the emergency room and were discharged home. For positive OFCs, antihistamines (n = 140, 44%) were the most frequently administered medications.

**Conclusions:** The large number of OFCs and overall reaction of rate of 19% with mild or rapidly reversible allergic reactions support the need for, commonality, and safety of outpatient OFCs in hospitals and community allergy clinics. Our study provides a basis for the feasibility of large-scale data from a combination of tertiary care and multiple community sites. We call on more community clinics to join our registry to inform more standardized OFC guidelines and promote learning health systems.

## 78 Neffy (epinephrine nasal spray) development, from pharmacokinetics and pharmacodynamics to real-world data in pediatric food allergy patients

### Motohiro Ebisawa^1^, David M. Fleischer^2^, Henri Li^3^, Michael Klainer^3^, Richard Lockey^4^, Neetu Talreja^5^, Richard Lowenthal^6^, Sarina Tanimoto^6^

#### ^1^NHO Sagamihara National Hospital, Sagamihara, Japan; ^2^Children’s Hospital Colorado, Aurora, CO, USA; ^3^Institute for Asthma & Allergy, Chevy Chase, MD, USA; ^4^University of South Florida College of Medicine, Tampa, FL, USA; ^5^The Allergy Group and Treasure Valley Medical Research, Boise, ID, USA; ^6^ARS Pharmaceuticals, San Diego, CA, USA

##### **Correspondence:** Motohiro Ebisawa, Sarina Tanimoto

*Allergy, Asthma & Clinical Immunology* 2025, **21(Suppl 1)**:78

**Background:** No clinical trials were conducted to support pediatric doses of epinephrine injection products for the treatment of severe allergic reactions. Instead, FDA approval was based on epinephrine’s well-established safety and efficacy. During the development of *neffy*, ARS Pharmaceuticals, Inc. conducted a pharmacokinetic/pharmacodynamic study and an oral food challenge (OFC) study in pediatric patients.

**Methods**: The pharmacokinetic/pharmacodynamic study was a Phase 1, single-dose, open label study with allergy patients aged 4 to 17 (N = 42). The OFC study was a Phase 3 study in food allergy patients aged 6 to 17 who received *neffy* following onset of moderate anaphylaxis symptoms (N = 15). In both studies, patients received *neffy* 1 mg (15–30 kg) or 2 mg (≥ 30 kg).

**Results:** Mean epinephrine concentration–time profiles were similar between 1 and 2 mg (C_max_ of 690 and 651 pg/mL, respectively). Median time to resolve moderate anaphylaxis symptoms was 16 min, with no patient requiring a second dose of *neffy*. One patient developed a biphasic reaction 2 h and 45 min following *neffy* administration and received intramuscular epinephrine. Pharmacodynamic data from these studies were similar, except for a more pronounced decrease in diastolic blood pressure at early time points in younger patients in the OFC study. Adverse events were mild/moderate and resolved quickly.

**Conclusions:** *neffy* is the first epinephrine product studied in pediatric patients. Pharmacodynamic data were consistent between studies, and the OFC study demonstrated that *neffy* can resolve anaphylaxis symptoms. *neffy* is expected to be a safe and effective needle-free treatment option for pediatric allergy patients.

## 79 Pharmacokinetics and pharmacodynamics following repeat dosing of neffy (epinephrine nasal spray) versus intramuscular injection during induced allergic rhinitis

### John Oppenheimer^1^, Thomas Casale^5^, Jonathan Spergel^2^, David Bernstein^6^, Carlos A. Camargo, Jr^3^, Anne Ellis^7^, Richard Lowenthal^4^, Sarina Tanimoto^4^

#### ^1^UMDNJ Rutgers University School of Medicine, Newark, NJ, USA; ^2^Children’s Hospital of Philadelphia, Philadelphia, PA, USA; ^3^Massachusetts General Hospital, Harvard Medical School, Boston, MA, USA; ^4^ARS Pharmaceuticals, San Diego, CA, USA; ^5^Morsani College of Medicine, University of South Florida, Tampa, FL, USA; ^6^Bernstein Clinical Research Center and Division of Immunology, Allergy and Rheumatology, University of Cincinnati College of Medicine, Cincinnati, OH, USA; ^7^Kingston General Health Research Institute, Kingston, ON

##### **Correspondence:** Anne Ellis, Sarina Tanimoto

*Allergy, Asthma & Clinical Immunology* 2025, **21(Suppl 1)**:79

**Background:** While most patients respond to a single dose of epinephrine for anaphylaxis, approximately 10% require a second dose. ARS Pharmaceuticals investigated the pharmacokinetics and pharmacodynamics of repeat dosing of *neffy* in seasonal allergic rhinitis (SAR) patients after nasal allergen challenge (NAC), a potential impediment to epinephrine delivery.

**Methods**: This was a randomized, crossover study in 43 SAR patients with screening Total Nasal Symptom Scores and congestions scores of ≥ 5/12 and ≥ 2/3, respectively after a NAC. The pharmacokinetics and pharmacodynamics of repeat dosing of *neffy* 2.0 mg were compared to repeat doses of IM epinephrine 0.3 mg during rhinitis induced by NAC. *neffy* was administered to the same naris (right/right) or alternate naris (right/left).

**Results:** Compared to IM epinephrine, *neffy* (right/right) resulted in higher mean epinephrine concentrations through 240-min post-dose and *neffy* (right/left) resulted in higher mean epinephrine concentrations through 45 min post-dose, with greater mean peak concentration, C_max_, than that of IM (852/581 vs. 495; p < 0.05, p > 0.05). The mean changes in systolic blood pressure and pulse rate were greater following *neffy* (right/right)(right/left) through 60 min post-dose and Emax (21/18 mmHg vs. 13; p < 0.01, p < 0.05 and 22/22 vs. 14 bpm; p < 0.001, p < 0.01). All adverse events were mild.

**Conclusions:** Following twice dosing under SAR conditions, *neffy’s* pharmacokinetic and pharmacodynamic profiles were comparable to or better than IM regardless of naris delivery method. These findings are consistent with prior reports and suggest that *neffy* will be a safe and effective treatment option even in those with allergic rhinitis.

## 80 Successful administration of neffy (intranasal epinephrine) when provided with a two-dose carrying case – a human factor study

### Vivian Hernandez-trujillo^1^, Joel Brooks^2^, Raffi Tachdjian^3^, Brian Dorsey^4^, Richard Lowenthal^4^, Sarina Tanimoto^4^

#### ^1^Nicklaus Children’s Hospital, Miami, FL, USA; ^2^Columbia University Irving Medical Center, New York, NY, USA; ^3^David Geffen School of Medicine University of California, Los Angeles, CA, USA; ^4^ARS Pharmaceuticals, San Diego, CA, USA

##### **Correspondence:** Sarina Tanimoto

*Allergy, Asthma & Clinical Immunology* 2025, **21(Suppl 1)**:80

**Background:** neffy is an intranasal epinephrine sprayer for the treatment of severe allergic reactions. Because severe allergic reactions primarily occur outside of a hospital setting and epinephrine is typically administered by patients or caregivers, human factor (HF) studies are required to assure that the products can be administered safely and effectively. ARS Pharmaceuticals, Inc has previously conducted four HF-studies (n = 188) which have demonstrated that patients, caregivers, passer-byes, and children can administer *neffy* during a simulated allergy emergency and without prior training. A supplemental fifth study was conducted using the optional carrying case that was designed to hold two neffy sprayers.

**Methods**: This supplemental HF-study included 16 untrained subjects (eight adults and eight juveniles. Adults included both Type 1 allergy patients or caregivers, while the juveniles group included Type 1 allergy patients aged 10 to 17 years. Related tasks included loading the carry case; correctly opening the case during a simulated allergy emergency, and removing the sprayers and administering the product both once and twice (10 min apart) in the same nostril.

**Results:** All (100%) of adults and juvenile patients were able to successfully complete all tasks during a simulated severe allergic reaction without any use errors.

**Conclusions:** The *neffy* carrying case enables patients and caregivers to always have two *neffy* sprayers available and easily accessible. The results of this study demonstrated that untrained patients can properly follow written instructions. This study confirms that the user-friendly *neffy* carry case is also suitable for use by both adults and children.

## 81 Social media metrics during allergy awareness month: experiences from a food allergy research lab

### Halimat A. Ayoade^1^, Michael A. Golding^1,2^, Phillip Snarr^1,2^, Jennifer L. Protudjer^1,2,3^

#### ^1^Children’s Hospital Research Institute of Manitoba, Winnipeg, MB; ^2^Department of Pediatrics and Child Health, Rady Faculty of Health Sciences, Winnipeg, MB; ^3^Institute of Environmental Medicine, Karolinska Institutet, Stockholm, Sweden

##### **Correspondence:** Halimat A. Ayoade

*Allergy, Asthma & Clinical Immunology* 2025, **21(Suppl 1)**:81

**Background:** The month of May is Food Allergy Awareness Month, a time focused on increasing public awareness and understanding of food allergy-related topics. Social media provides opportunities to achieve these goals by giving health professionals and researchers with access to a large audience at low cost, and opportunities to examine levels of engagement with the posts, which in turn, can be quantified and studied. In order to better understand how to maximize impact, we examined differences in user engagement with various types of social media posts on the Protudjer Lab Instagram page during food allergy awareness month.

**Methods**: Throughout May 2024, we created food allergy awareness posts such as picture infographics, short educational videos, lab promotion (research, member profiles and on-going projects) and other promotional material (allergy-friendly recipes, event reminders and activities), published on the Instagram account @protudjerfoodallergylab. User engagement was quantified in terms of the number of users reached and the number interactions (i.e., likes, comments, saves, and shares) generated using Instagram’s built-in ‘insight’ function. Results were described using n/N and percentages. As this study involved no human or animal-based research, institutional ethical approval was neither required nor sought.

**Results:** Collectively, 25 posts (40% lab promotion posts, 36% infographics (24% pictures, 12% videos), 24% other promotional material (recipes, activities, and events)) were published. Of the three categories, lab promotion-related posts reached the highest number of users with an average of 124.6 accounts, followed by infographics and other promotional materials, at 73.11 and 64.5 accounts, respectively. In terms of engagement, lab promotion had the most engagement with accounts with an average of 10.5, followed by infographics and other promotional materials, at an average of 9 and 5.33 engagements, respectively.

**Conclusions:** The content and type of social media posts seemingly influence the engagement and reach of the post to users.

## 82 Provincial variations in triggers related to pediatric allergy emergency room visits from the cross-Canada anaphylaxis registry (C-CARE)

### Belinda V. Homer^1,2^, Michael A. Golding^1,2^, Sofianne Gabrielli^3^, Ann E. Clarke^4^, Marina Delli Colli^3^, Judy Morris^5^, Jocelyn Gravel^6^, Rodrick Lim^7^, Edmond S. Chan^8^, Ran D. Goldman^9^, Andrew O’Keefe^10^, Jennifer Gerdts^11^, Derek K. Chu^12^, Julia Upton^13^, Elana Hochstadter^14^, Jocelyn Moisan^15^, Adam Bretholz^16^, Christine McCusker^3^, Xun Zhang^17^, Elissa M. Abrams^1,2^, Elinor Simons^1,2^, Moshe Ben-Shoshan^3^, Jennifer L. Protudjer^1,2,18^

#### ^1^Children’s Hospital Research Institute of Manitoba, Winnipeg, MB; ^2^Department of Pediatrics and Child Health, Rady Faculty of Health Sciences, University of Manitoba, Winnipeg, MB; ^3^Division of Pediatric Allergy, Clinical Immunology and Dermatology, Montreal Children’s Hospital, McGill University Health Centre, Montreal, QC; ^4^Division of Rheumatology, Department of Medicine, Cumming School of Medicine, University of Calgary, Calgary, AB; ^5^Department of Emergency Medicine, Hôpital Sacré-Coeur, Montreal, QC; ^6^Department of Pediatric Emergency Medicine, Centre Hospitalier Universitaire Sainte-Justine, Université de Montréal, Montreal, QC; ^7^Division of Pediatric Emergency Medicine, Department of Pediatrics, Schulich School of Medicine & Dentistry, University of Western Ontario, London, ON; ^8^Division of Allergy, Department of Pediatrics, BC Children’s Hospital, University of British Columbia, Vancouver, BC; ^9^Division of Clinical Pharmacology and Emergency Medicine, Department of Pediatrics, BC Children’s Hospital, University of British Columbia, and the BC Children’s Research Institute, Vancouver, BC, ^10^Department of Pediatrics, Faculty of Medicine, Memorial University, St. John’s, NL; ^11^Executive Director, Food Allergy Canada, Toronto, ON; ^12^Division of Clinical Immunology & Allergy, Department of Medicine, and Department of Health Research Methods, Evidence & Impact, McMaster University, Hamilton, ON; ^13^Division of Immunology and Allergy, Department of Pediatrics, The Hospital for Sick Children, Department of Paediatrics, University of Toronto, Toronto, ON; ^14^Department of Pediatric Emergency Medicine, The Hospital for Sick Children, University of Toronto, Toronto, ON; ^15^Regional Medical Director of Emergency Medical Services of Outaouais, Outaouais, QC; ^16^Division of Pediatric Emergency Medicine, Department of Pediatrics, Montreal Children’s Hospital, Montreal, QC; ^17^Centre for Outcomes Research and Evaluation, Research Institute of McGill University Health Centre, Montreal, QC; ^18^Institute of Environmental Medicine, Karolinska Institutet, Stockholm, Sweden.

##### **Correspondence:** Belinda V. Homer

*Allergy, Asthma & Clinical Immunology* 2025, **21(Suppl 1)**:82

**Background:** The prevalence of pediatric allergies and their corresponding triggers are highly variable internationally. To further our understanding of regional differences, we aimed to explore triggers associated with pediatric allergy-related emergency room (ER) visits in Canada across provinces involved in the Cross-Canada Anaphylaxis Registry (C-CARE).

**Methods**: This was a retrospective cohort study of children participating in C-CARE. The sample included children aged ≤ 19 years with an anaphylactic reaction from five Canadian provinces. Records from Newfoundland and Labrador were excluded due to limited sample size. Triggers were categorized as peanut, tree nut, egg, nut, milk, shellfish, sesame, fish, kiwi, wheat, soy, other, unknown and multiple. Our study used descriptive statistics and logistic regression, reported as odds ratio (OR) and 95 percent confidence intervals (95% CI). This study was approved by the human research ethics boards in the participating centres.

**Results:** This analysis included data on 5903 ER visits from Quebec (QC, 68.0%), Ontario (ON, 9.7%), Manitoba (MB, 9.4%), British Columbia (BC, 9.0%), and Alberta (AB, 3.9%). There was a significant provincial variation in all triggers associated with pediatric allergy-related ER visits, except for soy. Among the most prevalent triggers, the proportion of ER visits related to peanut triggers was highest in MB (OR 1.27, 95% CI 1.01, 1.59), which differed significantly from AB and BC, but not from QC and ON. For tree nuts, QC (OR 1.54, 95% CI 1.27, 1.86) exhibited the highest proportions of related ER visits, significantly differing from BC and MB, but not from ON and AB. Unknown (OR 2.26, 95% CI 1.79, 2.86) and multiple (OR 1.90, 95% CI 1.33, 2.71) trigger categories also had the highest proportion of ER visits in MB, significantly differing from all other provinces.

**Conclusions:** Interprovincial variation in food triggers for anaphylaxis exists, highlighting the need for targeted interventions and improved public health awareness.

## 83 Are children with moderate-to-severe atopic dermatitis more likely to have multiple food allergies?

### Jennifer Takuski^3^, Stephanie Goguen^2^, Theo J. Moraes^4^, Stuart E. Turvey^5^, Piushkumar Mandhane^6^, Meghan B. Azad^2^, Padmaja Subbarao^4^, Elinor Simons^1,2,3^

#### ^1^Section of Allergy & Immunology, Department of Pediatrics & Child Health, University of Manitoba, Winnipeg, MB; ^2^Children’s Hospital Research Institute of Manitoba, Winnipeg, MB; ^3^Department of Pediatrics & Child Health, Winnipeg, MB; ^4^Hospital for Sick Children and University of Toronto, Toronto, ON; ^5^University of British Columbia, Vancouver, BC; ^6^University of Alberta, Edmonton, AB

##### **Correspondence:** Jennifer Takuski

*Allergy, Asthma & Clinical Immunology* 2025, **21(Suppl 1)**:83

**Background:** Atopic dermatitis (AD) affects an estimated 10–20% of children worldwide and often appears in the first year of life. Existing literature suggests a strong association between AD and the development of subsequent food and environmental allergies. We evaluated the relationships between moderate-to-severe AD (msAD) and sensitization to highly allergenic foods in the CHILD Cohort Study.

**Methods**: CHILD recruited expecting parents whose infants were born in 2009–2012. Clinical assessments were conducted at ages 1 and 3 years and included a history, physical examination, and skin prick testing to peanut, cow’s milk, and egg. MsAD was diagnosed by study physicians based on parent report in the past year and physical examination. We used multivariable logistic regression to compare associations between msAD and sensitization to any of peanut, cow’s milk, and egg allergens, adjusting for covariables including study centre, self-reported parental race, and number of older siblings.

**Results:** MsAD was present in 71 children (2.6%) at age 1 year and 103 (4.1%) at age 3 years. Sensitization was present to peanut (n = 138, 5.1%), cow’s milk (n = 50, 1.8%), and egg (n = 201, 7.4%) at 1 year and to peanut (n = 99, 5.4%), cow’s milk (n = 29, 1.1%), and egg (n = 58, 2.3%) at 3 years. After adjustment, msAD was associated with sensitization at 1 year to any of peanut, cow’s milk, or egg (adjusted OR 6.00, 95% CI 3.20–11.0), and on stratified analysis comparing any one of these allergens (adjusted OR 5.46, 95% CI 2.97–9.83), and at least 2 of these allergens (adjusted OR 6.19, 95% CI 2.13–16.1) versus none. The associations persisted for sensitization at age 3 years.

**Conclusions:** MsAD at ages 1 and 3 years was associated with sensitization to peanut, cow’s milk, or egg, and the association persisted among children with sensitization to any one or more than one of these food allergens.

## 84 IgE-sensitization and tolerance of baked products in food protein-induced enterocolitis syndrome (FPIES) to egg

### Lisa Liang^1,2^, Alennie Lopez^2^, Thomas Gerstner^1,2^, Elinor Simons^1,2^

#### ^1^Section of Allergy & Immunology, Department of Pediatrics & Child Health, University of Manitoba, Winnipeg, MB; ^2^Department of Pediatrics & Child Health, University of Manitoba, Winnipeg, MB

##### **Correspondence:** Lisa Liang

*Allergy, Asthma & Clinical Immunology* 2025, **21(Suppl 1)**:84

**Background:** FPIES is a non-IgE mediated food allergy. Baked egg tolerance occurs in some children with FPIES to egg, and may suggest a contribution from humoral immune responses, because in cell-mediated immune responses, peptides do not retain the original protein’s structural confirmation, and interaction would not be affected by the denaturation that occurs during baking. We evaluated IgE-mediated sensitization to egg and the ability to tolerate baked egg among children with FPIES to egg.

**Methods**: In this retrospective cohort study, we determined the rates of IgE-mediated sensitization to egg and tolerance of well-baked egg in a pilot cohort of children with FPIES to egg. We identified possible predictors of tolerance to well-baked egg.

**Results:** Among a preliminary cohort of 9 children, 3 with and 6 without IgE-mediated egg sensitization, the median age of presentation was 7 months (range 6–41 months), and 3 (33.3%) were male. The median time elapsed between ingestion of egg and initial reaction to egg was 120 min (range 60–120) in children with IgE-mediated egg sensitization, and 180 min (range 30–240) in those without IgE-mediated egg sensitization. Tolerance of baked egg was found in 100% of children with IgE-mediated egg sensitization, and 40% of those without IgE-mediated egg sensitization (p = 0.13). There was a personal history of other atopy in 33.3% of children with IgE-mediated egg sensitization, and 66.7% of those without sensitization (p = 0.29), and a family history atopy in a first degree relative in 33.3% of children with IgE-mediated egg sensitization, and 66.7% of those without IgE-mediated sensitization (p = 0.17).

**Conclusions:** In this small cohort of children with FPIES to egg, a higher proportion of children with egg sensitization was tolerant of baked egg. Further research is needed to understand the relationship between IgE-mediated egg sensitization and tolerance of baked egg in FPIES.

## 85 Latent time of symptoms and signs during food oral immunotherapy is associated with their severity grades

### Esther Zuo^1^, Danbing Ke^1^, Duncan Lejtenyi^1^, Liane Beaudette^1^, Xun Zhang^1^, Christine McCusker^1^, Julia E. Upton^2^, Edmond S. Chan^3^, Ann E. Clarke^4^, Philippe Bégin^5^, Eyal Grunebaum^2^, Bruce D. Mazer^1^, Moshe Ben-Shoshan^1^

#### ^1^Research Institute of the McGill University Health Centre, Division of Allergy, Immunology, and Dermatology, Department of Pediatrics, Montreal Children’s Hospital, Montreal, QC; ^2^Division of Immunology and Allergy, Hospital for Sick Children, Department of Pediatrics, University of Toronto, Toronto, ON; ^3^Division of Immunology and Allergy, BC Children’s Hospital, Department of Pediatrics, Faculty of Medicine, University of British Columbia, Vancouver, BC, ^4^Department of Medicine, Cumming School of Medicine, University of Calgary, Calgary, AB; ^5^Division of Allergy, Immunology and Rheumatology, Centre Hospitalier Universitaire (CHU) Sainte-Justine, Montreal, QC

##### **Correspondence:** Esther Zuo

*Allergy, Asthma & Clinical Immunology* 2025, **21(Suppl 1)**:85

**Background:** As a treatment option used to desensitize individuals to various food allergies, oral immunotherapy (OIT) is often associated with a wide spectrum of adverse reactions. These reactions range from minor oral tingling to life-threatening anaphylaxis involving multiple organ systems. The temporal features of such symptoms and signs have not been well characterized.

**Methods**: Pediatric participants with a confirmed diagnosis of food allergy to either egg, milk or peanut were enrolled and had regular in-clinic visits for up-dosing followed by at-home dosing. We recorded each symptom/sign that occurred in clinic. The severity of these symptoms/signs was scored according to the World Allergy Organization 2024 updated grading system. The latent time of each symptom/sign was determined based on the dosing time and the onset time of the documented symptoms/signs. Its association with reaction severity was assessed via linear mixed-effects model. The estimated marginal means of the latent times for all symptoms/signs were calculated via post-hoc analysis of the fitted mixed-effects model.

**Results:** Among 132 who underwent OIT dose escalation (egg = 18, milk = 82, peanut = 32), 122 reported adverse reactions during OIT. Of these reactions, 25 symptoms and signs were recorded, mainly involving mucocutaneous (43.2%), respiratory (35.7%) and gastrointestinal (20.2%) systems. Estimated marginal means of the latent times for these symptoms/signs ranged from 2.2 to 40.7 min. Longer latent times of the allergic symptoms and signs were associated with higher severity grades of these symptoms/signs (CE 9.9, CI 8.1–11.7, p < 0.001), epinephrine use (CE 14.8, CI 9.9–19.7, p < 0.001), and respiratory symptoms/signs (CE 5.9, CI 1.8–9.9, p < 0.005). Shorter latent time was associated with milk (CE -9.7, CI -16.3—-3.1, p < 0.004) and peanut (CE -4.7, CI -12.4—-3.1, p = 0.24) as compared to egg.

**Conclusions:** Longer latent time is associated with symptoms and signs of higher severity grades observed during OIT.

## 86 Does component IgE testing add value in peanut allergy with negative total IgE? implications for diagnostics and cost

### Harinder Pal Gill, Yue (Jennifer) Du, Aislinn Vey, Ernie Avilla, Susan Waserman

#### McMaster University, Hamilton, ON

##### **Correspondence:** Harinder Pal Gill, Yue (Jennifer) Du

*Allergy, Asthma & Clinical Immunology* 2025, **21(Suppl 1)**:86

**Background:** Peanut allergy is a growing, potentially life-threatening condition. Although double-blind placebo-controlled food challenge (DBPCFC) is the diagnostic gold standard, it is risky and resource-intensive. Peanut IgE component testing aims to reduce DBPCFC use but needs further validation. This study investigates patients with negative whole peanut IgE but positive component IgE to determine characteristics justifying component tests.

**Methods**: We retrospectively reviewed charts of patients assessed for peanut allergy at Hamilton allergy clinics, (HHS-McMaster, Firestone Institute of Respiratory Health (FIRH), community clinics) from 2015 to 2022. Data included demographics, clinical histories, specific IgE levels to whole peanut and components, skin prick tests, and diagnoses. Ethics was approved by McMaster REB. We identified patients with whole peanut IgE negative but component IgE positive (Ara h1, 2, 3, 8, 9) using established 0.35 kU/L cut-off.

**Results:** Out of 413 patients, 4 were identified as IgE negative component positive (INCP). The total cohort included 109 females and 304 males, with 2 females and 2 males in the INCP group. Median age: 8 years (total) and 6.5 years (INCP). Serum IgE: 14.7 kU/L (total) and 0.18 kU/L (INCP). Three of the four INCP patients had positive SPT, averaging 3.75 mm. Two had positive Ara h2 levels (0.6 and 0.39), one had a positive Ara h8 level (4.06), and one had positive Ara h2 (64.8) and Ara h3 (5.9) levels. One patient developed hives upon peanut exposure, while two reported no symptoms, and one was never exposed.

**Conclusions:** Among over 400 patients, only 4 patients exhibited discordant results. These patients showed mild or no reactions to peanuts, suggesting limited additional diagnostic benefit from component testing when whole peanut IgE is negative. Routine component panel testing may not be necessary when whole peanut IgE is negative, potentially reducing costs without compromising care. Further research is needed to validate these findings.

## 87 Safety assessment of NSAIDs during epicutaneous immunotherapy for peanut allergy

### Julia E. Upton^1^, Philippe Begin^2^, David M. Fleischer^3^, Henry T. Bahnson^4^, Todd Green^4,5^, A. W. Burks^5^, Hugh Sampson^4,6^

#### ^1^The Hospital for Sick Children, Division of Immunology and Allergy, Department of Paediatrics, University of Toronto, Toronto, ON; ^2^Centre Hospitalier Universitaire Sainte-Justine, Montreal, QC; ^3^Children’s Hospital Colorado, University of Colorado School of Medicine, Aurora, CO, USA; ^4^DBV Technologies, Châtillon, France; ^5^University of North Carolina at Chapel Hill School of Medicine, Chapel Hill, NC, USA; ^6^Icahn School of Medicine at Mount Sinai, New York, NY, USA

##### **Correspondence:** Julia E. Upton

*Allergy, Asthma & Clinical Immunology* 2025, **21(Suppl 1)**:87

**Background:** Certain cofactors may lead to more severe allergic reactions and/or decrease the reactivity threshold in food-allergic children.^1^ Nonsteroidal anti-inflammatory drugs (NSAIDs) are a commonly identified cofactor in anaphylaxis to foods.^2,3^ This analysis evaluates whether NSAID use is associated with differences in adverse event (AE) outcomes in participants receiving epicutaneous immunotherapy with the VIASKIN® peanut patch (VP250) vs placebo.

**Methods**: Peanut-allergic participants aged 1 through 3 years (EPITOPE) or 4 through 11 years (PEPITES) were randomized to VP250 vs placebo for 12 months in these phase 3 studies. A pooled analysis evaluated a potential association between NSAID use and three AEs of interest: treatment-related anaphylaxis, treatment-related epinephrine use, and treatment-related AEs leading to systemic corticosteroid use.

**Results:** Of 718 participants (VP250: 482, placebo: 236), 320 (44.6%) reported any NSAID use and 398 (55.4%) did not. There were similar rates of NSAID use between treatment arms, 219/482 (45.4%) for VP250 and 101/236 (42.8%) for placebo. There were also similar numbers of treatment-related AEs among participants with any NSAID use vs no NSAID use (median: 5 vs 4 events for VP250 and 3 vs 1 event for placebo). Examination of the timing of NSAID use and AEs did not suggest an association. Among VP250-treated participants who reported any NSAID use and experienced treatment-related anaphylaxis (3.2%), treatment-related epinephrine use (2.3%), or treatment-related systemic corticosteroid use (3.2%), NSAID use was concurrent in only one (started 5 months prior to AE).

**Conclusions:** Although NSAID use is a known cofactor for anaphylactic reactions, these results suggest NSAID use was not associated with treatment-related anaphylaxis, epinephrine use, or systemic corticosteroid use among VP250 participants in 2 large phase 3 studies. NSAID use has not been restricted in VP250 clinical trials, and these data suggest that such restrictions would not be necessary if VP250 is approved for the treatment of peanut allergy.

**References:**

1. Muñoz-Cano R, San Bartolome C, Casas-Saucedo R, et al. *Front Immunol*. 2021;11:623,071.
2. Romano A, Gaeta F, Caruso C, Fiocchi A, Valluzzi RL. *J Allergy Clin Immunol Pract.* 2023;11(6):1843–1853.e1.
3. Shin M. *Clin Exp Pediatr*. 2021;64(8):393–399.

## 88 An evaluation of food allergy management practices in Canadian and American schools

### April Quill^1,2^, Michael A. Golding^1,2^, Lisa M. Bartnikas^3,4^, Jennifer L. Protudjer^1,2,5^

#### ^1^University of Manitoba, Winnipeg, MB; ^2^Children’s Hospital Research Institute of Manitoba, Winnipeg, MB; ^3^Boston Children’s Hospital, Boston, MA, USA; ^4^Harvard University, Boston, MA, USA; ^5^Karolinska Institutet, Stockholm, Sweden

##### **Correspondence:** April Quill

*Allergy, Asthma & Clinical Immunology* 2025, **21(Suppl 1)**:88

**Background:** Most children, including the estimated 7% of children with food allergy, spend the majority of their waking hours in school. Yet, variations in school-based food allergy practices are believed to exist. We aimed to explore the differences between epinephrine management in schools in Canada and the United States (US), with a secondary focus on differences across school locations and types.

**Methods**: Parents/caregivers with children who have Immunoglobulin E (IgE)-mediated food allergy were recruited through social media to complete an online survey evaluating the school’s stock epinephrine, epinephrine storage locations, children’s self-carriage, school type and location (i.e., country and rurality), and the child’s age in years. Descriptive statistics and logistic regression were used to report results as odds ratios (OR) and 95% confidence intervals (CI). This study was approved by the University of Manitoba Health Research Ethics Board.

**Results:** 169 participants (23 Canada, 146 US) were included. The average child age was 4.91 ± 3.12 years. Common food allergies were tree nuts (43.2%), peanuts (30.2%), and shellfish (21.9%). No significant differences were found between Canada and the US regarding stock epinephrine or storage locations. Compared to public schools, charter schools had lower odds of having stock epinephrine (OR 0.21; CI 0.05–0.94). Compared to rural schools, urban schools had greater odds of storing epinephrine in classrooms (OR 22.61; CI 2.35–217.32) and main offices (OR 23.24; CI 2.90–186.21), and lower odds in health offices (OR 0.20; CI 0.56–0.69). Older children (OR 1.22; CI 1.05–1.42) and those attending urban schools (OR 11.40; CI 1.26–103.49) had greater odds of epinephrine self-carriage compared to younger children and those in rural schools.

**Conclusions:** Differences were identified between urban and rural schools. Best practices and ongoing knowledge translation efforts are essential to ensure that epinephrine is consistently available in known, easily-accessible locations to immediately treat anaphylaxis in schools.

## 89 Symptomatology and management of adult anaphylaxis according to trigger: a cross-sectional study

### Roy Khalaf^1^, Connor Prosty^1^, Christine McCusker^1^, Ann E. Clarke^2^, Adam Bretholz^1^, Mohammed Kaouache^1^, Judy Morris^3^, Rodrick Lim^3^, Edmond S. Chan^4^, Ran D. Goldman^4^, Andrew O. Keefe^5^, Jennifer Gerdts^6^, Derek Chu^7^, Julia Upton^8^, Elana Hochstadter^8^, Xun Zhang^1^, Jennifer L. Protudjer^9^, Elissa M. Abrams^9^, Elinor Simons^9^, Juan Ruiz^4^, Moshe Ben-Shoshan^1^

#### ^1^McGill University, Montreal, QC; ^2^University of Calgary, Edmonton, AB; ^3^Université de Montréal, Montreal, QC; ^4^University of British Columbia, Vancouver, BC; ^5^Memorial University, St John’s, NL; ^6^Food Allergy Canada, Toronto, ON; ^7^McMaster University, Hamilton, ON; ^8^University of Toronto, Toronto, ON; ^9^University of Manitoba, Winnipeg, MB

##### **Correspondence:** Roy Khalaf

*Allergy, Asthma & Clinical Immunology* 2025, **21(Suppl 1)**:89

**Background:** Anaphylaxis is an acute life-threatening allergy, most commonly provoked by food, venom, or drugs. There is limited data regarding differences in symptomatology between anaphylaxis provoked by different triggers.**:** Anaphylaxis is an acute life-threatening allergy, most commonly provoked by food, venom, or drugs. There is limited data regarding differences in symptomatology between anaphylaxis provoked by different triggers.

**Methods**: We conducted a cross-sectional study recruiting adult patients with anaphylactic reactions across 8 emergency departments (ED) and 1 electronic medical service (EMS) in Canada. Univariate and multivariate regression models were used to evaluate symptoms involving all patients with the outcome of drug-induced anaphylaxis (DIA), venom-induced anaphylaxis (VIA), peanut-induced anaphylaxis (PIA), shellfish-induced anaphylaxis (SIA), tree-nut induced anaphylaxis (TIA) and nut-induced anaphylaxis (NIA). We assessed comorbidities associated with severe reactions, stratified by triggers listed above. Additionally, we evaluated the association of each trigger with treatment through regression models involving all patients with medications used as outcome and anaphylaxis triggers used as independent variables.

**Results:** Hypotension was more likely associated with VIA (aOR = 1.08,95%CI = 1.03–1.13,P = 0.042) and DIA (aOR = 1.20,95%CI = 1.11–1.30, P < 0.01). Pruritus was less likely associated with TIA (aOR = 0.97, 95%CI = 0.95–0.99, P = 0.026). Throat tightness was more likely associated with TIA (aOR = 1.04, 95% CI = 1.01–1.06, P < 0.01). When adjusted for age at reaction and male sex, alcohol was more likely associated (aOR = 1.51,95% CI = 1.04–2.19, P = 0.035) with NIA. TIA was more likely associated with inpatient epinephrine (aOR = 2.05, 95%CI = 1.16–3.64, P = 0.014). DIA was less likely associated with outpatient antihistamine (aOR = 0.68, 95%CI = 0.48–0.89, P < 0.01) whereas TIA was more likely associated with outpatient antihistamine (aOR = 1.81,95%CI = 1.03–3.19,P = 0.040).

**Conclusions:** Our study underscores associations between specific triggers, clinical manifestations and managements, such as the potential link between TIA and throat tightness and hypotension and VIA. Identifying such associations can aid with the prompt diagnosis of anaphylaxis in patients presenting to the ED, leading to swifter treatment initiation and improving overall outcomes.

## 90 Oral food challenge safety in the post-oral immunotherapy era

### Min Jung Kim^1^, Adam P. Sage^1^, Tiffany Wong^2,3^, Raymond Mak^2,3^, Stephanie C. Erdle^2,3^, Lianne Soller^2,3^, Edmond S. Chan^2,3^

#### ^1^Faculty of Medicine, University of British Columbia, Vancouver, BC; ^2^Division of Allergy, Department of Pediatrics, Faculty of Medicine, University of British Columbia, Vancouver, BC; ^3^British Columbia Children’s Hospital, Vancouver, BC

##### **Correspondence:** Min Jung Kim

*Allergy, Asthma & Clinical Immunology* 2025, **21(Suppl 1)**:90

**Background:** We previously published data on the safety of oral food challenges (OFCs) at BC Children’s Hospital (BCCH), which demonstrated high rates of anaphylaxis requiring epinephrine (15.0%) [1]. Since 2019, oral immunotherapy (OIT) to treat food allergies has been widely adopted at our center. As a result, an increasing proportion of OFCs are being completed to assess desensitization after OIT, which carries a significantly lower risk of anaphylaxis than OFCs completed for diagnostic purposes [2]. Thus, we sought to compare the safety of OFCs between the pre-OIT (2014–2017) and post-OIT (2019–2021) eras.

**Methods**: We conducted a retrospective chart review as part of a quality improvement project, of OFCs completed between 2019–2021 at BCCH. Data on OFCs from 2014–2017 were previously collected and published [1]. Here, we compare rates of anaphylaxis requiring epinephrine and rates of failed OFCs between the pre-OIT (2014–2017) and post-OIT (2019–2021) cohorts.

**Results:** A total of 353 pre-OIT OFCs, and 590 post-OIT OFCs were completed, with 92 (15.6%) OFCs in the post-OIT era assessing desensitization following OIT. One hundred fifteen (32.6%) pre-OIT versus 156 (26.4%) post-OIT OFCs failed (p = 0.04). In comparison to the pre-OIT cohort, we found lower rates of anaphylaxis requiring epinephrine in the post-OIT cohort (15.0% vs. 8.3%; p = 0.001). Stratifying the data, post-March 2020 there were more children on OIT compared to pre-March 2020 (27.3% vs. 7.2%) and less anaphylaxis (5.3% vs. 10.4%; p = 0.03).

**Conclusions:** Compared with the pre-OIT era, our data demonstrate the increased safety of OFCs performed in the post-OIT era, with fewer failed OFCs, and fewer anaphylactic reactions requiring epinephrine. With the introduction of OIT, there have been changes in clinical practice whereby patients with a strong suspicion of food allergy can be offered OIT rather than performing a diagnostic OFC with a higher risk of anaphylaxis.

**References:**

1. Soller L, Teoh T, Baerg I, Wong T, Hildebrand KJ, Cook VE, Biggs CM, Lee N, Yaworski L, Cameron SB, Chan ES. **Extended analysis of parent and child confidence in recognizing anaphylaxis and using the epinephrine autoinjector during oral food challenges**. *J Allergy Clin Immunol Pract*. 2019; **7**:693–5.
2. Soller L, Abrams EM, Carr S, Kapur S, Rex GA, Leo S, McHenry M, Vander Leek TK, Yeung J, Cook VE, Wong T. **First real-world effectiveness analysis of preschool peanut oral immunotherapy**. *J Allergy Clin Immunol Pract*. 2021; **9**:1349–56.

## 91 Patient perspective on self-care models to increase access to early interventions in infants with food allergy

### Anna Voia^1^, Marie-Josée Bettez^2^, Catherine Lord^3^, Philippe Bégin^1^

#### ^1^Section of Allergy, Department of Pediatrics, CHU Sainte-Justine, Montreal, QC; ^2^Déjouer les allergies, Québec, QC; ^3^Immerscience, Québec, QC

##### **Correspondence:** Anna Voia

*Allergy, Asthma & Clinical Immunology* 2025, **21(Suppl 1)**:91

**Background:** Food allergies affect over 4% of children and 2% of adults in Canada, with the burden mainly arising from the psychosocial impact on patients and families. Oral immunotherapy (OIT), which is recommended as a treatment option by Canadian guidelines since 2020, can induce complete remission if started within the first year of life. However, access to specialized care remains limited, leading many children to miss the window of opportunity to induce clinical tolerance. While OIT is traditionally offered by allergist, there is no literature to our knowledge addressing patients’ views on potential alternative care models. The objective of this study was to explore views of parents regarding barriers and facilitators, risk tolerance, required support and overall acceptability of self-care approaches to early infant OIT.

**Methods**: Parents of food allergic children were recruited from the “Déjouer les allergies” community, using a reasoned sampling method based on demographic traits and opinions on OIT. Semi-structured interviews were conducted and analyzed thematically using a qualitative moderately inductive approach.

**Results:** Preliminary analyses suggest that participants were open to accepting a certain level of risk to increase access to OIT, even without direct supervision from an allergist. Barriers for parents included the distance to care centers, apprehension of allergic reactions, and lack of support. Participants were open to OIT monitoring by primary care professionals such as family doctors and clinical nurses but emphasized the importance of proper medical training for treatment initiation and supervision. Support modalities such as a hotline and standardized dosage logs increased the perceived acceptability of self-care options.

**Conclusions:** Parents are open to alternative care models to increase access to OIT. Including patient perspective in the development of these new models will be crucial to ensure acceptability and success.

## 92 Impact of reducing post-vaccination observation period on the risk of delayed identification of serious adverse events following immunization

### Anabel Gil^1^, Cathy Yan^1,3^, Elissa M. Abrams^1,2^, Joseline Zafack^1^, Nicole Forbes^1^, Kelsey Young^1^, Pamela Doyon-Plourde^1^

#### ^1^Public Health Agency of Canada, Ottawa, ON; ^2^University of Manitoba, Public Health Agency of Canada, Ottawa, MB; ^3^University of British Columbia, Vancouver, BC

##### **Correspondence:** Elissa M. Abrams

*Allergy, Asthma & Clinical Immunology* 2025, **21(Suppl 1)**:92

**Background:** In Canada, post-vaccination monitoring is typically 15 min, unless there are specific concerns. During the COVID-19 pandemic, shortening this period was considered to prevent SARS-CoV-2 transmission. This rapid review assesses the onset time of serious adverse events following immunization (AEFI) such as an anaphylaxis and the impact of shorter post-vaccination observation periods on detecting events requiring immediate medical intervention.

**Methods**: Embase and MEDLINE were searched up to March 7, 2023, to identify studies on the timing of serious AEFIs (i.e., anaphylaxis, syncope, and seizure). Studies reporting events that occurred within 30 min of vaccination with specific time to symptoms onset of at least a subset of AEFIs were included. Screening and data extraction were completed by one reviewer and independently validated by another.

**Results:** Out of 3,375 records, 60 articles were included. Most studies reported data on anaphylaxis (n = 43), then syncope (n = 17), and seizures (n = 9). Currently, 37% (n = 22) underwent data extraction and were included in preliminary analyses. Among the anaphylaxis cases, 868 (88%) reported time to onset where the majority (n = 497, 57.3%) occurred within 30 min of vaccination. Of those with onset reported within the first 15 min (n = 57), 70.2% occurred within 5 min, and 93.0% within 10 min of vaccination. Among syncope cases, 698 (50.7%) had time to onset available with most 89.3% (n = 623) occurring within 30 min of vaccination. Of those occurring within the first 15 min (n = 470), 72.1% occurred within 5 min, and 91.9% occurred within 10 min of vaccination. For seizure events captured, only 17 (24.3%) cases had information on time to onset with most (88.2%) happening more than 30 min after vaccination.

**Conclusions:** This rapid review is important to inform guidelines for post-vaccination observation period during public health emergencies and for pandemic preparedness. Data extraction, quality assessment and results synthesis will be completed in the coming months.

## Immunology

## 93Burden of disease of respiratory syncytial virus in older adults and adults considered at high risk of severe infection

### Elissa M. Abrams^1,2^, Pamela Doyon-Plourde^1^, Phaedra Davis^1,4^, Liza Lee^1^, Abbas Rahal^1^, Nicholas Brousseau^3^, Winnie Siu^1,4^, April Killikelly^1^

#### ^1^Public Health Agency of Canada, Ottawa, ON; ^2^University of Manitoba, Winnipeg, MB; ^3^Institut national de la santé publique du Québec, Quebec, QC; ^4^University of Ottawa, Ottawa, ON

##### **Correspondence:** Elissa M. Abrams

*Allergy, Asthma & Clinical Immunology* 2025, **21(Suppl 1)**:93

**Background:** The respiratory syncytial virus (RSV) vaccine landscape has changed significantly with several new vaccines being authorized for adults in Canada. While RSV causes a significant burden in children, the burden is not well characterized in adults. This study compiles evidence from a rapid literature review and the Canadian Institute for Health Information (CIHI) Discharge Abstract Database (DAD) to present a comprehensive picture of the RSV burden in adults.

**Methods**: Four databases were searched for studies published between January 1, 1995 and September 1, 2023 reporting data on adult RSV outpatient visits, hospitalizations, intensive care unit (ICU) admissions, and deaths. Data on patients admitted to an acute care facility with RSV between September 2010 to August 2020 and September 2021 to August 2023 were obtained from the CIHI DAD. RSV-associated hospitalizations were defined using clinical diagnostic codes. Aggregated rates were calculated using population data from provinces and territories, except Quebec.

**Results:** 26 studies were included in the rapid review. RSV outpatient visits were responsible for 4.7–7.8% of symptomatic respiratory tract infections in adults 60 and older. Hospitalization incidence varied between studies, but the risk increased consistently with age. Among hospitalized adults, approximately 10% required ICU admission and the case fatality ratio (CFR) ranged from 5–10%. The incidence of all clinical outcomes increased with age and comorbidities. Results from the CIHI DAD showed rates of hospitalization, ICU admission and death increased with age. In adults 50 and older, the average hospitalization rate was 16 per 100,000. Overall, 16% of hospitalizations resulted in ICU admission and in-hospital CFR was 9%.

**Conclusions:** Canadian hospitalization data support the rapid review findings where the incidence of severe clinical outcomes increased with age and the presence of comorbidities. The combination of these analyses provides a novel perspective on the burden of RSV in older adults to support vaccine program decision-making.

**References:**

Abrams EM et al.Burden of Disease of Respiratory Syncytial Virus in Older Adults and Adults Considered at High Risk of Severe Infection. MedRxiv doi: 10.1101/2024.03.18.24304476

## 94 Burden of diseased of respiratory syncytial virus in infants and young children

### Elissa M. Abrams^1^, Pamela Doyon-Plourde^1^, Phaedra Davis^1^, Nicholas Brousseau^2^, Andrea Irwin^3^, Winnie Siu^4^, April Killikelly^1^

#### ^1^Public Health Agency of Canada, Ottawa, ON; ^2^Institut national de la santé publique du Québec, Quebec, QC; ^3^Yukon Communicable Disease Control, Health and Social Services, Government of Yukon, Whitehorse, YT; ^4^University of Ottawa, Ottawa, ON

##### **Correspondence:** Elissa M. Abrams

*Allergy, Asthma & Clinical Immunology* 2025, **21(Suppl 1)**:94

**Background:** The Respiratory Syncytial Virus (RSV) vaccine landscape has shifted rapidly for infants and young children with two new immunization products approved in Canada in 2023. While there is significant evidence regarding RSV burden of disease in high-risk infants, less evidence is available in healthy infants and young children. This rapid review aims to summarize the available evidence on RSV burden of disease in infants and young children.

**Methods**: Four databases were searched for studies published between January 1, 1995 to April 10, 2023. Canadian respiratory virus surveillance experts were contacted for additional data. One reviewer screened the articles for eligibility. A second reviewer validated the exclusion lists. Systematic reviews (SRs) and primary studies reporting data on outpatient visits, hospitalizations, intensive care unit (ICU) admissions, and deaths associated with RSV in infants and children from high-income countries were included.

**Results:** Overall, 17 studies were included, in addition to surveillance data from one Canadian territory (Yukon). Medically attended RSV infections are frequent during infancy and early childhood, with approximately 10–20% of infants seeking care for RSV in a season (n = 4 studies). Rates of RSV hospitalization decreased with increasing age (n = 11 studies) with the highest rates observed in infants under six months of age (> 1% annually). The majority (> 70%) of hospitalized children have no underlying medical conditions. Overall, approximately 5% of RSV hospitalizations needed ICU admission (n = 7 studies), with higher rates among those with risk factors. Mortality was very low (n = 4 studies).

**Conclusions:** While the risk of severe outcomes is greater in high-risk infants and children, the healthcare burden is greatest in healthy infants born full-term. Robust surveillance systems will be crucial for evaluating the public health impact of new RSV immunization programs. This review contributes to the literature, aiding in characterizing the RSV burden in Canada and guiding RSV immunization strategies for infant protection.

**References:**

Abrams EM et al. Burden of disease of RSV in infants, children and pregnant women and people. Can Commun Dis Rep 2024;50(1/2):1–15. 10.14745/ccdr.v50i12a01

## 95 Feasibility and resource utilization of nurse-administered subcutaneous immunoglobulin therapy in antibody deficiency: a cross-sectional study

### Darshee Ghia, Pranavi Thota, Taylor Ritchie, Heli Rana, Ranvir Minhas, Ranvir Minhas, Adil Adatia, Bruce Ritchie

#### University of Alberta, Edmonton, AB

##### **Correspondence:** Darshee Ghia

Allergy, Asthma & Clinical Immunology 2025, **21(Suppl 1)**:95

**Background:** Primary and secondary antibody deficiencies (PAD and SAD) are common immunodeficiency syndromes often necessitating lifelong immune globulin replacement therapy (IRT). Both intravenous immunoglobulin (IVIg) and subcutaneous immunoglobulin (SCIg) have demonstrated efficacy in these conditions. SCIg is usually self-administered at home whereas IVIg is nurse-administered at infusion clinics. The feasibility of implementing nurse-administered SCIg programs within hospital infusion clinics remains unstudied.

**Methods**: We conducted a cross-sectional study assessing nurse-administered SCIg. Nursing staff administered SCIg using quadrifurcated needle sets. Data collected included infusion duration, chair time, nursing time, health status measured using SF36v2, and treatment satisfaction measured by the Life Quality Index (LQI). Time measures were expressed as minutes/month to account for more frequent SCIg dosing compared to IVIg. All subjects provided written informed consent.

**Results:** There were 13 SCIg patients and 13 IVIg patients included. Median monthly times (SCIg vs. IVIg) were as follows: infusion: 107.9 (95% Cl: [40, 184]) vs.122 (95% Cl: [99, 167]) minutes, chair: 180.66 (95% Cl: [96.2, 204.1]) vs.156 (95% Cl: [117, 207]) minutes, and nurse: 54.7 (95% Cl: [46.8, 98]) vs. 36.75 (95% Cl: [28, 58.5]) minutes. Patients reported high satisfaction with both treatments. Average SCIg LQI scores (SD) were 6.15/7 (0.8) for treatment interference, 4.61/7 (2.1) for therapy-related problems, and 6.69/7 (0.85) for therapy setting. IVIg LQI scores (SD) were 6.36/7 (1.28), 5.79/7 (2.01), and 6.36/7 (1.65), respectively.

**Conclusions:** Nurse-administered SCIg appears feasible and comparable to IVIg in terms of resource utilization. Nurse-administered SCIg may be a useful modality for well-selected patients who are not suitable for home treatment.

## 96 Clinical and genetic findings of > 7,300 individuals tested via the navigateAPDS sponsored genetic testing program

### Jenny Garkaby^1^, Julia Upton^2^, Nami Park^3^, Brian Hartline^3^, Mike Samad^3^, Anurag Relan^3^, Joseph Harper^3^, Heather McLaughlin^3^

#### ^1^McMaster Children’s Hospital, Department of Pediatrics, Division of Rheumatology, Immunology and Allergy, McMaster University, Hamilton, ON; ^2^The Hospital For Sick Children, Clinical Immunology and Allergy, Dept. of Pediatrics, University of Toronto, Toronto, ON; ^3^Pharming Healthcare, Warren, NJ, USA

##### **Correspondence:** Jenny Garkaby

*Allergy, Asthma & Clinical Immunology* 2025, **21(Suppl 1)**:96

**Background:** Activated phosphoinositide 3-kinase (PI3K) δ syndrome (APDS) results from disease-associated variants in the *PIK3CD* and *PIK3R1* genes that lead to hyperactivity of the PI3K delta pathway and progressive immune deficiency and/or dysregulation. navigateAPDS, a no-charge, sponsored genetic testing program, was established in the US and Canada to help reduce barriers to testing. This study aims to review clinical and genetic findings from the navigateAPDS cohort, including 44 Canadian participants.

**Methods**: Consented individuals who fulfilled ≥ 2 APDS-related clinical, laboratory, and/or family history criteria were eligible for comprehensive immunodeficiency genetic testing at Invitae Corporation.

**Results:** 7356 patients from 1346 providers were tested between March 2021 and June 2024. The median age at testing was 35 years, 48.8% were < 18 years, and 62.1% were female. A total of 590 patients (8.0%) received a positive molecular diagnosis (MolDx). *PIK3CD* or *PIK3R1* variants were identified in 156 individuals (2.1%) including 35 individuals from 27 families who received an APDS MolDx (e.g., likely pathogenic or pathogenic *PIK3CD* or *PIK3R1* variant) and 122 individuals who received a variant of uncertain significance (VUS). Of the individuals with a positive APDS MolDx, 10/35 (28.5%) harbored a disease-causing variant not previously published in association with APDS.

**Conclusions:** The navigateAPDS program has identified multiple individuals with a positive APDS MolDx, including several with novel variants, demonstrating the ability of the program to assist clinicians in accurately diagnosing their patients. Additional clinical, functional, and family segregation studies will aid in VUS interpretation and offer additional insights into the genetic underpinnings of APDS and its clinical manifestations.

## 97 Incidence of Rituximab induced hypogammaglobinemia in pediatric nephrotic syndrome patients: a systematic review and meta-analysis

### Jason Chung^1^, Andrew Yu^2^, Zinnia Chung^3^, Cal Robinson^4^, Arvind Bagga^5^, Joycelyne Ewusie^3^, Rajiv Sinha^6^, Yuanxin Xue^7^, Rahul Chanchlani^3^

#### ^1^University of Western Ontario, London, ON; ^2^University of Alberta, Edmonton, AB; ^3^McMaster University, Hamilton, ON; ^4^The Hospital for Sick Children, Toronto, ON; ^5^All India Institute of Medical Sciences, New Delhi, DL, India; ^6^Institute of Child Health, Kolkata, BR, India; ^7^University of Toronto, Toronto, ON

##### **Correspondence:** Jason Chung

*Allergy, Asthma & Clinical Immunology* 2025, **21(Suppl 1)**:97

**Background:** Rituximab is a chimeric anti-CD20 monoclonal antibody used to treat complicated pediatric nephrotic syndrome, as an effective steroid-sparing agent [1]. Hypogammaglobinemia is a rare, but important adverse event of rituximab associated with severe infections and mortality. The risk of hypogammaglobinemia in the pediatric population has been previously reported to be higher than adults [2]. The objective of this review is to determine the incidence of hypogammaglobinemia and concomitant infections in pediatric nephrotic syndrome patients receiving rituximab.

**Methods**: A systematic literature search was conducted on five databases from 1974–2024 for English publications. Randomized and non-randomized studies with pediatric (mean age < 18 years) nephrotic syndrome patients receiving at least one dose of rituximab were included. Hypogammaglobinemia was defined by IgG levels less than two standard deviations below the age-matched reference range.

**Results:** Thirty-six studies (n = 3,495) were eligible for meta-analysis. The pooled incidence of hypogammaglobinemia was 11.4% (95% CI: 6.4, 19.5). Random effects meta-regression demonstrated no significant effect of the length of follow up, age, sex or cumulative dose. Of 517 isolated episodes of hypogammaglobinemia, 93(18)% infections were reported, of which 4 patients died, 2 patients required intensive care, 2 patients had chronic infections and 73 patients had severe infection such as varicella-zoster infections, sepsis, gastrointestinal and respiratory infections. The most common co-administered medications included mycophenolate mofetil, corticosteroids, cyclosporin, tacrolimus and cyclophosphamide.

**Conclusions:** Hypogammaglobinemia is an important side effect of rituximab that places patients at risk of life-threatening infections and mortality. Despite the relative safety and efficacy of rituximab, clinicians should appreciate the potential risks and consider adoption of a basic immunologic workup, including regular monitor of immunoglobulin levels preceding and following rituximab administration. Future research is required to determine reliable immunophenotyping or biomarkers that can reliably determine risk of severe side effects or treatment success.

**References:**

1. Trautmann A, Boyer O, Hodson E, Bagga A, Gipson DS, Samuel S, Wetzels J, Alhasan K, Banerjee S, Bhimma R, Bonilla-Felix M. IPNA clinical practice recommendations for the diagnosis and management of children with steroid-sensitive nephrotic syndrome. Pediatric nephrology. 2023 Mar;38(3):877–919.
2. Roberts DM, Jones RB, Smith RM, Alberici F, Kumaratne DS, Burns S, Jayne DR. Rituximab-associated hypogammaglobulinemia: incidence, predictors and outcomes in patients with multi-system autoimmune disease. Journal of autoimmunity. 2015 Feb 1;57:60–5.

## 98 Varicella zoster virus serology in pediatric patients treated with biologic therapies

### Bianca Pozzebon^1^, Maria Plesa^1^, Samar Elzein^1^, Ciriaco A. Piccirillo^1^, Bruce Mazer^1,2^, Christine McCusker^1,2^

#### ^1^Research Institute of the McGill University Health Centre, Montreal, QC; ^2^Pediatric Allergy, Immunology and Dermatology, Montreal Children’s Hospital, Montreal, QC

##### **Correspondence:** Bianca Pozzebon

*Allergy, Asthma & Clinical Immunology* 2025, **21(Suppl 1)**:98

**Background:** Varicella-zoster virus (VZV) is a highly infectious virus and the causative agent of chickenpox during primary infection, and shingles following reactivation, often in immunocompromised hosts [1]. There are 2 types of vaccines available for VZV; a live attenuated contraindicated for immunocompromised individuals [1] and a particulate vaccine indicated for the elderly and immunocompromised adults [2].

The vaccination status and the presence of seroconversion to VZV is not routinely evaluated in children undergoing biologic treatment [3]. Yet, these patients may have increased susceptibility to VZV infections. This study aims to establish VZV serology in pediatric patients at the start of their biologic therapies and follow VZV antibody levels during and after completion of biologics. The patient cohort is part of the Centre Transdisciplinaire de Thérapies Biologiques (CTTB) biobank and registry.

**Methods**: Vaccination status and past serology of the patients were compiled from the CTTB registry. An in-house enzyme-linked immunosorbent assay (ELISA) for anti-VZV antibody quantification was developed using Oka (vaccine) strain and recombinant (wild type WT) envelope glycoprotein E (gE) antigens. Serum from pediatric patients undergoing Rituximab or Infliximab therapy were/will be analyzed at baseline and 6-months, 1-year and 2-years post start of biological therapy.

**Results:** Eighteen patients were selected, 7 treated with Rituximab and 11 with Infliximab. Using Oka antigen, 5 patients (28%) were seronegative for VZV; 3/5 of which were not vaccinated and 2/5 received one VZV vaccination. Five different patients (28%) were seronegative to WT virus; 2/5 were never vaccinated, 2/5 had one vaccine and 1/5 had history of both natural infection and vaccination.

**Conclusions:** Pediatric patients beginning biologic treatment may be at risk of VZV infection as biologic therapies reduce the ability of patients to respond to infections. Our data highlight the importance of assessing seropositivity in patients and to consider the particulate VZV vaccine (Shingrix®) for these immunocompromised individuals.

**References:**

1. Papaloukas O, Giannouli G, Papaevangelou V. Successes and challenges in varicella vaccine. Ther Adv Vaccines. 2014;2(2):39–55.
2. Losa L, Antonazzo IC, Di Martino G, Mazzaglia G, Tafuri S, Mantovani LG, Ferrara P. Immunogenicity of Recombinant Zoster Vaccine: A Systematic Review, Meta-Analysis, and Meta-Regression. Vaccines. 2024;12(5):527.
3. Gershon AA, Breuer J, Cohen JI, Cohrs RJ, Gershon MD, Gilden D, et al. Varicella zoster virus infection. Nat Rev Dis Primers. 2015;1:15,016.

## 99 Chromatin landscape of CpG-induced regulatory B cells is highly dependent on originating cell subset

### Eisha A. Ahmed^1^, Nicholas Vonniessen^1^, Wei Zhao^1^, Bruce D. Mazer^1,2^

#### ^1^Research Institute of the McGill University Health Centre, Montreal, QC; ^2^Division of Allergy and Clinical Immunology, Department of Pediatrics, Montreal Children’s Hospital, Montreal, QC

##### **Correspondence:** Eisha A. Ahmed

*Allergy, Asthma & Clinical Immunology* 2025, **21(Suppl 1)**:99

**Background:** Regulatory B-cells (Bregs) are a heterogeneous cell subset of growing interest with the capacity to supress inflammatory responses. The most studied class of Bregs are those defined by the expression of interleukin-10 (IL-10), however B-cell subsets which express IL-10 and their mechanism of IL-10 induction remains unclear. Toll-like receptor activation is known to induce IL-10 expression in both mouse and human B-cells, with CpG – a TLR9 agonist – commonly used to induce Bregs in vitro.

**Methods**: In this study, we use CpG to induce IL-10 expression in sorted primary B cell subsets from naïve IL-10-eGFP reporter mice: follicular B (FOB) cells, marginal zone B (MZB) cells, and peritoneum-derived B1 cells. After 48 h of culture, B cells subsets were sorted a second time based on GFP expression, and chromatin landscape assessed via ATAC sequencing. Finally, we performed differential peak analysis and motif analysis to understand the differences in the epigenetic landscape that may underlie the capacity to express IL-10.

**Results:** Chromatin accessibility differs substantially across B cell subsets based on their originating population. MZB-derived cells exhibited more open chromatin at the *il10* locus compared to FOB-derived subsets, which may partially explain differences in their respective capacity to express IL-10 following CpG stimulation. We also observed greater chromatin accessibility globally in GFPhi compared to GFPlo subsets originating from peritoneal B1 cells. Differences between GFPlo and GFPhi populations within MZB and FOB subsets were more subtle, suggesting other mechanisms have a greater role in the heterogeneity in IL-10 expression within these cells. In contrast, B1-derived GFPhi cells had significantly more open chromatin at the *il10* locus, which may contribute to their robust IL-10-expression following stimulation.

**Conclusions:** This work highlights the necessity of deconvoluting heterogeneous cell responses to better understand mechanisms of gene regulation due to the significant differences between lymphoid and circulating B-cell populations.

## 100 Modeling the temporal decay of anti-N IgG following a COVID-19 infection

### Elsa Sakr^1^, Tanya Murphy^2^, Xun Zhang^3^, Danbing Ke^1^, Maria Plesa^1^, Bruce D. Mazer^1,4^

#### ^1^Translational Research in Respiratory Diseases, Meakins-Christie Laboratories, Research Institute of the McGill University Health Center, Montreal, QC; ^2^CITF Databank, School of Population and Global Health, McGill University, Montreal, QC; ^3^Centre for Outcomes Research and Evaluation, Research Institute of the McGill University Health Center, Montreal, QC; ^4^Department of Pediatrics, Faculty of Medicine and Health Sciences, McGill University, Montreal, QC

##### **Correspondence:** Elsa Sakr

*Allergy, Asthma & Clinical Immunology* 2025, **21(Suppl 1)**:100

**Background:** Anti-N IgG antibodies are specific antibodies produced in response to SARS-CoV-2 infection, targeting the virus’ nucleocapsid (N) protein. Infection-induced antibodies decay over time, as IgG is degraded and cleared. Understanding this decay is important for assessing long-term immunity conferred by infection. Studies have shown that anti-Spike and anti-Receptor-Binding-Domain IgG have a slower decay rate thus can be protective for a prolonged period of time. The dynamics of anti-N IgG antibodies, including their decay and half-life, are not well-characterized.

**Methods**: We studied a subset of 34 participants from our prospective cohort ‘The Living Lab Seroprevalence Study’ who provided serum samples at defined timepoints; anti-N IgG responses were assessed by chemiluminescent ELISA. Fluorescent detection above 1.0 SCO was considered positive for Anti-N IgG, while values below this cutoff were considered negative. We evaluated subjects with positive anti-N IgG (i.e. an infection) and assessed when anti-N antibodies were no longer detectable. A Cox survival regression model was employed to determine the half-life and average time to waning of these antibodies following an infection. Age, sex and repeated measures within participants were also accounted for in the model.

**Results:** Our analysis demonstrates a significant negative relationship between ‘days-post-infection’ and anti-N IgG levels, indicating a gradual decrease in anti-N IgG over time. The estimated half-life of anti-N IgG was approximately 86 days, with anti-N IgG levels dropping below the 1.0 SCO cutoff on average at around 228 days. The slope was derived from log-transformed anti-N IgG and found to be -0.0033, suggesting anti-N IgG levels decrease approximately 0.0033 SCO units per day in our cohort.

**Conclusions:** Our findings underscore the temporal dynamics of anti-N IgG antibodies following SARS-CoV-2 infection. The estimated half-life of 86 days provides insights into the longevity of the antibody protection conferred by infection.

## 101 Safeguarding the vulnerable: measles vaccination and seroprotection in immunocompromised pediatric patients on biologic therapies

### Eva Mamane^1^, Samar Elzein^1^, Maria Plesa^1^, Ewan Sullivan^1^, Ciriaco A. Piccirillo^1^, Bruce Mazer^2^, Christine McCusker^2^

#### ^1^RI-MUHC, Montreal, QC; ^2^Department of Paediatrics, Division of Allergy and Immunology, Montreal Children’s Hospital, Montreal, QC

##### **Correspondence:** Eva Mamane

*Allergy, Asthma & Clinical Immunology* 2025, **21(Suppl 1)**:101

**Background:** Vaccination status and seroprotection against measles in immunocompromised pediatric patients undergoing biologic therapies is a critical concern. These children are at heightened risk for severe complications from measles due to compromised immune systems. Proper seroprotection for this disease is essential to safeguarding their health and preventing outbreaks in vulnerable populations.[1].

This study aims to establish seroprevalence in an immunocompromised pediatric cohort to identify patients unprotected against measles and determine if there is a correlation between biologic used, underlying disease, and seroprotection to measles infection. The cohort is derived from the established Centre Transdisciplinaire Therapeutique Biologique (CTTB) biobank and registry.

**Methods**: We examined the established CTTB biobank for patients receiving biologics. We evaluated vaccination status prior to start of therapies and examined whether measles serology was evaluated. Using banked serum samples, we are evaluating measles protection using serum antibody levels by ELISA and the sensitive Plaque Reduction Neutralization test (PRNT).

**Results:** The pediatric CTTB cohort (N = 141) are undergoing treatment with biologic therapies including infliximab, and Rituximab.

Of the 141 children, 84(69%) received both recommended doses of the vaccine, 24(20%) received only one dose and13(11%) patients were never vaccinated. Vaccination status was unknown for 20(16.5%) patients. Prior to start of biologic therapy, measles serology assessed in 78% of the cohort revealed that 89(81%) patients were seropositive, 5(4%) patients were in the equivocal range and, importantly, 16(15%) patients were seronegative. Studies are ongoing to determine if these children maintain protection while on treatment.

**Conclusions:** Childhood vaccine immunization is essential for patients receiving biologic therapies as these are vulnerable, immunocompromised patients. Our findings show significant seronegativity in this cohort. Given recent outbreaks of measles in Canada (77 cases from 2024), these patients are vulnerable to infection and high-risk of morbidity.[2, 3].

Establishing pretreatment protocols and ensuring vaccine coverage is essential to protect this vulnerable population going forward.

**References:**

1. Osman S, Crowcroft N. **Population immunity to measles in Canada using Canadian Health Measures survey data – A Canadian Immunization Research Network (CIRN) study.** *National Library of Medicine*. 2002
2. Ehresmann K, Crouch N. **An outbreak of measles among unvaccinated young adults and measles seroprevalence study: implications for measles outbreak control in adult populations.** *National Library of Medicine*. 2004
3. **Measles and Rubella Weekly Monitoring Reports**. *Government of Canada*. 2024

## 102 Canadian Inborn Errors of Immunity Registry (CIEINR) is a platform that empowers development of the next generation of evidence-based practitioners and scientists in Canada

### Tatiana Kalashnikova^1^, Taylor Mattison^2^, Hanna Huska^1^, Luis Murguia-Favela^1^, Eyal Grunebaum^3^, Sneha Suresh^4^, Bruce Ritchie^4^, Juthaporn Cowan^5^, Tamar Rubin^6^, Hugo Chapdelaine^7^, Kevin Lee^8^, Catherine Biggs^8^, Rae Brager^9^, Ashley V. Geerlinks^9^, Whitney Goulstone^10^, Beata Derfalvi^2^, Nicola Wright^1^

#### ^1^Alberta Children’s Hospital, University of Calgary, Calgary, AB; ^2^IWK Health Centre, Dalhousie University, Halifax, NS; ^3^The Hospital for Sick Children, University of Toronto, Toronto, ON; ^4^Stollery Children’s Hospital, University of Alberta, Edmonton, AB; ^5^The Ottawa Hospital Research Institute, Ottawa, ON; ^6^Children’s Hospital of Winnipeg, University of Manitoba, Winnipeg, MB; ^7^Montreal Clinical Research Institute, University of Montreal, Montreal, QC; ^8^BC Children’s Hospital, University of British Columbia, Vancouver, BC; ^9^Children’s Hospital of London Health Sciences Centre, Western University, London, ON; ^10^ImmUnity Canada, Vancouver, BC

##### **Correspondence:** Tatiana Kalashnikova

*Allergy, Asthma & Clinical Immunology* 2025, **21(Suppl 1)**:102

**Background:** Patients with inborn errors of immunity (IEI) present with a broad spectrum of clinical, immunological, and genetic features that provide substantial opportunities for conducting research studies.

We identified a significant gap in resources within this field supporting students, medical residents, and fellows in initiating research projects. Typically, trainees would need to apply for ethics board approval, build data collection platforms, and navigate data sharing agreements between study centers—processes that can be time-consuming and restrict opportunities for trainees to focus on research projects.

To address these challenges, we developed the Canadian Inborn Error of Immunity National Registry (CIEINR) on a REDCap eCRF platform as a resource to enhance the educational experience of trainees in the field of Clinical Immunology in Canada.

**Methods**: The study design and regulatory documents for CIEINR were developed by the Registry Steering Committee. CIEINR Regulatory package is approved by the Research Ethics Boards in Alberta, Ontario, Nova Scotia, Manitoba. Other provinces are in the process of ethics consideration.

Data collection framework is based at the University of Calgary REDCap system enabling high-quality data collection, data sharing, and collaborative research.

**Results:** The National Canadian IEI Registry has been developed, and the patient recruitment phase has begun. Clinical and research trainees are encouraged to propose and execute sub-studies within the platform framework, addressing genotype–phenotype correlations, treatment outcomes, epidemiological trends, artificial intelligence implementation, quality improvement projects.

This platform supports trainees with hands-on experience in study design, patient recruitment, data collection, and analysis, enabling them to understand the landscape of IEIs in Canada, including diagnostic and management strategies.

**Conclusions:** By combining comprehensive data collection with educational opportunities, the CIEINR promotes both clinical research and the development of the next generation of researchers. Through this process, trainees will acquire analytical skills that are essential for healthcare professionals and ultimately enrich research on IEI.

**References:**

1. Condino-Neto A, Sullivan KE. The relevance of primary immunodeficiency registries on a global perspective. J Allergy Clin Immunol. 2021; 148(5):1170–1.

## 103 Creation of a database of pathogenic founder variants causing inborn errors of immunity with the aim of developing a platform for precision gene therapies

### Hanna Huska^1^, Robyn Loves^2^, Eyal Grunebaum^2,3^, Pierre Billon^4,5,6^, Ashish Marwaha^7^, Nicola Wright^1^

#### ^1^Department of Pediatrics, University of Calgary, Calgary, AB; ^2^Department of Immunology, University of Toronto, Toronto, ON; ^3^Developmental and Stem Cell Biology Program, the Hospital for Sick Children, Toronto, ON; ^4^Department of Biochemistry and Molecular Biology, University of Calgary, Calgary, AB; ^5^Robson DNA Science Center, Calgary, AB; ^6^Charbonneau Cancer Institute, Calgary, AB; ^7^Department of Medical Genetics, University of Calgary, Calgary, AB

##### **Correspondence:** Hanna Huska

*Allergy, Asthma & Clinical Immunology* 2025, **21(Suppl 1)**:103

**Background:** Inborn errors of immunity (IEI) are predominantly single gene germline variants that represent a significant health burden, particularly in populations with a high prevalence of founder variants. Many pathogenic variants are amendable to gene therapy, which for many patients has proven safer than allogenic hematopoietic stem cell transplantation by mitigating the requirement for a donor, risks of graft versus host disease, and requiring lower intensity conditioning and immunosuppression [1]. IEI caused by the CD3D founder variant and NCF1 hotspot provide examples of pathogenic variants amendable to base and prime editing respectively [2,3]. Our primary objective was to compile a comprehensive database of pathogenic founder variants causing IEI that could be viewed and updated in real time and used to identify the best correction strategies for additional candidate diseases.

**Methods**: Using the 2022 classification of IEI from the International Union of Immunological Societies Expert Committee (IUIS) [4] as reference, each disease was researched using Online Mendelian Inheritance in Man (OMIM), followed by a literature search using PubMed and other online resources, then classified using Franklin by Genoox [https://franklin.genoox.com] to ensure consistency.

**Results:** We compiled pathogenic founder variants causing IEI then reviewed each variant to determine the best candidates for gene correction including gene addition, base editing, and prime editing. Candidate founder variants were evaluated based on frequency, whether correction of the pathogenic variant in hematopoietic stem cells would provide clinical benefits, and the effects of the pathogenic variant on protein function (loss of function, gain of function, or neo-function).

**Conclusions:** Compiling a database of pathogenic variants resulting in IEI enables assessment of the potential for life saving disease specific gene therapies. Our database will guide prioritization of candidate IEI amendable to gene therapy and decision-making regarding the most appropriate gene correction strategies for specific founder variants.

**References:**

1. Grunebaum E, Booth C, Cuvelier GDE, Loves R, Aiuti A, Kohn DB. **Updated Management Guidelines for Adenosine Deaminase Deficiency.** J Allergy Clin Immunol Pract. 2023; **11(6)**:1665–75.
2. McAuley GE, Yiu G, Chang PC, Newby GA, Campo-Fernandez B, Fitz-Gibbon ST, et al. **Human T cell generation is restored in CD3δ severe combined immunodeficiency through adenine base editing.** Cell J. 2023; **186**:1398–1416.
3. Heath JM, Jacob Stuart Orenstein, Tedeschi JG, Ng A, Collier MD, Kushakji J, et al. **Prime Editing efficiently and precisely corrects causative mutation in chronic granulomatous disease, restoring myeloid function: Toward development of a prime edited autologous hematopoietic stem cell therapy**. Blood. 2023; **142**:7129.
4. Bousfiha A, Moundir A, Tangye SG, Picard C, Jeddane L, Al-Herz W, et al**. The 2022 Update of IUIS Phenotypical Classification for Human Inborn Errors of Immunity.** J Clin Immunol. 2022; **42(7)**:1508–20.

## 104 Efficacy and safety of amlitelimab maintenance/withdrawal in patients with moderate to severe atopic dermatitis: 52-week results from STREAM-AD

### Kim Papp^1^, Stephan Weidinger^2^, Andrew Blauvelt^3^, Adam Reich^4^, Chih-Hung Lee^5^, Margitta Worm^6^, Charles Lynde^7^, Yoko Kataoka^8^, Peter Foley^9^, Xiaodan Wei^10^, Anne-Catherine Solente^11^, Christine Weber^12^, Fabrice Hurbin^13^, Natalie Rynkiewicz^14^, Karl Yen^15^, John T. O’Malley^16^, Charlotte Bernigaud^12^

#### ^1^Department of Dermatology, Alliance Clinical Trials, Probity Medical Research, and University of Toronto, Waterloo, Toronto, ON; ^2^Department of Dermatology and Allergy, University Hospital Schleswig–Holstein, Kiel, Germany; ^3^Blauvelt Consulting, LLC Lake, Oswego, OR, USA; ^4^Department of Dermatology, University of Rzeszow, Rzeszow, Poland; ^5^Department of Dermatology, Kaohsiung Chang Gung Memorial Hospital, Kaohsiung, Taiwan; ^6^Department of Dermatology and Allergology, University Medical Center Berlin, Berlin, Germany; ^7^Department of Medicine, University of Toronto and Probity Medical Research, Markham, ON; ^8^Department of dermatology Osaka Habikino Medical Center, Habikino, Japan; ^9^Department of Dermatology, Skin Health Institute, Carlton, VIC, Australia; ^10^Department of B&P Immunology and Inflammation, Sanofi, Bridgewater, NJ, USA; ^11^Department of B&P Immunology and Inflammation, Sanofi, Paris, France; ^12^Department of Inflammation Dev Franchise, Sanofi, Paris, France; ^13^Department of Pharmacokinetic-Dynamics and Metabolism, Sanofi, Montpellier, France; ^14^Department of Translational Precision Medicine Immunology & Inflammation, Sanofi, Cambridge, United Kingdom; ^15^Department of Immuno Inflammation Dev Franchise, Sanofi, Rotkreuz, Switzerland; ^16^Department of Immunology and Inflammation GPT Amlitelimab, Sanofi, Cambridge, MA, USA

##### **Correspondence:** Kim Papp

*Allergy, Asthma & Clinical Immunology* 2025, **21(Suppl 1)**:104

**Background:** Amlitelimab is an OX40 ligand (OX40L) monoclonal antibody inhibiting OX40L-OX40 interactions. Here, we present data from the 28-week amlitelimab maintenance/withdrawal period (Part 2) of the Phase 2b (STREAM-AD, NCT05131477) dose-ranging trial in adults with moderate-to-severe atopic dermatitis (AD).

**Methods**: STREAM-AD Part 2 included clinical responders from Part 1, defined as participants achieving Eczema Area and Severity Index (EASI)-75 and/or Investigator Global Assessment (IGA) 0/1 at Week 24. Of 390 participants enrolled in Part 1, 190 entered Part 2. Participants were re-randomized 3:1 to withdraw treatment (replaced by placebo) or continue pre-Week 24 subcutaneous once-every-four weeks (Q4W) dose (250 mg with 500 mg loading dose [LD], n = 34 [treatment withdrawal]/n = 13 [continuing]; 250 mg, n = 28/n = 12; 125 mg, n = 33/n = 12; 62.5 mg, n = 35/n = 7; placebo/placebo, n = 16), and were followed to Week 52 for efficacy (28 weeks observation period, last dose in Part 1 at Week 20). Endpoints included maintenance of EASI-75 and/or IGA 0/1 response, and the percentage of patients with EASI-75 and IGA 0/1 response at Week 52. Statistical analysis was conducted by imputing endpoints, as non-responder after rescue medication use (NRI).

**Results:** Among clinical responders at Week 24, EASI-75 and/or IGA 0/1 response was maintained at Week 52 in 59%, 63%, 55%, and 66% of participants withdrawn from Q4W dose of 250 mg with LD, 250 mg, 125 mg, and 62.5 mg, respectively compared to 69%, 67%, 75%, 71% for those continuing amlitelimab. Maintenance of EASI-75 and IGA 0/1 at Week 52 was observed in 62% and 57% of clinical responders withdrawn from treatment in the pooled dosing group, respectively versus 69% and 72% in those continuing amlitelimab. AD-related biomarkers remained suppressed over 28 weeks. The safety profile remained generally consistent with Part 1 without new concerns identified in Part 2.

**Conclusions:** Maintenance of clinical responses were demonstrated for 28 weeks in the majority of patients, both on- and off- amlitelimab.

## 105 Quality improvement project to reduce the frequency of bloodwork in pediatric humoral immunodeficiency patients receiving monthly immunoglobulin replacement therapy

### Kristin M. Hunt, Abdulrahman Abdullah Al Ghamdi, Ashna Asim, Azhar Al Shaqaq, Narges Baluch, Yousef Alabdeen, Vy Kim

#### The Hospital for Sick Children, Toronto, ON

##### **Correspondence:** Kristin M. Hunt

*Allergy, Asthma & Clinical Immunology* 2025, **21(Suppl 1)**:105

**Background:** Pediatric patients with humoral immunodeficiencies are commonly treated with monthly intravenous immunoglobulin (IVIG) to protect them against recurrent infections. The current practice at Sickkids is that patients undergo “routine” bloodwork at each IVIG visit. However, there is limited evidence about how to best monitor these patients and at what frequency. An internal audit of 10 random IVIG patients demonstrated that 89% of visits had at least one abnormal lab value but only 3.8% of these resulted in a change in the management plan. This highlights that frequent blood sampling can be harmful, potentially leading to additional testing and contributing to escalating costs of healthcare.

**Methods**: Using a Plan-Do-Study-Act (PDSA) quality improvement methodology, we aimed to decrease the frequency of bloodwork drawn for at least 90% of patients with predominantly humoral deficiency who receive IVIG at our centre from every 4 weeks to every 12 weeks by January 2025. Therapy plans were updated accordingly in the electronic health system. Additional interventions included intra-departmental discussions, emails and staff meeting updates to medical day centre nurses, and educational handouts to patients and their families.

**Results:** From January to May 2024, the median time between routine bloodwork was 11.5 weeks. Only 13 (62%) of 21 eligible patients were deemed appropriate by their immunology physician for q12week bloodwork. There was an average reduction of 30 labs and $218.38 per patient over the first 6 months. There was no significant difference in the length of the IVIG visit when bloodwork was not drawn and no additional pokes outside the IV insertion with or without bloodwork. Of the IVIG visits with bloodwork, 88% had ≥ 1 lab abnormality but only 3.4% of these resulted in a change in management plan.

**Conclusions:** Reduced bloodwork frequency did not have a negative impact on patient care and saved healthcare resources.

## 106 Thymic stromal lymphopoietin secretion and trafficking in airway epithelial cells in response to panel of common allergen extracts

### Marc Duchesne, Luke Gerla, Paige Lacy

#### University of Alberta, Edmonton, AB

##### **Correspondence:** Marc Duchesne

*Allergy, Asthma & Clinical Immunology* 2025, **21(Suppl 1)**:106

**Background:** Airway epithelial cells (AECs) maintain lung immunity by releasing alarmin cytokines, including thymic stromal lymphopoietin (TSLP), a cytokine emerging as a key mediator in asthma. However, there is a fundamental lack of understanding of how allergens may affect the secretion of TSLP by AECs. Previous research suggests that cockroach extract (CE) induces TSLP release directly from AECs. We hypothesize that multiple allergens induce increased TSLP release.

**Methods**: Immortalized (BEAS-2B), normal (NHBE), and asthmatic bronchial epithelial (AHBE) cells were used to examine AECs TSLP trafficking in response to allergen extracts. AECs were stimulated with cockroach extract (CE), house dust mites (HDM), birch pollen extract (BPE), timothy grass extract (TGE), cat epithelia extract (CEE), hog epithelia extract (HEE), chicken feather/epithelia extract (CFE) or poly I:C for 0–24 h and fixed with 4% PFA for immunolabelled microscopy. In total, 10 cytokines were investigated following CE stimulation through immunofluorescence (IF) analysis: IL‑1β, ‑4, ‑6, -8, -13, -17, -25, -33, TNF-α, and TSLP. Protein synthesis (verracurin A) and transcriptional inhibitors (actinomycin D) were applied during extract stimulation. Coverslips were analyzed using a high-resolution Zeiss Elyra microscope and Volocity software V7. Supernatant cytokine levels were quantified with MSD kits.

**Results:**: Allergen-stimulated AECs showed quantifiable increases in TSLP, TNF-α, and IL-33, with TSLP having the highest levels (*p* < 0.01). The addition of verrucarin A or actinomycin D reverted TSLP IF to basal levels in CE stimulation, suggesting de novo synthesis of TSLP. Findings in BEAS-2Bs were confirmed in NHBEs and AHBEs.

**Conclusions:** Our results show for the first time the intracellular localization and subsequent release of TSLP in AECs during stimulation by multiple allergens. These findings are crucial for expanding our understanding of allergen-induced TSLP release in AECs.

## 107 Dupilumab is efficacious in children (aged 1 to < 12 years) with eosinophilic esophagitis regardless of prior history of type 2 comorbidities

### Jonathan M. Spergel^1^, Evan S. Dellon^2^, Changming Xia^3^, Tiffany Pela^4^, Bram P. Raphael^3^, Juby A. Jacob-Nara^4^, Amr Radwan^3^

#### ^1^Division of Allergy and Immunology, Children’s Hospital of Philadelphia, Philadelphia, PA, USA; ^2^Center for Esophageal Diseases and Swallowing, Division of Gastroenterology and Hepatology, University of North Carolina School of Medicine, Chapel Hill, NC, USA; ^3^Regeneron Pharmaceuticals Inc., Tarrytown, NY, USA; ^4^Sanofi, Bridgewater, NJ, USA

##### **Correspondence:** Jonathan M. Spergel

*Allergy, Asthma & Clinical Immunology* 2025, **21(Suppl 1)**:107

**Background:** Eosinophilic esophagitis (EoE) is a chronic, progressive, type 2 inflammatory disease with substantial impact on quality of life. Type 2 comorbidities are common in patients with EoE. Dupilumab is approved for the treatment of EoE for patients aged ≥ 12 years, weighing ≥ 40 kg, in Canada, and patients aged ≥ 1 year, weighing ≥ 15 kg in the US. This post hoc analysis of data from the phase 3 EoE KIDS study (NCT04394351) assessed dupilumab’s efficacy in children aged 1– < 12 years with EoE according to prior history of common comorbidities.

**Methods**: This analysis focused on patients randomized to a weight-tiered, higher-exposure dupilumab regimen or placebo for 16 weeks. Endpoints included proportions achieving peak intraepithelial eosinophil count ≤ 6, < 15, and ≤ 1 eosinophils/high-powered field (eos/hpf), and mean changes in Endoscopic Reference Score (EREFS), Histologic Scoring System (HSS) grade/stage scores, and Pediatric EoE Sign/Symptom Questionnaire-Caregiver version; all stratified by prior history of atopic dermatitis (AD), asthma, and allergic rhinitis (AR).

**Results:** Almost all patients (97.2%) had a type 2 comorbidity; 43 had AD (dupilumab/placebo, n = 21/22), 39 had asthma (n = 24/15), and 51 had AR (n = 29/22). At Week 16, dupilumab led to greater proportions achieving ≤ 6 eos/hpf vs placebo, irrespective of comorbidities (rate difference versus placebo [95% CI]: AD: yes 71.4% [52.1–90.8], no 54.2% [25.8–82.6]; asthma: yes 55.8% [32.7–79.0], no 76.9% [54.0–99.8]; AR: yes 64.4% [45.5–83.4], no 62.5 [29.0–96.1]). Dupilumab also improved proportions achieving < 15 and ≤ 1 eos/hpf, and improved EREFS and HSS grade/stage scores vs placebo in all subgroups. Dupilumab generally reduced the proportion of days with ≥ 1 EoE sign vs placebo at Week 16, although this varied across comorbidities.

**Conclusions:** Dupilumab improved features of EoE versus placebo, in children aged 1- < 12 years with or without past medical history of common type 2 comorbidities (AD, asthma, and AR).

## 108 Dupilumab is efficacious up to 52 weeks in patients with eosinophilic esophagitis irrespective of prior elimination diet or history of food allergy

### Jonathan M. Spergel^1^, Marc E. Rothenberg^2^, Antonella Cianferoni^3^, Changming Xia^4^, Margee Louisias^5^, Allen Radin^4^

#### ^1^Division of Allergy and Immunology, Children’s Hospital of Philadelphia, Philadelphia, PA, USA; ^2^Division of Allergy and Immunology, Department of Pediatrics, Cincinnati Children’s Hospital Medical Center and University of Cincinnati College of Medicine, Cincinnati, OH, USA; ^3^Department of Pediatrics, Perelman School of Medicine at the University of Pennsylvania, Philadelphia, PA, USA; ^4^Regeneron Pharmaceuticals Inc., Tarrytown, NY, USA; ^5^Sanofi, Bridgewater, NJ, USA

##### **Correspondence:** Jonathan M. Spergel

*Allergy, Asthma & Clinical Immunology* 2025, **21(Suppl 1)**:108

**Background:** Eosinophilic esophagitis (EoE) is a chronic, progressive, type-2 inflammatory disease, with high food allergy comorbidity. Elimination diets can improve symptoms, but maintenance can be challenging. Dupilumab is approved for the treatment of EoE in patients ≥ 12 years, weighing ≥ 40 kg, in Canada, and patients aged ≥ 1 year, weighing ≥ 15 kg, in the US. Dupilumab demonstrated improvements in key outcome measures in patients with EoE with/without a history of food allergy (self-declared, unverified) or food elimination diet at Week 24 versus placebo in Part B of the 3-part LIBERTY EoE TREET study (NCT03633617). This subgroup analysis assessed the efficacy of dupilumab versus placebo at Week 52 (the end of Part C).

**Methods**: Eligible patients completing Part B entered Part C and received 28 weeks of dupilumab 300 mg weekly. Endpoints assessed were proportion of patients achieving peak intraepithelial eosinophil count ≤ 6 eosinophils per high-powered field (eos/hpf), and absolute change from baseline in Dysphagia Symptom Questionnaire (DSQ) score, Endoscopic Reference Score (EREFS) total score, and EoE-Histologic Scoring System (HSS) stage/grade scores.

**Results:** In patients who continued dupilumab, the proportion achieving ≤ 6 eos/hpf (with/without elimination diet [standard error] improved: 87%[0.06]/82%[0.07]; with/without prior food allergy: 77%[0.07]/93%[0.05]), DSQ score (absolute mean change [standard deviation]: –31.1[17.0]/–29.1[12.9]; -32.7[14.2]/-27.7[16.5]), and EREFS total score (absolute mean change [standard deviation]: –5.6[2.5]/–5.1[3.2]; –5.0[2.8]/–5.8[2.9]). The proportion achieving ≤ 6 eos/hpf (67%[0.10]/69%[0.12]; 65%[0.11]/71%[0.11]), DSQ score (–28.4[9.9]/–25.4[14.2]; -26.1[10.8]/-28.8[12.8]), and EREFS total score (–6.7[3.8]/–5.3[3.2]; –6.2[4.1]/–6.1[3.1]) also improved in those patients who switched to dupilumab from placebo. HSS grade/stage scores were improved or maintained in patients continuing dupilumab and improved in patients switching to dupilumab from placebo.

**Conclusions:** Dupilumab led to sustained improvements in histologic, symptomatic, and endoscopic aspects of EoE in adults and adolescents up to 52 weeks, regardless of prior elimination diet or food allergy.

## Urticaria/Angioedema

## 109 Assessment of the quality of life in children with chronic urticaria using the children’s dermatology life quality questionnaire index (CDLQI)

### Catherine K. Zhu^1^, Connor Prosty^1^, Sofianne Gabrielli^1^, Michelle Le^2^, Elena Netchiporouk^2^, Xun Zhang^3^, Barbara Miedzybrodski^2^, Michael Fein^4^, Moshe Ben-Shoshan^5^

#### ^1^Faculty of Medicine, McGill University, Montreal, QC; ^2^Division of Dermatology, McGill University Health Centre, Montreal, QC; ^3^Centre for Outcome Research and Evaluation, Research Institute of the McGill University Health Centre, Montreal, QC; ^4^Division of Allergy and Clinical Immunology, Department of Medicine, McGill University Health Centre, Montreal, QC; ^5^Division of Pediatric Allergy and Clinical Immunology, Department of Pediatrics, Montreal, QC

##### **Correspondence:** Catherine K. Zhu

*Allergy, Asthma & Clinical Immunology* 2025, **21(Suppl 1)**:109

**Background:** Chronic urticaria (CU) is a mast cell-driven disease with adverse effects on a child’s quality of life (QoL). We aimed to assess the impact of CU on children’s QoL by Children’s Dermatology Life Quality Index (CDLQI).

**Methods**: Children (4–16 years old) with CU were recruited. Patients were administered standardized questionnaires on demographics, management of CU, and comorbidities. Chart review was performed to assess laboratory characteristics. Recruited children completed the CDLQI at study entry (validated for children 4–16 years old). Multivariate logistic regression was performed to identify factors associated with better (*i.e., CDLQI* ≤ *5*) and worse QoL (*i.e., CDLQI* > *5*) based on established CDLQI cut-offs.

**Results:** 74 children with CU (median age = 10.0, 58% male) were recruited. Among all children, 39 (52.7%) had isolated chronic spontaneous urticaria (CSU), 21 (27.0%) had isolated chronic inducible urticaria (CIndU), and 14 (16.2%) had concomitant CSU and CIndU. Based on their CDLQI scores, CU had no effect on the QoL for 30 children (40.5%), a small effect in 29 (39.2%), moderate effect in 11 (14.9%), very large effect in 3 (4.1%), and extremely large effect in one child (1.4%). On multivariate logistic regression, older age at symptoms onset [adjusted Odds Ratio (aOR) = 1.05, 95% confidence interval (95% CI) = 1.02–1.09], elevated CRP (> 5 mg/L) (aOR = 1.49, 95%CI = 1.04–2.12) and history of atopic dermatitis [aOR = 1.45, 95%CI = 1.96–2.17] were associated with worse QoL (CDLQI > 5) when adjusting for sex. Subgroup analysis using age at enrollment suggested that children aged 4–10 showed no significant association between age and QoL, while adolescents aged 11–16 showed that older age was associated with worse QoL [aOR = 1.12, 95%CI = 1.03, 1.22] when adjusting for sex. No significant association with CDLQI were found for other demographic/clinical characteristics.

**Conclusions:** Our findings suggest that patient characteristics such as older age at onset of CU symptoms, elevated CRP, and history of atopic dermatitis, are associated with worse QoL in children with CU.

## 110 Comparing children and adults with chronic spontaneous urticaria: is there a difference?

### Lauren Perlman^1^, Justin Sacksner^1^, Elena Netchiporouk^2^, Michael Fein^3^, John Sampalis^4^, Xun Zhang^5^, Moshe Ben-Shoshan^1^

#### ^1^Division of Pediatric Allergy, Immunology and Dermatology, Montreal Children’s Hospital, McGill University, Montreal, QC; ^2^Division of Dermatology, McGill University, Montreal, QC; ^3^Division of Allergy and Clinical Immunology, McGill University Health Centre, Montreal, QC; ^4^Division of Surgical Research, Surgical Epidemiology, McGill University, Montreal, QC; ^5^Centre for Outcome Research and Evaluation, Research Institute of McGill University Health Centre, Montreal, QC

##### **Correspondence:** Lauren Perlman

*Allergy, Asthma & Clinical Immunology* 2025, **21(Suppl 1)**:110

**Background:** Data comparing children and adults with chronic spontaneous urticaria (CSU) are sparse worldwide. We aimed to assess differences in demographics, comorbidities, treatment, and laboratory markers between these two populations.

**Methods**: We recruited children (< 18 years) and adults with CSU from the Montreal Chronic Urticaria Registry. Patients completed standardized questionnaires on demographics, known atopic and autoimmune conditions, and management of CSU. Using R v4.2.0, we compared the prevalence of these aforementioned factors using chi-squared testing. Using t-testing, we assessed the difference in continuous variables such as laboratory markers and age. Significance was set at 0.05.

**Results:** 56 children and 31 adults with CSU were recruited from 2014–2024. 23 (41.1%) children and 11 (35.5%) adults were male, with median ages being 11.1 (IQR 7.8) and 38.4 (15.9), respectively. The most prevalent atopic conditions were asthma (6 children (10.7%) and 8 adults (25.8%) and atopic dermatitis (6 (10.7%), and 10 (32.3%)). Of all, 8 children had known autoimmune diseases, with hypothyroidism being the most common (n = 4, 7.1%). Nearly all children and adults with CSU were treated with antihistamines (n = 49 (87.5%), n = 29 (93.5%)). Significantly more adults receive 4 doses of second-generation antihistamines compared to children (p = 0.01). Atopic dermatitis was significantly more prevalent amongst adults (p = 0.03) and their mean tryptase levels were significantly higher (p = 0.04).

**Conclusions:** Adults with CSU are more likely to present with comorbid atopic dermatitis and require 4 doses of second-generation antihistamines to treat their symptoms, indicating more poorly controlled forms of CSU compared to children.

## 111 Retrospective review of hereditary angioedema manifestation and management in pregnancy – preliminary findings in Manitoba

### Ming H. Bi, Chrystyna Kalicinsky

#### University of Manitoba, Winnipeg, MB

##### **Correspondence:** Ming H. Bi

*Allergy, Asthma & Clinical Immunology* 2025, **21(Suppl 1)**:111

**Background:** Hereditary Angioedema (HAE) is a rare genetic condition resulting in recurrent, potentially life threatening, angioedema. Pregnancy may complicate HAE management, with uncertain effects on angioedema frequency and severity, and limited approved treatments for pregnant and breastfeeding women. Prior studies report varying results regarding effects of pregnancy on HAE and whether HAE influences pregnancy outcomes.

**Methods**: Women > 18 years of age with HAE in Manitoba who experienced any pregnancies were screened between June 2022—May 2024 for inclusion as part of a multi-centre Canadian study. Records were reviewed for past medical history, baseline characteristics, pregnancy outcomes, HAE control and management; participants were interviewed for additional information if records were incomplete.

**Results:** Our cohort, comprised of 11 women, contributed 42 pregnancies in total. Mean age of participants was 48.9 years, with a range of 24–68 years. 6/11 women experienced at least 1 pregnancy after HAE diagnosis, with 16 pregnancies occurring after and 26 pregnancies occurring prior to HAE diagnosis. In comparing frequency of attacks between 1 year prior to pregnancy and during pregnancy and breastfeeding, 3/11 participants had no attacks in these periods, 2/11 women only experienced increases in attacks, and 6/11 women experienced variable changes in attack frequency. 6 women noted changes in attack locations during pregnancy or breastfeeding compared to 1 year prior. 4/6 women with pregnancies post-HAE diagnosis received C1-esterance inhibitor for either prophylaxis or treatment during pregnancy or breastfeeding; 1 of these 4 women received icatibant for treatment during pregnancy and 1 other individual for treatment during breastfeeding. 45.2% (n = 19) of pregnancies experienced complications, including but not limited to spontaneous abortion, stillbirth, pre-eclampsia, NICU admission, and hyperemesis gravidarum.

**Conclusions:** HAE attack frequency and characteristics were variable during pregnancy in our cohort. A minority of women received prophylaxis and treatment of attacks, likely because most pregnancies occurred prior to HAE diagnosis.

## 112 Investigation of measures of disease severity in children with chronic spontaneous urticaria in a prospective registry cohort

### Michael J. Aw^1^, Magan Solomon^1^, Gregory Shand^2^, Elena Netchiporouk^3^, John Samplis^4^, Michael Fein^5^, Moshe Ben-Shoshan^2^

#### ^1^Faculty of Medicine, Department of Internal Medicine, McGill University, Montreal, QC; ^2^Division of Allergy & Clinical Immunology, Montreal Children’s Hospital, McGill University, Montreal, QC; ^3^Division of Dermatology, McGill University, Montreal, QC; ^4^Division of Surgical research, Surgical Epidemiology, McGill University, Montreal, QC; ^5^Division of Adult Allergy & Clinical Immunology, McGill University, Montreal, QC

##### **Correspondence:** Michael J. Aw

*Allergy, Asthma & Clinical Immunology* 2025, **21(Suppl 1)**:112

**Background:** Chronic spontaneous urticaria (CSU) affects approximately 1% of the pediatric population(1). CSU is a heterogeneous disease and biochemical measures of disease severity, specifically for children, remain largely unknown. We aimed to characterize the clinical presentation of CSU in children and evaluate the association between biomarkers and disease severity.

**Methods**: Children (0–18 years) with CSU who presented to the Montreal Children’s Hospital and a Montreal community clinic between 2013 and 2022 were prospectively recruited. Patient demographic data and laboratory testing at study entry were collected. Disease activity was measured using the weekly Urticaria Activity Score (UAS7) at initial and follow up visits. Linear regression was performed for continuous variables, and t tests and chi-squared for between group comparisons using R 4.2.1(2).

**Results:** We included 336 eligible participants (48.5% male) for analysis, of which 33.6% had co-morbid atopy (asthma, eczema, food allergy, allergic rhinitis). The mean age of CSU onset was earlier in males (9.3 vs 13.0, p = 0.008). The mean first reported UAS7 score was 8 (0–16), of which 11.7% participants required anti-IgE treatment. There was an independent positive association between serum tryptase levels (β 0.58 95%CI 0.40–0.76, p = 0.001) and leukocytes (β 0.49 95%CI 0.24–0.74, p = 0.05) with higher UAS7 scores at study entry. The tryptase association was maintained when adjusted for sex but not age. No associations were identified between C-reactive protein, basophils or IgE levels and UAS7 scores. Furthermore, higher UAS7 scores (β 0.26 95%CI 0.18–0.35, p = 0.002), and tryptase levels (β 0.34 95%CI 0.17–0.51, p = 0.04) were independently positively associated with later age of CSU onset.

**Conclusions:** We have identified serum tryptase levels as a possible biomarker to estimate CSU disease activity. Furthermore, we report sex differences in disease onset and an increase in self-reported disease severity with later onset of CSU. This data contributes to our understanding of pediatric CSU.

**References:**

1. Sánchez-Borges M, Ansotegui IJ, Baiardini I, Bernstein J, Canonica GW, Ebisawa M, et al. The challenges of chronic urticaria part 1: Epidemiology, immunopathogenesis, comorbidities, quality of life, and management. World Allergy Organ J. 2021;14(6):100,533.
2. R development core team. R: A language and environmental for statistical computing. Vienna, Austria: R Foundation for Statistical Computing; 2010.

## 113 Use of HAE quality of life questionnaires and control tools by Canadian physicians

### Natasha Correa^1,2^, Stephen Betschel^1,2^

#### ^1^University of Toronto, Toronto, ON; ^2^St. Michael’s Hospital, Toronto, ON

##### **Correspondence:** Natasha Correa

*Allergy, Asthma & Clinical Immunology* 2025, **21(Suppl 1)**:113

**Background:** Hereditary angioedema (HAE) is caused by C1 esterase inhibitor deficiency. HAE manifests as episodes of angioedema resulting in quality of life impairment, morbidity and possible fatality. Guidelines recommend healthcare providers routinely assess quality of life in HAE patients using validated instruments to optimize HAE management. It is unknown whether Canadian physicians use HAE quality of life questionnaires or control tools in clinical practice and the barriers they face in doing so.

**Methods**: We conducted an online survey of HAE treating physicians in Canada. The survey was administered using REDCap between February to April 2024. Participants were recruited from clinical organizations that include physicians who treat HAE. Participants were asked how often they used HAE quality of life questionnaires or control tools, which ones they used and the barriers they experienced in using them.

**Results:** 43 physicians completed the survey. 18% never used HAE quality of life questionnaires or control tools, 13% used them occasionally, 13% used them half the time, 50% used them most of the time, and 5% used them all the time. The most frequently tool was the Angioedema Quality of Life Questionnaire (37%), followed by the Angioedema Control Test (30%), followed by the Hereditary Angioedema Activity Score (29%). The most frequently reported barriers were not knowing where to find these tools (29%), lack of time to use them (21%), and not finding them to be clinically helpful (11%).

**Conclusions:** Most HAE treating physicians in Canada do not always use HAE quality of life questionnaires and control tools in the management of patients with HAE. Inability to find these tools and lack of time were the most frequently cited barriers. Further studies are needed to investigate strategies to increase the use of HAE quality of life questionnaires and control tools in clinical practice.

## 114 Anti-thyroid peroxidase antibody (Anti-TPO antibodies) is higher in children with chronic urticaria who require omalizumab

### Zainab Alsaffar^1^, Roy Khalaf^2^, Abdulaziz S. Alrafiaah^3,4^, Sarah D. Mohamed^2^, Elena Netchiporouk^5^, Michael Fein^6^, John Sampalis^7^, Moshe Ben-Shoshan^3^

#### ^1^Department of Pediatrics, McGill University Health Centre, Montreal, QC; ^2^Faculty of Medicine and Health Science, McGill University, Montreal, QC, ^3^Department of Pediatric, Division of Allergy Immunology and Dermatology, Montreal, QC; ^4^Department of Pediatrics, College of Medicine, Majmaah university, Majmaah, 11,952, Saudi Arabia; ^5^Department of Dermatology, McGill University health centre, Montreal, QC; ^6^Department of Allergy and Immunology, McGill University Health Centre, Montreal, QC; ^7^Department of Surgery, McGill University Health Centre, Montreal, QC

##### **Correspondence:** Zainab Alsaffar, Abdulaziz S. Alrafiaah

*Allergy, Asthma & Clinical Immunology* 2025, **21(Suppl 1)**:114

**Background:** Chronic urticaria (CU) is characterized by occurrence of hives/angioedema or both, for a duration of at least six weeks. The association between anti-Thyroid peroxidase (anti-TPO) in CU and poor response to treatment with high dose (4 times the standard dose) of antihistamine was not explored in pediatric CSU literature.

**Methods**: This was a prospective cohort study over a 11-year period which included pediatric patients (0–18 years) with chronic urticaria who presented to allergy clinic at Montreal children hospital in Canada during period from 2013 to 2024. Data including patent’s demographics, comorbidities, urticaria activity score (UAS7), urticaria control test (UCT), laboratory findings including Anti-TPO, treatment regimens and treatment response were collected.

**Results:** During the study period, 183 patients with chronic urticaria were included, and 14 of them had elevated anti-TPO levels, with a prevalence of 7.6%.The median age for those patients was 17.0 (15.3–21.8) years and 28.6% of them were males. The elevated anti-TPO group had significantly higher mean anti-TPO levels compared to the non-elevated group (p < 0.001), with no difference in mean TSH levels (p = 0.81). Antihistamines were the most common treatment in both groups. Omalizumab use was significantly higher in the elevated anti-TPO group (28.6%) compared to the non-elevated group (5.3%) (p = 0.01). There was no significant difference in baseline UAS7 scores and UCT scores between the groups (p = 0.52)(p = 0.77) respectively.

**Conclusions:** The findings of this study suggested that anti-TPO positivity is associated with a higher likelihood of requiring Omalizumab in children with chronic urticaria, this finding aligning with observations in previous studies in adult populations with CSU.

## 115 Quality of life (QoL) improvements in hereditary angioedema (HAE) with STAR-0215; interim results from the phase 1b/2 ALPHA-STAR clinical trial

### Adil Adatia^1^, Theodora Cohen^2^, Christopher Morabito^2^, William H. Yang^3^

#### ^1^University of Alberta, Edmonton, AB; ^2^Astria Therapeutics, Boston, MA, USA; ^3^Ottawa Allergy Research Corporation, Ottawa, ON

##### **Correspondence:** Adil Adatia

*Allergy, Asthma & Clinical Immunology* 2025, **21(Suppl 1)**:115

**Background:** ALPHA-STAR (NCT05695248) is an ongoing phase 1b/2 clinical trial in patients with HAE, investigating safety and efficacy of STAR-0215, humanized monoclonal antibody inhibitor of plasma kallikrein with long-lasting activity enabled by a YTE-modified Fc domain.

**Methods**: Adults (n = 16) with HAE-C1INH-Type1/2, were recruited into 3 dose cohorts, Cohort 1: 450 mg (day 1); Cohort 2: 600 mg (day 1), 300 mg (day 84); Cohort 3: 600 mg (day 1 and day 28). Quality of life was assessed at baseline and monthly thereafter using Angioedema Quality of Life (AE-QoL) questionnaire, that has a minimal clinically important difference (MCID) of –6 points. This analysis focuses on available data (n = 15) for the first 84 days post treatment in ALPHA-STAR.

**Results:** The mean age (SD) of study participants was 46 (20) years, 56% were female; 88% had HAE C1INH-Type 1. No serious treatment emergent adverse events or treatment discontinuations were reported. By day 28, the total AE-QoL score improved by -20.9 (17.9) from mean (SD) baseline score of 37.5 (15.6); improvement was sustained -22.5 (17.5) on day 84, across all three cohorts combined. Improvements were achieved and persisted across all domains of the AE-QoL score; at day 84 functioning score improved by -28.4 (30.1), fatigue/mood improved by -21.4 (23.7), fear/shame score improved by -18.5 (20.1) and nutrition score improved by -18.8 (21.2). Within the first month, 80% of participants achieved clinically meaningful improvements (MCID) which was maintained through 84 days.

**Conclusions:** Following single or multiple doses of STAR-0215, participants in the ALPHA-STAR clinical trial experienced rapid and meaningful improvements in quality of life which were sustained for at least 84 days.

## 116 Higher omalizumab usage in children with concurrent chronic spontaneous and inducible urticaria compared to CSU alone or chronic inducible urticaria alone

### Abdulaziz S. Alrafiaah^1,2^, Roy Khalaf^3^, Carly Sillcox^1^, Sundus NoorSaeed^1,4^, Barbara Miedzybrodzki^5^, Elena Netchiporouk^6^, Moshe Ben-Shoshan^1^

#### ^1^Division of Pediatric Allergy and Clinical Immunology, Department of Pediatrics, McGill University Health Center, Montreal, QC; ^2^Division of Pediatric, College of Medicine, Majmaah University, Al Majma’ah, Saudi Arabia; ^3^Faculty of Medicine-McGill University, Montreal, QC; ^4^Department of Pediatric, King AbdulAziz University hospital, Jeddah, Saudi Arabia; ^5^Division of Pediatric Dermatology, McGill University Health Centre, Montreal Children’s Hospital, Montreal, QC; ^6^Division of Dermatology, McGill University Health Centre, Montreal, QC

##### **Correspondence:** Abdulaziz S. Alrafiaah

*Allergy, Asthma & Clinical Immunology* 2025, **21(Suppl 1)**:116

**Background:** Chronic spontaneous urticaria (CSU) presents as repetitive spontaneous hives and/or angioedema lasting for at least six weeks. In contrast chronic inducible urticaria (CIndU) is triggered by specific stimuli. This study aimed to characterize children who has concurrent CSU and CIndU, and to identify factors that distinguish them from children with CSU alone or CIndU alone.

**Methods**: This prospective cohort study was conducted over an 11-year period, from 2013 to 2024, at Montreal Children’s Hospital in Canada. It included pediatric patients aged 0–18 years with chronic urticaria.

**Results:** During the study period, 202 pediatric patients with chronic urticaria were included. Of these, 20 patients (9.9%) had both CSU and CIndU concurrently. Cold urticaria was the most common CIndU associated with CSU, affecting 11 patients (55%). The mean age of patients with concurrent CSU and CIndU was 6.2 years (5.0–11.8), with females comprising the majority at 12 patients (60%). Atopic dermatitis was the most prevalent atopic condition in this group, affecting 4 patients (20%). Regarding the timing of urticaria onset, 8 patients (42%) initially presented with CSU alone. Although children with concurrent CSU and CINDU had higher baseline UAS7 and UCT scores compared to those with CSU alone or CIndU alone, this difference was not statistically significant. Antihistamines were commonly used in most children with concurrent CSU and CIndU (16 patients, 80%). Omalizumab usage was significantly higher in children with concurrent CSU and CIndU (20%) compared to those with CSU alone (5.9%) and CINDU alone (0%), with p-values of 0.04 and 0.01, respectively.

**Conclusions:** Pediatric patients who have concomitant CSU and CIndU are more likely have severe disease that require more advanced treatment options like omalizumab as compared to children with CSU/ CIndU alone.

## 117 Efficacy and safety of oral deucrictibant, a bradykinin b2 receptor antagonist, in prophylaxis of HAE attacks: results of CHAPTER-1 phase 2 trial

### William H. Yang^1^, John Anderson^2^, Francesco Arcoleo^3^, Mauro Cancian^4^, Hugo Chapdelaine^5^, Niall Conlon^6^, Efrem Eren^7^, Mark Gompels^8^, Sofia Grigoriadou^9^, Maria D. Guarino^10^, Padmalal Gurugama^11^, Tamar Kinaciyan^12^, Markus Magerl^13,14^, Michael E. Manning^15^, Marcin Stobiecki^16^, Michael D. Tarzi^17^, Anna Valerieva^18^, H. James Wedner^19^, Andrea Zanichelli^20,21^, Rafael Crabbé^22^, Susan Mulders^23^, Minying Royston^24^, Li Zhu^24^, Jochen Knolle^25^, Anne Lesage^26^, Peng Lu^24^, Marc A. Riedl^27^, Emel Aygören-Pürsün^28^

#### ^1^University of Ottawa, Ottawa Allergy Research Corporation, Department of Medicine, Ottawa, ON; ^2^AllerVie Health, Clinical Research Center of Alabama, Birmingham, AL, USA; ^3^AOR Villa Sofia-Cervello, UOC di Patologia Clinica e Immunologia, Palermo, Italy; ^4^University Hospital of Padua, Department of Systems Medicine, Padua, Italy; ^5^Université de Montréal, CHU de Montréal, Montréal, QC; ^6^St. James’s Hospital and Trinity College, Wellcome Trust CRF, Dublin, Ireland; ^7^University Hospital Southampton NHS Foundation Trust, Southampton, United Kingdom; ^8^North Bristol NHS Trust, Bristol, United Kingdom; ^9^Barts Health NHS Trust, Department of Immunology, London, United Kingdom; ^10^Ospedale di Civitanova Marche, Civitanova Marche, Italy; ^11^Cambridge University Hospitals NHS Foundation Trust, Department of Clinical Immunology, Cambridge, United Kingdom; ^12^Medical University of Vienna, Department of Dermatology, Vienna, Austria; ^13^Charité – Universitätsmedizin Berlin, Institute of Allergology, Corporate Member of Freie Universität Berlin and Humboldt-Universität zu Berlin, Berlin, Germany; ^14^Fraunhofer Institute for Translational Medicine and Pharmacology ITMP, Immunology and Allergology, Berlin, Germany; ^15^Allergy, Asthma and Immunology Associates, Ltd., Scottsdale, AZ, USA; ^16^Jagiellonian University Medical College, Department of Clinical and Environmental Allergology, Krakow, Poland; ^17^University Hospitals Sussex NHS Foundation Trust, Department of Respiratory Medicine, Brighton, United Kingdom; ^18^University Hospital Alexandrovska, Department of Allergology, Clinic of Allergology, Medical University of Sofia, Sofia, Bulgaria, ^19^Washington University School of Medicine, Division of Allergy and Immunology, Department of Medicine, St. Louis, MO, USA; ^20^Universita degli Studi di Milano, Dipartimento di Scienze Biomediche per la Salute, Milan, Italy; ^21^I.R.C.C.S., Policlinico San Donato, Centro Angioedema, UO Medicina, Milan, Italy; ^22^RC Consultancy, Bassins, Switzerland; ^23^Mulders Clinical Consulting, Groesbeek, Netherlands; ^24^Pharvaris Inc., Lexington, MA, USA; ^25^JCK Consult, Frankfurt, Germany; ^26^GrayMatters Consulting, Schilde, Belgium; ^27^University of California San Diego, Division of Allergy and Immunology, La Jolla, CA, USA; ^28^University Hospital Frankfurt, Department for Children and Adolescents; Goethe University Frankfurt, Frankfurt, Germany

##### **Correspondence:** William H. Yang

*Allergy, Asthma & Clinical Immunology* 2025, **21(Suppl 1)**:117

**Background:** Hereditary angioedema (HAE) attacks are caused by excess bradykinin activating bradykinin B2 receptors. Deucrictibant is an orally-administered bradykinin B2 receptor antagonist under development for on-demand and prophylactic treatment of HAE attacks.

**Methods**: CHAPTER-1 (NCT05047185) is an ongoing 2-part Phase 2 study evaluating deucrictibant for long-term prophylaxis of HAE attacks. In part 1, participants received double-blind treatment with placebo or deucrictibant 20 mg/day or 40 mg/day (administered as immediate-release capsule formulation) for 12 weeks. Thirty-four participants were enrolled. In the ongoing part 2, participants may receive treatment with open-label deucrictibant 40 mg/day. Participants were aged ≥ 18 and ≤ 75 years, diagnosed with HAE-1/2, not receiving other prophylactic treatments at the time of screening, and experienced ≥ 3 attacks within the 3 months prior to screening or ≥ 2 attacks during screening (up to 8 weeks).

**Results:** In placebo-controlled part 1, the monthly attack rate (primary endpoint) was reduced by 84.5% (p = 0.0008) by deucrictibant 40 mg/day (least squares mean: 0.30; 95% CI: 0.11, 0.82) vs placebo (1.94; 1.31, 2.87). Deucrictibant 40 mg/day reduced the monthly rate of “moderate and severe” attacks by 92.3% and the rate of attacks treated with on-demand medication by 92.6%. At 12 weeks, ≥ 50%, ≥ 70%, and ≥ 90% reduction in attack rate from baseline was achieved in 9, 8, and 6 of 10 participants receiving deucrictibant 40 mg/day vs 2, 2, and 0 of 11 participants receiving placebo. Forty percent of participants receiving deucrictibant 40 mg/day vs 0% receiving placebo were attack-free. Both doses of deucrictibant were well-tolerated. Four mild treatment-related treatment-emergent adverse events (TEAEs) were reported by 4 participants: 1 receiving placebo, 2 deucrictibant 20 mg/day, and 1 deucrictibant 40 mg/day. There were no serious TEAEs, severe TEAEs, or TEAEs leading to treatment discontinuation.

**Conclusions:** CHAPTER-1 provides evidence on efficacy and safety of deucrictibant for the prevention of HAE attacks and supports its further development as a potential prophylactic therapy.

## 118 Long-term efficacy and safety of oral bradykinin B2 receptor antagonist deucrictibant in treatment of HAE attacks: RAPIDe-2 extension study results

### Hugo Chapdelaine^1^, Emel Aygören-Pürsün^2^, Laurence Bouillet^3^, Henriette Farkas^4^, Delphine Gobert^5^, Roman Hakl^6^, Ramon Lleonart^7^, Avner Reshef^8^, Giuseppe Spadaro^9^, Maria Staevska^10^, Marcin Stobiecki^11^, Anna Valerieva^10^, Justin Sun^12^, Li Zhu^12^, Ming Yu^12^, Giorgio Giannattasio^13^, Peng Lu^12^, Marcus Maurer^14,15^

#### ^1^Université de Montréal, CHU de Montréal, Montréal, QC; ^2^University Hospital Frankfurt, Goethe University Frankfurt, Department for Children and Adolescents, Frankfurt, Germany; ^3^Grenoble Alpes University, Laboratoire T-RAIG, UMR 5525 TIMC-IMAG (UGA-CNRS), National Reference Center for Angioedema (CREAK), Department of Internal Medicine, Grenoble, France; ^4^Semmelweis University, Department of Internal Medicine and Haematology, Hungarian Angioedema Center of Reference and Excellence, Budapest, Hungary; ^5^Sorbonne Université, Médecine Interne, AP-HP, Centre de référence des angiœdèmes à kinines, Hôpital Saint-Antoine, Paris, France; ^6^St. Anne’s University Hospital in Brno and Faculty of Medicine, Department of Clinical Immunology and Allergology, Masaryk University, Brno, Czech Republic; ^7^Bellvitge University Hospital, Allergology Service, L’Hospitalet de Llobregat, Barcelona, Spain; ^8^Barzilai University Hospital, Allergy, Immunology and Angioedema Center, Ashkelon, Israel; ^9^University of Naples Federico II, Department of Translational Medical Sciences and Center for Basic and Clinical Immunology Research (CISI), Napoli, Italy; ^10^University Hospital Alexandrovska, Department of Allergology, Clinic of Allergology, Medical University of Sofia, Sofia, Bulgaria; ^11^Jagiellonian University Medical College, Department of Clinical and Environmental Allergology, Krakow, Poland; ^12^Pharvaris Inc., Lexington, MA, USA; ^13^Pharvaris GmbH, Zug, Switzerland; ^14^Charité—Universitätsmedizin Berlin, Institute of Allergology, Corporate Member of Freie Universität Berlin and Humboldt-Universität zu Berlin, Berlin, Germany; ^15^Fraunhofer Institute for Translational Medicine and Pharmacology ITMP, Immunology and Allergology, Berlin, Germany

##### **Correspondence:** Hugo Chapdelaine

*Allergy, Asthma & Clinical Immunology* 2025, **21(Suppl 1)**:118

**Background:** Deucrictibant is a selective, orally-administered antagonist of the bradykinin B2 receptor under development for on-demand and prophylactic treatment of hereditary angioedema (HAE) attacks. In the RAPIDe-1 Phase 2 trial (NCT04618211), deucrictibant immediate-release (IR) capsule reduced time to onset of symptom relief and to resolution of HAE attacks vs placebo; treatment was well-tolerated.

**Methods**: RAPIDe-2 (NCT05396105) is an ongoing 2-part Phase 2/3 open-label extension study evaluating long-term efficacy and safety of deucrictibant IR capsule for treatment of HAE attacks. Part A enrolls adult (≥ 18 years) participants who completed RAPIDe-1. Participants continue self-administering the same double-blinded dose of deucrictibant IR capsule (10 mg, 20 mg, or 30 mg) received in RAPIDe-1 to treat qualifying non-laryngeal attacks as well as laryngeal attacks presenting without breathing difficulties.

**Results:** This Part-A data snapshot (cut-off: 01 March 2024) included 265 qualifying attacks treated with deucrictibant IR capsule by 17 participants (combined dose-group results reported). Participants’ mean age was 43.9 years at RAPIDe-1 enrollment; 61.1% were female. Median (95% CI) time to onset of symptom relief, defined as PGI-C “a little better”, was 1.1 h (1.0–1.2), with 98.5% of attacks achieving onset of symptom relief by 12 h. Median (95% CI) time to reduction in attack severity, measured as PGI-S ≥ 1 point reduction, and to complete resolution, measured as PGI-S “none”, was 2.6 (2.0–2.9) and 11.5 (11.0–13.0) hours, respectively. In total, 84.5% of attacks achieved complete resolution within 24 h, with 90.2% of these attacks achieving this milestone with a single dose of deucrictibant IR capsule. Deucrictibant IR capsule was well-tolerated with no new safety signals observed.

**Conclusions:** Results of the RAPIDe-2 extension study provide additional evidence on the long-term efficacy and safety of deucrictibant IR capsule for treatment of HAE attacks. Most attacks achieved onset of symptom relief and complete resolution with a single dose of deucrictibant IR capsule.

## 119 Long-term safety and efficacy of oral deucrictibant, a bradykinin B2 receptor antagonist, for prophylaxis in HAE: CHAPTER-1 extension study results

### William H. Yang^1^, John Anderson^2^, Francesco Arcoleo^3^, Mauro Cancian^4^, Hugo Chapdelaine^5^, Niall Conlon^6^, Efrem Eren^7^, Mark Gompels^8^, Sofia Grigoriadou^9^, Maria D. Guarino^10^, Padmalal Gurugama^11^, Tamar Kinaciyan^12^, Markus Magerl^13,14^, Michael E. Manning^15^, Marc A. Riedl^16^, Marcin Stobiecki^17^, Michael D. Tarzi^18^, Anna Valerieva^19^, H. James Wedner^20^, Andrea Zanichelli^21, 22^, Rafael Crabbé^23^, Susan Mulders^24^, Jonathan Levy^25^, Ulrich Freudensprung^26^, Umar Katbeh^26^, Jochen Knolle^27^, Anne Lesage^28^, Peng Lu^25^, Emel Aygören-Pürsün^29^

#### ^1^Ottawa Allergy Research Corporation, Department of Medicine, University of Ottawa, Ottawa, ON; ^2^AllerVie Health, Clinical Research Center of Alabama, Birmingham, AL, USA; ^3^AOR Villa Sofia-Cervello, UOC di Patologia Clinica e Immunologia, Palermo, Italy; ^4^University Hospital of Padua, Department of Systems Medicine, Padua, Italy; ^5^Université de Montréal, CHU de Montréal, Montréal, QC; ^6^St. James’s Hospital and Trinity College, Wellcome Trust CRF, Dublin, Ireland; 7. University Hospital Southampton NHS Foundation Trust, Southampton, United Kingdom; ^8^North Bristol NHS Trust, Bristol, United Kingdom; ^9^Barts Health NHS Trust, Department of Immunology, London, United Kingdom; ^10^Ospedale di Civitanova Marche, Civitanova Marche, Italy; ^11^Cambridge University Hospitals NHS Foundation Trust, Department of Clinical Immunology, Cambridge, United Kingdom; ^12^Medical University of Vienna, Department of Dermatology, Vienna, Austria; ^13^Charité – Universitätsmedizin Berlin, Institute of Allergology, Corporate Member of Freie Universität Berlin and Humboldt-Universität zu Berlin, Berlin, Germany; ^14^Fraunhofer Institute for Translational Medicine and Pharmacology ITMP, Immunology and Allergology, Berlin, Germany; ^15^Allergy, Asthma and Immunology Associates, Ltd., Scottsdale, AZ, USA; ^16^University of California San Diego, Division of Allergy and Immunology, La Jolla, CA, USA; ^17^Jagiellonian University Medical College, Department of Clinical and Environmental Allergology, Krakow, Poland; ^18^University Hospitals Sussex NHS Foundation Trust, Department of Respiratory Medicine, Brighton, United Kingdom; ^19^University Hospital Alexandrovska, Department of Allergology, Clinic of Allergology, Medical University of Sofia, Sofia, Bulgaria; ^20^Washington University School of Medicine, Division of Allergy and Immunology, Department of Medicine, St. Louis, MO, USA; ^21^Universita degli Studi di Milano, Dipartimento di Scienze Biomediche per la Salute, Milan, Italy; ^22^I.R.C.C.S., Policlinico San Donato, Centro Angioedema, UO Medicina, Milan, Italy; ^23^RC Consultancy, Bassins, Switzerland; ^24^Mulders Clinical Consulting, Groesbeek, Netherlands; ^25^Pharvaris Inc., Lexington, MA, USA; ^26^Pharvaris GmbH, Zug, Switzerland; ^27^JCK Consult, Frankfurt, Germany; ^28^GrayMatters Consulting, Schilde, Belgium; ^29^University Hospital Frankfurt, Department for Children and Adolescents, Goethe University Frankfurt, Frankfurt, Germany

##### **Correspondence:** William H. Yang

*Allergy, Asthma & Clinical Immunology* 2025, **21(Suppl 1)**:119

**Background:** Deucrictibant is a selective, orally-administered antagonist of the bradykinin B2 receptor under development for on-demand and prophylactic treatment of hereditary angioedema (HAE) attacks. CHAPTER-1 (NCT05047185) is a 2-part Phase 2 study evaluating the efficacy and safety of deucrictibant for long-term prophylaxis of HAE attacks. In the double-blind, placebo-controlled part 1 (N = 34 enrolled), the monthly attack rate was reduced by 84.5% (*P* = 0.0008) in participants receiving deucrictibant 40 mg/day (immediate-release capsule formulation; least squares mean [LSM]: 0.30; 95% CI: 0.11, 0.82) for 12 weeks vs placebo (1.94; 1.31, 2.87). In the ongoing part 2, participants receive open-label treatment with deucrictibant 40 mg/day for long-term safety and efficacy assessments.

**Methods**: Eligible participants were aged ≥ 18 and ≤ 75 years, diagnosed with HAE-1/2, not receiving other prophylactic treatments at screening, and experienced ≥ 3 attacks within 3 months prior to screening or ≥ 2 attacks during screening (up to 8 weeks). All 30 participants who completed the double-blind placebo-controlled part 1 after randomizing into treatment groups with deucrictibant 20 mg/day (N = 11) or 40 mg/day (N = 10) or with placebo (N = 9), enrolled into the ongoing part 2.

**Results:** This part 2 data snapshot (cut-off: June 2024) included 30 participants with a mean exposure to deucrictibant 40 mg/day of longer than 12 months (up to a maximum of approximately 20 months). Mean age was 39.1 years at CHAPTER-1 part 1 randomization; 60.0% were female. Treatment with deucrictibant was well-tolerated, with no TEAEs leading to treatment discontinuation. The median monthly rate of “all” and of “moderate and severe” investigator-confirmed attacks with deucrictibant 40 mg/day in part 2 was 0.00 and 0.00, respectively.

**Conclusions:** Results of the ongoing CHAPTER-1 open-label extension study provide evidence on the long-term safety and efficacy of deucrictibant for prevention of HAE attacks.

## 120 Comparative analysis of hereditary angioedema activity score (hae-as) and angioedema control test (AECT) scores

### Jack Borle^1^, Bruce Ritchie^2^, Adil Adatia^3^

#### ^1^Faculty of Medicine, University of Alberta, Edmonton, AB; ^2^Division of Hematology, Department of Medicine, University of Alberta, Edmonton, AB; ^3^Division of Pulmonary Medicine, Department of Medicine, University of Alberta, Edmonton, AB

##### **Correspondence:** Jack Borle

*Allergy, Asthma & Clinical Immunology* 2025, **21(Suppl 1)**:120

**Background:** Hereditary angioedema (HAE) is a rare genetic condition that leads to swelling of the upper airway, gastrointestinal tract, genitals, and limbs. International guidelines recommend using validated patient-reported outcome (PROM) instruments to track patient disease control and treatment response. The Angioedema Control Test (AECT) measures the level of disease control; scores range from 0 to 16, with higher values indicating better control and scores ≥ 10 indicating well-controlled disease[1]. The Disease Activity Scale for Hereditary Angioedema (HAE-AS) measures attack activity and impairment; scores range from 0 to 29, with higher values indicating greater HAE activity and scores ≤ 12 indicating mild disease[2].

**Methods**: We conducted a retrospective analysis of HAE type 1 and type 2 patients at the Edmonton Angioedema Center of Reference and Excellence (ACARE) who had both the AECT and HAE-AS scores measured concomitantly. Demographics, clinical data, and PROM scores were collected from patient charts. Descriptive statistics and Spearman’s rank-order correlation were used for analysis.

**Results:** There were n = 17 patients included in the study. The mean age was 38.1 years (SD 11.7), and 87.5% were female. The median AECT core was 13.5, indicating good control and the median HAE-AS was 6, indicating mild disease activity. Spearman’s rank-order analysis showed a non-significant correlation between AECT and HAE-AS scores (rs = 0.480, p = 0.0511).

**Conclusions:** Though disease activity and control are related concepts, this small study showed no significant correlation between the AECT and HAE-AS, suggesting these instruments capture independent information. Using both instruments together may provide a more comprehensive patient assessment.

**References:**

1. Weller K, Donoso T, Magerl M, et al. Development of the Angioedema Control Test-A patient-reported outcome measure that assesses disease control in patients with recurrent angioedema. Allergy. 2020;75(5):1165–1177.
2. Forjaz MJ, Ayala A, Caminoa M, Prior N, Pérez-Fernández E, Caballero T; DV-HAE-QoL Study Group. HAE-AS: A Specific Disease Activity Scale for Hereditary Angioedema With C1-Inhibitor Deficiency. J Investig Allergol Clin Immunol. 2021;31(3):246–252.

## 121 Comparing medications in management of chronic spontaneous urticaria in children in a prospective registry cohort

### Magan Solomon^1^, Michael Aw^2^, Conor Prosty^2^, Greg Shand^3^, Elena Netchiporouk^4^, Victoria Cook^5^, John Sampalis^6^, Michael Fein^7^, Moshe Ben-Shoshan^3^

#### ^1^Faculty of Medicine, McGill University, Montreal, QC; ^2^Faculty of Medicine, Department of Internal Medicine, McGill University, Montreal, QC; ^3^Division of Allergy & Clinical Immunology, Montreal Children’s Hospital, McGill University, Montreal, QC; ^4^Division of Dermatology, McGill University Health Centre, Montreal, QC; ^5^Division of Allergy and Clinical Immunology, University of British Columbia, Vancouver, BC; ^6^Division of Surgical Research, Surgical Epidemiology, McGill University, Montreal, QC; ^7^Division of Adult Allergy & Clinical Immunology, Montreal, QC

##### **Correspondence:** Magan Solomon

*Allergy, Asthma & Clinical Immunology* 2025, **21(Suppl 1)**:121

**Background:** Chronic spontaneous urticaria (CSU) affects up to 1.5% of children and has considerable impact on quality of life. CSU treatment guidelines for the pediatric population are limited given sparse data on efficacy and safety of different management strategies. We aim to compare the use of medications for CSU in the pediatric population at study entry (defined as first assessment visit in allergy clinic) versus follow-up.

**Methods**: Children (age 0–18) with CSU who presented to the Montreal Children’s Hospital or a community clinic in Montreal, Quebec between 2013 and 2024 were prospectively recruited. Patient demographic and medication data at entry and follow-up were collected. Chi square test was conducted using R 4.4.0 [1].

**Results:** A total of 96 participants were included, of which 40.2% were male. The median age of CSU onset was 8 years old. At study entry, 87.5% of participants were taking antihistamines: 9.5% first-generation (diphenhydramine and hydroxyzine), 53.4% second-generation, and 29.8% both. Among those taking second-generation antihistamines, 57.1% were taking cetirizine, 12.9% ketotifen, 22.9% desloratadine, 10% rupatadine and 5.7% loratadine. Additionally, 11.5% were taking steroids and 8.3% were untreated. At follow-up, 33.3% were taking cetirizine, 3.1% bilastin, 17.7% rupatadine, 12.5% desloratadine and 16.7% ketotifen. There was a statistically significant decrease in participants taking first-generation antihistamines at follow-up (6.25%) compared to study entry (37.1%) (95% CI 0.19 to 0.43, p < 0.001). There was also a statistically significant increase in participants taking omalizumab at follow-up (16.7%) compared to study entry (0%) (95% CI 0.08 to 0.25, p < 0.001).

**Conclusions:** Our findings suggest that being followed in an allergy clinic is important for optimizing medication regimens for children with CSU, mainly decreasing the use of first-generation antihistamines.

**References:**

1. R development core team. R: A language and environmental for statistical computing. Vienna, Austria: R Foundation for Statistical Computing; 2010.

## 122 Long-term effectiveness and safety of lanadelumab in patients with hereditary angioedema from Canada: Analysis of the final data from the EMPOWER Study

### Stephen D. Betschel^1^, Hugo Chapdelaine^2,3^, Remi Gagnon^4^, M. Dawn Goodyear^5^, Paul K. Keith^6^, Ahmed El-Zoeiby^7^, Natalie Khutoryansky^8^, Daniel Nova Estepan^8^

#### ^1^Division of Clinical Immunology and Allergy, University of Toronto, Toronto, ON; ^2^Centre Hospitalier de l’Université de Montréal, Université de Montréal, Montréal, QC; ^3^Montréal Clinical Research Institute, Montréal, QC; ^4^Laval University, Centre Hospitalier Universitaire de Québec (CHUQ), Québec, QC; ^5^Division of Hematology and Hematologic Malignancies, Cumming School of Medicine, University of Calgary, Calgary, AB; ^6^McMaster University, Department of Medicine, Division of Clinical Immunology and Allergy, Hamilton, ON; ^7^Takeda Canada Inc., Toronto, ON; ^8^Takeda Development Center Americas, Inc., Lexington, MA, USA

##### **Correspondence:** Stephen D. Betschel

*Allergy, Asthma & Clinical Immunology* 2025, **21(Suppl 1)**:122

**Background:** The Phase IV observational, noninterventional, multicenter EMPOWER Study (NCT03845400) evaluated the real-world effectiveness and safety of lanadelumab in patients with hereditary angioedema (HAE) from US and Canada. This EMPOWER analysis describes lanadelumab effectiveness and safety in patients from Canada.

**Methods**: Patients with HAE Type I/II were enrolled. Analyses included patients with ≥ 1 lanadelumab dose and ≥ 1 post-baseline effectiveness assessment. Patients with < 4 and ≥ 4 lanadelumab doses before enrollment were classified as new to lanadelumab (“new patients”) and established on lanadelumab (“established patients”), respectively.

**Results:** Seven new (age [mean ± SD] 48 ± 15 years, 71% female, 100% White) and 6 established (age 43 ± 17 years, 67% female, 83% White) patients from Canada were enrolled; medical history events (comorbidities) in the 6 months before enrollment were reported in 86% of new and 67% of established patients. During the study, patients from Canada received lanadelumab for 315 ± 224 (new patients) and 536 ± 366 (established patients) days (mean ± SD). The observed HAE attack rate (mean ± SD) in new patients from Canada decreased after lanadelumab initiation (1.5 ± 2.9 attacks/month pre- and 0.3 ± 0.6 post-lanadelumab); in established patients, the observed HAE attack rate during the study was 0.1 ± 0.1 attacks/month. Most on-treatment HAE attacks were mild-to-moderate in severity (new, 94%; established, 100%) and treated with plasma-derived C1 inhibitor (new, 94%; established, 71%). One treatment-emergent adverse event (TEAE) was reported in 1 (14%) new and 10 TEAEs in 2 (33%) established patients from Canada. All TEAEs were nonsevere, nonserious, and assessed as unrelated to lanadelumab treatment. No TEAEs resulted in discontinuation from the study.

**Conclusions:** In the EMPOWER Canadian subset, HAE attack rate was reduced after lanadelumab initiation in new patients; all TEAEs were nonsevere, nonserious, and unrelated to lanadelumab. Lanadelumab effectiveness and safety in the EMPOWER Canadian subset were consistent with the EMPOWER overall population, supporting lanadelumab use and place as an effective first-line HAE long-term prophylaxis option.

## 123 Impact of chronic spontaneous urticaria on HRQoL domains in Canadian patients: results from the urticaria voices study

### Gordon Sussman^1^, Elaine Dery^2^, Stephanie Charland^3^, Patrick Leclerc^3^, Jaggi Rao^4^

#### ^1^University of Toronto, Toronto, ON; ^2^Canadian Chronic Urticaria Society, Quebec, QC; ^3^Novartis Pharmaceuticals Canada Inc., Montreal, QC; ^4^University of Alberta, Edmonton, AB

##### **Correspondence:** Gordon Sussman

*Allergy, Asthma & Clinical Immunology* 2025, **21(Suppl 1)**:123

**Background:** Chronic spontaneous urticaria (CSU), characterized by itchy wheals (hives) and/or angioedema for ≥ 6 weeks, significantly impacts patients’ health-related quality of life (HRQOL) [1–3]. Herein, we assessed the impact of CSU on HRQOL in a Canadian patients’ subset of the Urticaria Voices survey.

**Methods**: Urticaria Voices was a multinational, cross-sectional survey. Patients with CSU aged > 18 years and eligible CSU-treating physicians, respectively, completed 40- and 30-min online surveys. The perceived negative impact of CSU on 6 HRQOL domains was assessed using a 10-point scale (higher scores indicated higher impact). Data were analyzed descriptively and reported as percentage (%) or mean ± standard deviation.

**Results:** A total of 73 Canadian patients with CSU (women, 82%; age, 45 ± 11.6 years) and 40 physicians (dermatologists, 68%; allergists, 28%) participated in the study. Overall, 49% (36 of 73) of patients reported experiencing angioedema ever, and 74% (54 of 73) were currently on anti‑histamines. Patients reported the highest negative impact on the HRQOL domain of mental/emotional wellbeing (6.2 ± 3.0), followed by social life/intimate relationships (5.6 ± 3.3), activities of daily living (5.2 ± 3.1), family life (4.8 ± 3.1), professional/academic life (4.6 ± 3.2), and financial life (3.9 ± 2.8). Among the parameters assessed under mental/emotional wellbeing, patients reported being most negatively impacted by the “stress about the spontaneous nature of CSU” (60%), “desperate need to achieve relief from CSU symptoms” (41%), and “anxiety” (40%). Apart from itching and hives, patients experienced physical symptoms during an urticaria outbreak, such as sleeping problems (66%), reduced concentration (60%), fatigue (55%), and pain (51%).

**Conclusions:** CSU negatively impacts the mental and emotional wellbeing of Canadian patients, a trend that aligns with reported global patient data. Patient awareness and access to newer therapies that have a fast onset of action present an opportunity to mitigate the “stress about the spontaneous nature of CSU” and physical symptoms, thereby facilitating improved HRQOL.

**References:**

1. Maurer M, Abuzakouk M, Bérard F, Canonica W, Oude Elberink H, Giménez-Arnau A, Grattan C, Hollis K, Knulst A, Lacour JP, Lynde C, Marsland A, McBride D, Nakonechna A, Ortiz de Frutos J, Proctor C, Sussman G, Sweeney C, Tian H, Weller K, Wolin D, Balp MM. **The burden of chronic spontaneous urticaria is substantial: Real-world evidence from ASSURE-CSU**. *Allergy*. 2017;**72**:2005–2016.
2. Zuberbier T, Abdul Latiff AH, Abuzakouk M, Aquilina S, Asero R, Baker D, Ballmer-Weber B, Bangert C, Ben-Shoshan M, Bernstein JA, Bindslev-Jensen C, Brockow K, Brzoza Z, Chong Neto HJ, Church MK, Criado PR, Danilycheva IV, Dressler C, Ensina LF, Fonacier L, Gaskins M, Gáspár K, Gelincik A, Giménez-Arnau A, Godse K, Gonçalo M, Grattan C, Grosber M, Hamelmann E, Hébert J, Hide M, Kaplan A, Kapp A, Kessel A, Kocatürk E, Kulthanan K, Larenas-Linnemann D, Lauerma A, Leslie TA, Magerl M, Makris M, Meshkova RY, Metz M, Micallef D, Mortz CG, Nast A, Oude-Elberink H, Pawankar R, Pigatto PD, Ratti Sisa H, Rojo Gutiérrez MI, Saini SS, Schmid-Grendelmeier P, Sekerel BE, Siebenhaar F, Siiskonen H, Soria A, Staubach-Renz P, Stingeni L, Sussman G, Szegedi A, Thomsen SF, Vadasz Z, Vestergaard C, Wedi B, Zhao Z, Maurer M. **The international EAACI/GA**^2^**LEN/EuroGuiDerm/APAAACI guideline for the definition, classification, diagnosis, and management of urticaria**. *Allergy*. 2022;**77**:734–766.
3. Balp MM, Vietri J, Tian H, Isherwood G. The impact of chronic urticaria from the patient’s perspective: A survey in five European countries. *Patient*. 2015;**8**:551–558.

## 124 Intravenous use of concentrated C1 inhibitor for on-demand treatment of hereditary angioedema: study design of a phase 2 trial

### Ashley Holmes^1^, Bruce Ritchie^2^, Adil Adatia^3^

#### ^1^Faculty of Science, University of Alberta, Edmonton, AB; ^2^Division of Hematology, Department of Medicine, University of Alberta, Edmonton, AB; ^3^Division of Pulmonary Medicine, Department of Medicine, University of Alberta, Edmonton, AB

##### **Correspondence:** Ashley Holmes

*Allergy, Asthma & Clinical Immunology* 2025, **21(Suppl 1)**:124

**Background:** Hereditary angioedema (HAE) is a genetic disease characterized by recurrent swelling of the bowel mucosa, extremities and upper airway. Haegarda is a plasma-derived C1-inhibitor administered subcutaneously for long-term prophylaxis (LTP). Haegarda is very similar in formulation to Berinert, which is approved for on-demand treatment of angioedema attacks and is administered by IV. The possibility of treatment with Haegarda for both on-demand and prophylactic treatment presents an opportunity for both streamlined and lower-cost treatment. We report a study design for a Phase 2 study of IV Haegarda in HAE patients.

**Methods**: This pilot study is a prospective, open-label, single-arm Phase 2 clinical trial conducted to evaluate the safety and efficacy of IV Haegarda. The specific objectives of this study are to 1) assess the tolerability, 2) adverse events, 3) the effectiveness for the treatment of acute attacks, and 4) treatment satisfaction of patients using IV Haegarda. Patients will document every IV infusion of Haegarda, time to attack resolution, and adverse events during the surveillance period of 36 weeks following enrollment. Study inclusion criteria include: age ≥ 18 years, ≥ 2 angioedema episodes requiring on-demand therapy in the past 8 weeks, and no use of LTP or no changes to LTP for the past 3 months. A total of n = 18 patients will be recruited.

**Results:** Regulatory approvals are currently pending. Enrollment will begin following approval in Q4 2024. The study is expected to be completed in Q3 2025.

**Conclusions:** The results of this pilot study will serve as the first step towards assessing the tolerability and efficacy of IV Haegarda and facilitate future randomized multicentre studies.

## Data Availability

.

